# Molecular phenotyping of the surfaceome of migratory chondroprogenitors and mesenchymal stem cells using biotinylation, glycocapture and quantitative LC-MS/MS proteomic analysis

**DOI:** 10.1038/s41598-019-44957-y

**Published:** 2019-06-21

**Authors:** Csaba Matta, David J. Boocock, Christopher R. Fellows, Nicolai Miosge, James E. Dixon, Susan Liddell, Julia Smith, Ali Mobasheri

**Affiliations:** 1 Department of Anatomy, Histology and Embryology, Faculty of Medicine, University of Debrecen, Debrecen, H-4032 Hungary; 2 Department of Veterinary Preclinical Sciences, School of Veterinary Medicine, Faculty of Health and Medical Sciences, University of Surrey, Guildford Surrey, GU2 7AL United Kingdom; 3 John van Geest Cancer Research Centre, Nottingham Trent University, Nottingham, NG11 8NS United Kingdom; 4 Department of Prosthodontics, Tissue Regeneration Work Group, Georg August University, Göttingen, Germany; 5 School of Pharmacy, Wolfson Centre for Stem Cells, Tissue Engineering, and Modelling, Centre of Biomolecular Sciences, University of Nottingham, Nottingham, NG7 2RD United Kingdom; 6 School of Biosciences, University of Nottingham, Sutton Bonington, LE12 5RD United Kingdom; 7 Bruker UK Limited, Coventry, CV4 9GH United Kingdom; 8 Centre for Sport, Exercise and Osteoarthritis Research Versus Arthritis, Queen’s Medical Centre, Nottingham, NG7 2UH United Kingdom; 9 Sheikh Salem Bin Mahfouz Scientific Chair for Treatment of Osteoarthritis with Stem Cells, King Abdulaziz University, Jeddah, Saudi Arabia; 10 Department of Regenerative Medicine, State Research Institute Centre for Innovative Medicine, Vilnius, Lithuania; 11 Research Unit of Medical Imaging, Physics and Technology, Faculty of Medicine, University of Oulu, Aapistie 5 A, Oulu, FIN-90230 Finland

**Keywords:** Mechanisms of disease, Proteomics, Mesenchymal stem cells, Stem-cell differentiation

## Abstract

The complement of cell surface proteins, collectively referred to as the surfaceome, is a useful indicator of normal differentiation processes, and the development of pathologies such as osteoarthritis (OA). We employed biochemical and proteomic tools to explore the surfaceome and to define biomarkers in chondrogenic progenitor cells (CPC) derived from human OA knee articular cartilage. These cells have great therapeutic potential, but their unexplored biology limits their clinical application. We performed biotinylation combined with glycocapture and high throughput shotgun proteomics to define the surface proteome of human bone marrow mesenchymal stem cells (MSCs) and human CPCs. We prepared cell surface protein-enriched fractions from MSCs and CPCs, and then a proteomic approach was used to compare and evaluate protein changes between undifferentiated MSCs and CPCs. 1256 proteins were identified in the study, of which 791 (63%) were plasma membrane, cell surface or extracellular matrix proteins. Proteins constituting the surfaceome were annotated and categorized. Our results provide, for the first time, a repository of quantitative proteomic data on the surfaceome of two closely related cell types relevant to cartilage biology and OA. These results may provide novel insights into the transformation of the surfaceome during chondrogenic differentiation and phenotypic changes during OA development.

## Introduction

Proteins on the cell surface, collectively referred to as the *surfaceome*, help to define cellular phenotype, identity, communication and behaviour. They mediate ion and metabolite transport, cell adhesion, cell-matrix interactions, vesicle trafficking, signalling and communication, and represent a source of potential diagnostic biomarkers of various diseases and disease states^1,2^. Their biomedical importance is underpinned by the fact that two-thirds of approved human drugs target a cell surface protein^3^. Despite their key roles, proteomic approaches to study cell surface membrane proteins are challenging to develop and implement successfully. Progress in this area is hampered and complicated by the relatively low abundance and the hydrophobicity of plasma membrane (PM) proteins, and difficulties associated with their purification and separation from membrane proteins residing in cell organelles^4,5^. To overcome these challenges, techniques that both enrich the PM fraction and improve the level of peptide detection during mass spectrometry need to be developed and refined. Cell surface proteins can either be selectively isolated via chemical labels or extracted as part of the entire PM fraction based on their physicochemical (amphiphilic) properties. As the majority of the extracellular and secreted proteins are glycosylated, the orientation and ubiquity of carbohydrate moieties on surface proteins provide a unique opportunity to enrich membrane proteins, which is generally termed as “glycocapture”^6^. Whilst there have been conflicting reports with regards to the efficiency of such enrichment techniques, both in terms of the yield and purity of PM proteins and the type of non-plasma membrane annotated proteins that are co-purified^7^, this method has yielded the highest relative amount of PM proteins in an earlier study^4^.

The aim of the current study was to apply the glycocapture biotinylation technique, which is based on the selective labelling of sialylated glycoproteins with aminooxy-biotin^5^, to characterise the surfaceome of mesenchymal (stromal) stem cells (MSCs) and chondroprogenitor cells (CPCs), which have great potential in cartilage tissue engineering and may have clinical applications in cartilage repair. Cartilage has a poor intrinsic repair capacity; critical size defects arising as a result of joint injuries rarely heal and in the long-term have the potential to lead to osteoarthritis (OA) in the affected joint. Therefore, interventions are often required as an attempt to repair damaged cartilage^8^. The most widely used surgical method for cartilage repair involves penetration of the subchondral bone, which causes the release of multipotent bone marrow-derived mesenchymal stem cells (BM-MSCs). MSCs in turn differentiate into chondrocytes^9^ and secrete a proteoglycan-rich extracellular matrix as an attempt to regenerate the damaged area^10^. However, the fibrous repair tissue formed has inferior biomechanical features compared to hyaline cartilage^11^. Also, chondrogenically induced BM-MSCs hold the inherent risk of undergoing terminal differentiation to form calcifying cartilage, subchondral bone overgrowth or intra-lesional osteophytes. The success of BM-MSC-based techniques in hyaline articular cartilage regeneration may remain limited if the presence of fibrous and hypertrophic tissue cannot be eliminated^12^.

Tissue-specific MSCs have now been isolated from healthy and OA adult articular cartilage^13–15^. Compared to bone marrow and adipose-derived MSCs, articular cartilage-derived progenitor cells (CPCs) are believed to be further along in their commitment to the chondrogenic lineage primed to form hyaline cartilage, which makes them a logical choice for articular cartilage tissue engineering^8^. Existing resident stem cell populations within healthy or OA adult articular cartilage offer a novel and promising cell source for tissue engineering. The discovery and characterisation of reliable cell surface markers and isolation methods is essential in ensuring the most effective repair. However, comprehensive identification of cell-specific surface markers for CPCs has been limited by a lack of suitable methodology for the enrichment of cell surface proteins, and inability to compare protein levels between stem cells, progenitors and differentiated cells^16^. Therefore, in the current study we employed the aminooxy-biotinylation strategy followed by shotgun mass spectrometry to enrich and characterise the surfaceome of CPCs and BM-MSCs. This process enables the study of similarities and differences in the surfaceome of these cells and discover potential biomarkers that would facilitate their selective isolation.

## Results

### The aminooxy-biotinylation technique resulted in a high relative abundance of cell surface proteins

**Figure 1:**
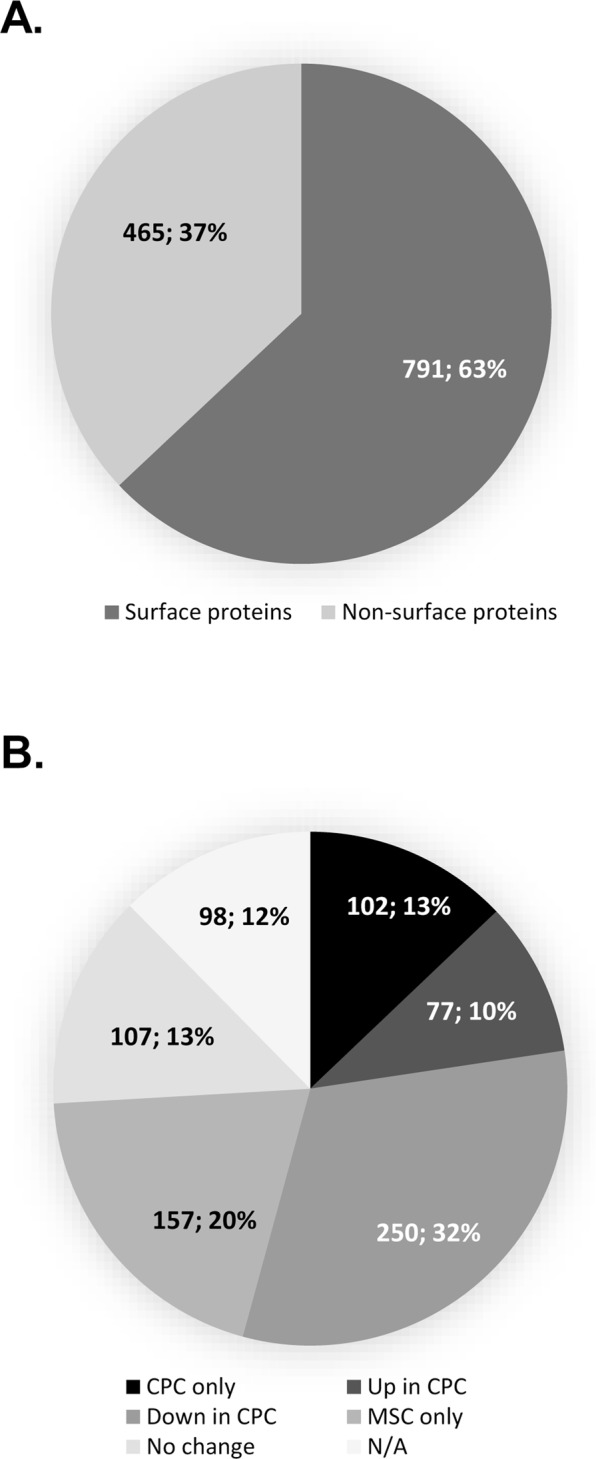
(**A**) Subcellular distribution of the surfaceome proteins identified following glycocapture. The surfaceome comprises the subproteome with proteins annotated as “plasma membrane”, “cell surface”, “cell membrane”, “extracellular” or “secreted”, as well as their associated proteins. (**B**) Surface proteins were further classified according to their presence/absence and relative expression in CPC and MSC, based on normalised quantitative data (cut-off: ±1.5 FC). Numbers in pie charts represent the actual numbers and relative percentages of proteins identified in each subgroup using all data from the PEAKS Studio protein identification export.

In line with previous studies^3,4,17^, those proteins that constitute the surfaceome fraction were defined as the subpopulation of proteins annotated as “plasma membrane”, “cell surface”, “cell membrane”, “extracellular” or “secreted”. The glycocapture method with aminooxy-biotin yielded a very high relative amount of surfaceome proteins compared to non-surfaceome proteins; of the 1256 proteins reliably identified, 791 proteins (63%) were considered as surfaceome proteins according to their gene ontology (GO) annotations (Fig. 1A). This included those proteins that are known interacting partners with the cell surface subproteome but fulfilled the above GO annotation criteria. Based on normalised quantitative data, we also looked at the relative expression of proteins between CPC and MSC. Not all 791 surface proteins were identified in both cell types; whilst the majority (434 proteins; 55%) of proteins were detected in both CPC and MSC, 102 proteins (13%) were unique to CPC and 157 proteins (20%) were unique to MSC. For 98 proteins (12%), we were unable to determine fold change (FC) directions or magnitudes. This shows that despite similarities, the two cells are characterised by distinct surfaceomes. Of the 434 proteins identified in both cell types, 77 proteins (10%) were upregulated in CPC, 250 proteins (32%) were showing lower protein levels in CPC, and there were no significant differences (below the cut-off threshold of ±1.5 FC) in the expression of 107 (13%) proteins between CPC and MSC (Fig. 1B).

### Validation of surface CD antigen expression profiles for CPC and MSC

Given that MSC cultures are a heterogeneous population of cells, no unique cell surface markers are available for their identification and isolation, and a panel of positive and negative cluster of differentiation (CD) markers are used as selection criteria. Cells must be positive for the commonly expressed surface proteins including CD29, CD44, CD49a–f, CD51, CD73, CD90, CD105, CD106, CD166 and Stro-1, and must be negative for hematopoietic lineage markers including CD11b, CD14, and CD45^18^. According to the recommendation of The International Society of Cellular Therapy, the minimum criteria for MSC must include adhesion to plastic, expression of surface markers CD73, CD90, CD105 (≥95% expression), the absence of hematopoietic markers CD34, CD45, CD14 or CD11b, CD79a or CD19 (≤2%), the absence of HLA Class II molecules, and tripotent differentiation into chondrogenic, osteogenic, and adipogenic phenotypes^19,20^.

To this end, we first investigated the above MSC surface markers to validate both the methodology that we were using and the phenotype of cells. The two cell types shared a remarkably similar CD surface antigen expression profile (Table 1). Both MSC and CPC expressed the following mesenchymal progenitor markers: 5′-nucleotidase (CD73), CD166 antigen, CD44 antigen, endoglin (CD105), integrin alpha-1 (CD49a), integrin alpha-2 (CD49b), integrin alpha-3 (CD49c), integrin alpha-4 (CD49d), integrin alpha-5 (CD49e), integrin alpha-6 (CD49f), integrin alpha-V (CD51), integrin beta-1 (CD29), Thy-1 membrane glycoprotein (CD90) and vascular cell adhesion protein 1 (CD106), but no expression of the hematopoietic markers CD34, CD45, CD14, CD79a or CD19 was detected, as expected. CD49b (integrin alpha-2) exhibited higher signal intensities in CPC than in MSC. 5′-nucleotidase (CD73), integrin alpha-5 (CD49e) and CD49f (integrin alpha-6) were present at equal amounts, and the other markers showed lower scores in CPC. Thus, we confirmed that the applied methodology was appropriate and sensitive enough to detect conventional cell surface markers, and that both cell types fulfilled the criteria of mesenchymal stem cells.

**Table 1:** MSCs and CPCs fulfilled the minimum criteria for MSCs according to the recommendation of The International Society of Cellular Therapy in terms of cell surface CD marker expression. Relative expression levels in CPC *vs*. MSC are based on normalised quantitative MS data (cut-off for significant up/down regulation was ± 1.5 FC).

Accession	Protein ID	Description	−10lgP	Area CPC	Area MSC	Fold change	Expression
P21589	5NTD_HUMAN	5′-nucleotidase (CD73)	325.74	6 110 000	6 340 000	−1.04	Unchanged
Q13740	CD166_HUMAN	CD166 antigen	355.25	19 500 000	45 000 000	−2.31	Down in CPC
P16070	CD44_HUMAN	CD44 antigen	293.92	62 700 000	96 000 000	−1.53	Down in CPC
P17813	EGLN_HUMAN	Endoglin (CD105)	316.98	2 270 000	14 400 000	−6.34	Down in CPC
P56199	ITA1_HUMAN	Integrin alpha-1 (CD49a)	254.73	1 010 000	3 630 000	−3.59	Down in CPC
P17301	ITA2_HUMAN	Integrin alpha-2 (CD49b)	317.04	8 960 000	2 160 000	4.15	Up in CPC
P26006	ITA3_HUMAN	Integrin alpha-3 (CD49c)	338.92	9 950 000	22 500 000	−2.26	Down in CPC
P13612	ITA4_HUMAN	Integrin alpha-4 (CD49d)	123.76	90 300	272 000	−3.01	Down in CPC
P08648	ITA5_HUMAN	Integrin alpha-5 (CD49e)	379.98	22 700 000	23 100 000	−1.02	Unchanged
P23229	ITA6_HUMAN	Integrin alpha-6 (CD49f)	278.4	2 610 000	2 530 000	1.03	Unchanged
P06756	ITAV_HUMAN	Integrin alpha-V (CD51)	358.52	12 500 000	22 600 000	−1.81	Down in CPC
P05556	ITB1_HUMAN	Integrin beta-1 (CD29)	334.1	33 200 000	50 100 000	−1.51	Down in CPC
P04216	THY1_HUMAN	Thy-1 membrane glycoprotein (CD90)	173.3	3 320 000	7 040 000	−2.12	Down in CPC
P19320	VCAM1_HUMAN	Vascular cell adhesion protein 1 (CD106)	281.07	10 500	3 940 000	−375.24	Down in CPC

### Functional categories of annotated proteins

**Figure 2:**
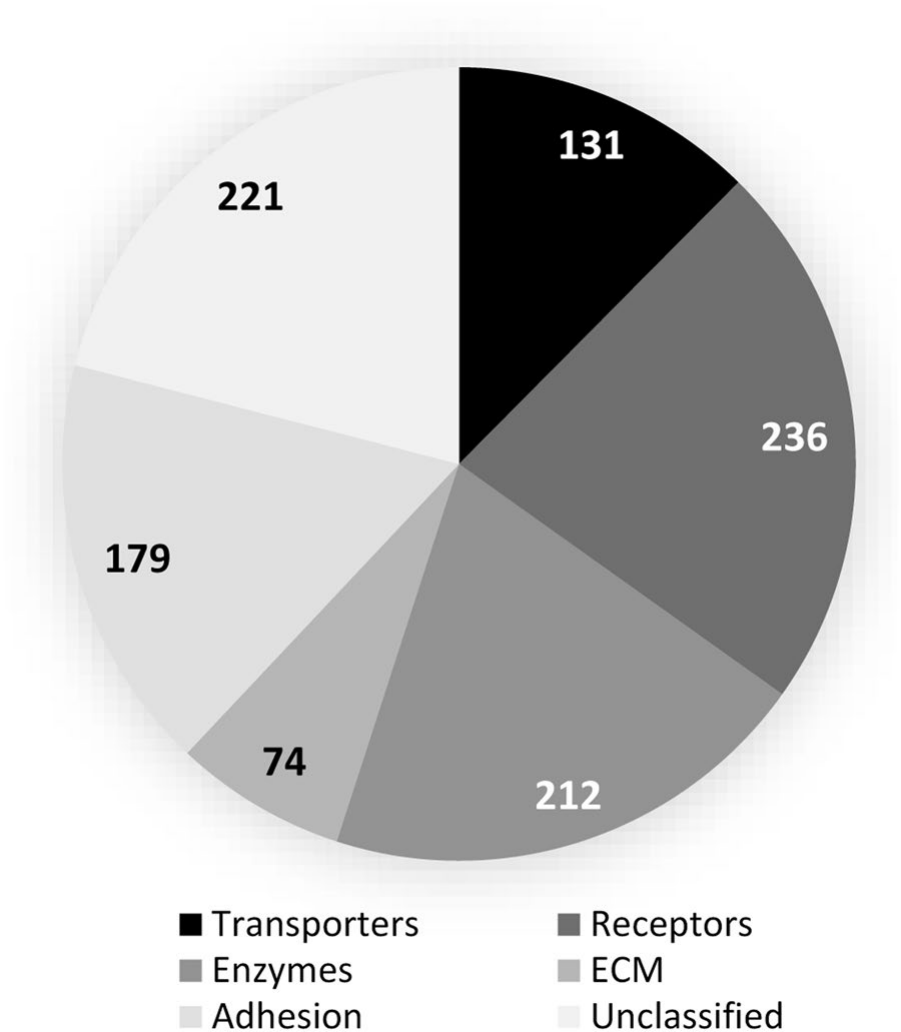
Classification of the identified 791 surface proteins in both CPCs and MSCs (combined). Surface proteins were categorised according to their GO annotations in the UniProt database (GO molecular function and/or GO biological process) into the following major functional groups: transporters, receptors, enzymes, extracellular matrix components, proteins involved in cell adhesion and cell junctions, and unclassified proteins. Note that some proteins appear in more than one category. Numbers in the pie chart represent the actual numbers of proteins classified into each subgroup.

Based on the classification criteria presented by Almén and colleagues^21^ and Bausch-Fluck and colleagues^3^ we classified the surfaceome proteins identified in this study into the following major functional groups: transporters, receptors, enzymes, extracellular matrix components, proteins mediating cell junctions and adhesion, and unclassified proteins (Fig. 2). Classification was based on the Gene Ontology (GO) annotations (GO molecular function and/or GO biological process) in the UniProt database using the keywords given under each category. The full lists of proteins in each subcategory can be found in Supplementary Tables S1–S6.

**Figure 3:**
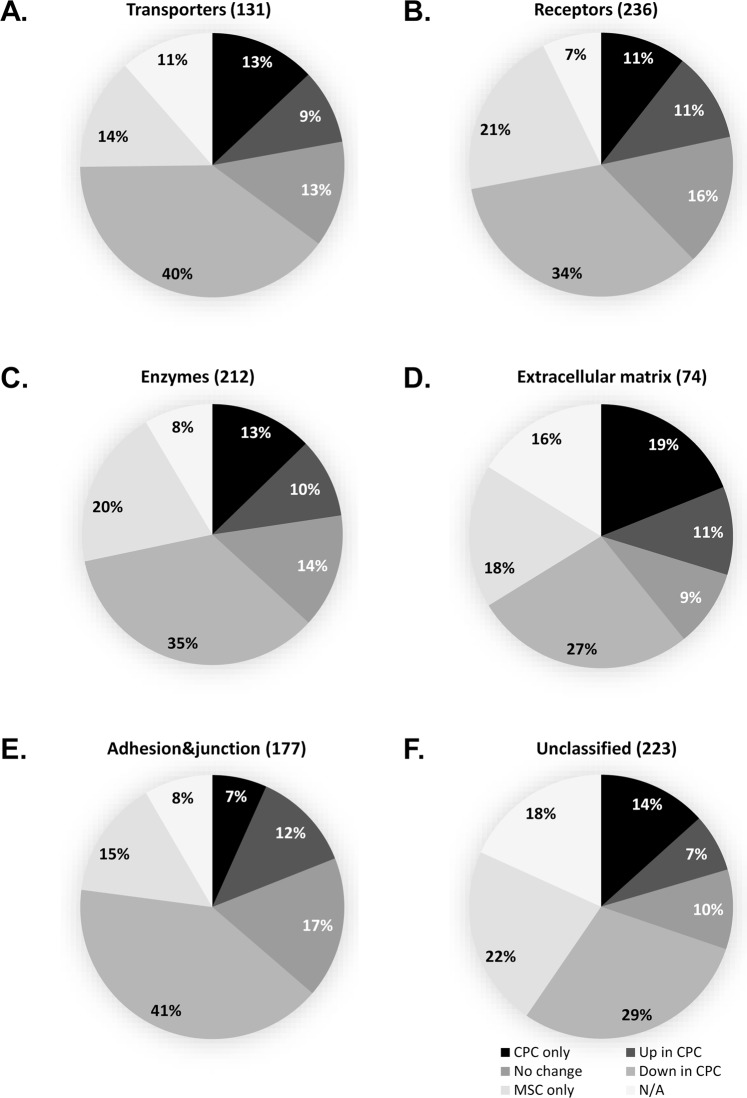
Pie charts showing the differential expression of proteins in CPCs and MSCs in all 6 functional protein groups (cut-off: ±1.5 FC). Numbers in the pie charts represent the relative percentages of proteins in each subgroup using all data from the PEAKS Studio protein identification export.

1. *Transporters* are involved in enabling the movement of a substrate (ion or solute) across membranes by utilizing electrochemical gradients or energy from chemical reactions. We put those proteins into this category which contained the following terms in their GO annotations: ‘transporter,’ ‘symporter,’ ‘antiporter,’ ‘channel,’ ‘porin,’ and ‘exchanging’. We identified 131 transporters in the two cell types (Fig. 3A, see Supplementary Table S1). Members of many key channel and transporter groups were identified and some of these were differentially expressed on the two cell types. Interesting distribution was found with regards to the voltage-dependent calcium channels; CAC1A (voltage-dependent P/Q-type calcium channel subunit alpha-1A) was detected in MSC only, whereas the CAC1H (voltage-dependent T-type calcium channel subunit alpha-1H) alpha subunit was identified in CPC only. On the other hand, the plasma membrane calcium-transporting ATPase 3 (AT2B3), as well as voltage-dependent anion-selective channel proteins 1 and 3 (VDAC1 and VDAC3) were expressed in MSC only. Potassium, sodium and chloride channels, as well as non-selective cation channels were also showing differential distribution; e.g. the big conductance calcium-activated potassium channel subunit alpha-1 (KCMA1) was upregulated in CPC, whereas the transient receptor potential cation channel subfamily M member 2 (TRPM2), the inward rectifier potassium channel 2 (KCNJ2) and potassium voltage-gated channel subfamily KQT member 2 (KCNQ2) could only be detected in CPC. Interestingly, TRPM4 was only found in MSC.
2. *Receptors* are proteins that mediate a cellular response following ligand binding. Based on the keywords ‘receptor,’ ‘collagen binding’ and ‘integrin,’ we classified 236 proteins as receptors in the surfaceome of CPC and MSC (Fig. 3B, see Supplementary Table S2). Using the above keywords, we have also picked up proteins which are interacting with receptor proteins, such as heat shock 70 kDa protein 1 A (HS71A), a molecular chaperone with a receptor binding activity. More than 50% of these proteins were either downregulated or were unique to MSC (or under the detection threshold in CPC); for example, ADRB2 (beta-2 adrenergic receptor), BKRB2 (B2 bradykinin receptor), BMR1A (bone morphogenetic protein receptor type-1A), integrin alpha-X and VGFR3 (vascular endothelial growth factor receptor 3) were only detected in MSC. On the contrary, the receptor-type tyrosine-protein phosphatases beta and mu (PTPRB and PTPRM), as well as syntaxin-4 were only found in CPC.
3. *Enzymes* are proteins with the ability to catalyse a chemical reaction. We have identified 212 surfaceome-associated enzymes in this study, based on GO annotations containing the term ‘enzymatic activity’ (Fig. 3C, see Supplementary Table S3). Interesting differences were found between the two cell types investigated in this study with regards to enzymes in or associated with the surfaceome. For example, we identified adenylate cyclase types 1, 3, 7 and 9; of these, ADCY1, 3 and 7 were present in both cell types but ADCY9 could only be detected in MSC. Bone morphogenetic protein receptor type-1A (BMR1A), as well as PPBT, the tissue-nonspecific isozyme of alkaline phosphatase were also only detected in MSC. ALK tyrosine kinase receptor and carbonic anhydrase 12, on the other hand, was specific to CPC.
4. *Extracellular matrix (ECM) structural components* are proteins with the following subcellular locations: ‘secreted,’ ‘extracellular space,’ and/or ‘extracellular matrix’. We found 74 proteins with such annotations (Fig. 3D, see Supplementary Table S4). For collagen alpha chains, CO2A1 (collagen type II), CO6A6 (collagen type VI), CONA1 (collagen type XXIII) and CORA1 (collagen type XXVII) were CPC-specific, whereas CO4A5 and CO4A6 (collagen type IV), CO5A1 (collagen type V), COJA1 (collagen type XIX) and COOA1 (collagen type XXIV) were only present in MSCs. The cell surface proteoglycan syndecan-3, as well as agrin and filaggrin were only identified in CPC.
5. We noticed that protein entries with GO annotations related to cell junctions, cell adhesions and the cytoskeleton were overrepresented in the list of reliably identified proteins, therefore we created an arbitrary list of proteins with the following GO annotations: ‘cell-cell junction,’ ‘adherens junction,’ ‘focal adhesion’ and ‘cytoskeleton’. A total of 179 proteins were mapped to the list of Structural/Adhesion/Junctional proteins (Fig. 3E, see Supplementary Table S5). In this group, the leucine-rich repeat transmembrane protein FLRT2 and plakophilin-4 were only identified in CPC. On the other hand, the leucine-rich repeat-containing protein 7 (LRRC7) and four and a half LIM domains protein 1 (FHL1) were present in MSC only. Integrins alpha-1, 3, 4, 7, 11 and V, as well as beta-1 and 3 were downregulated, and integrin alpha-2 was upregulated in CPC.
6. We identified 225 proteins that have not been categorised into any of the 5 major functional classes and we put them into an *Unclassified* group (Fig. 3F, see Supplementary Table S6). Proteins in this group were involved in various biological processes, including HLA class histocompatibility antigens or protocadherins, and, as might be expected from any proteomic study of cultured cells, “contaminant” proteins such as keratins, trypsin or albumin are also included in this list. The bone marrow stromal antigen 2 (BST2) was detected in both cell types but with higher MS scores and peptide coverage in MSC, possibly reflecting on the bone marrow stromal origin of both CPC and MSC.

### Differentially expressed proteins

**Figure 4:**
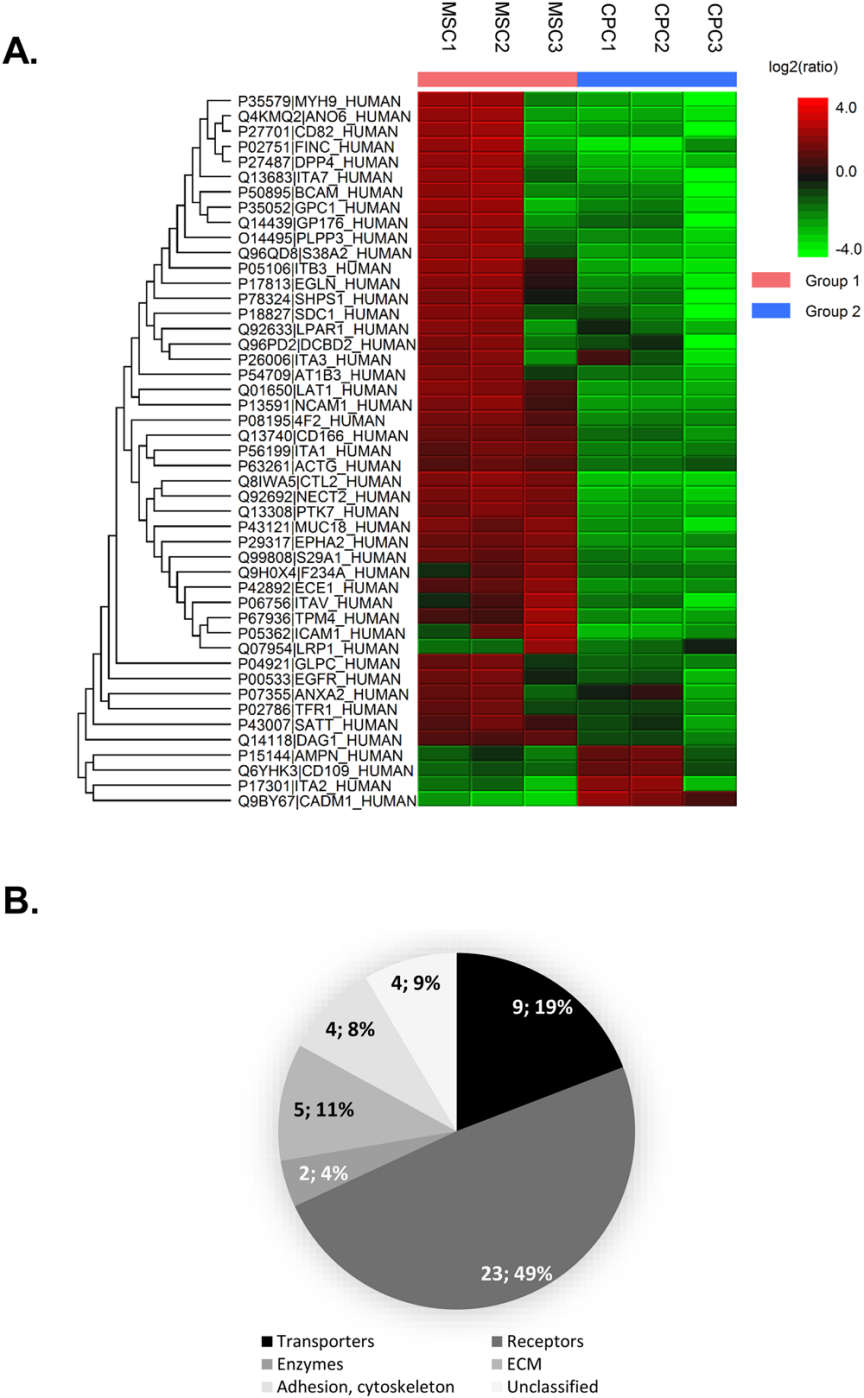
(**A**) Protein profile heatmap generated from PEAKS Studio quantitation module, showing significantly differentially expressed proteins following quantitative LC-MS/MS analysis (cut-off fold change >1.5) utilising the Top 3 peptides from each protein. Cell colour represents the log2(ratio) to the average area across different samples. (**B**) Functional classification of significantly differentially expressed surface proteins. Surface proteins were categorised according to their GO annotations in the UniProt database into the major functional groups. Numbers in the pie chart represent the actual numbers and percentages of proteins classified into each subgroup.

Following quantitative analysis of the liquid chromatography-tandem mass spectrometry (LC-MS/MS) data between CPCs and MSCs, and utilising the Top 3 peptides from each protein, a protein profile heatmap was generated (Fig. 4A). 4 proteins were found with significantly increased expression in CPCs – AMPN (Aminopeptidase N), CD109 antigen, CADM1 (cell adhesion molecule 1) and ITA2 (integrin alpha-2). 43 proteins showed significantly higher expression in MSCs; significantly differentially expressed proteins had the following functional classification: 9 transporters, 23 receptors, 2 enzymes, 5 extracellular matrix components, 4 structural/adhesion/cytoskeletal proteins and 4 unclassified proteins (Fig. 4B, see also Supplementary Table S7 in the Supporting Information; further details of these proteins are contained in the Excel spreadsheet available as a Supplementary Dataset).

### Validation of KCNMA1 protein distribution

**Figure 5:**
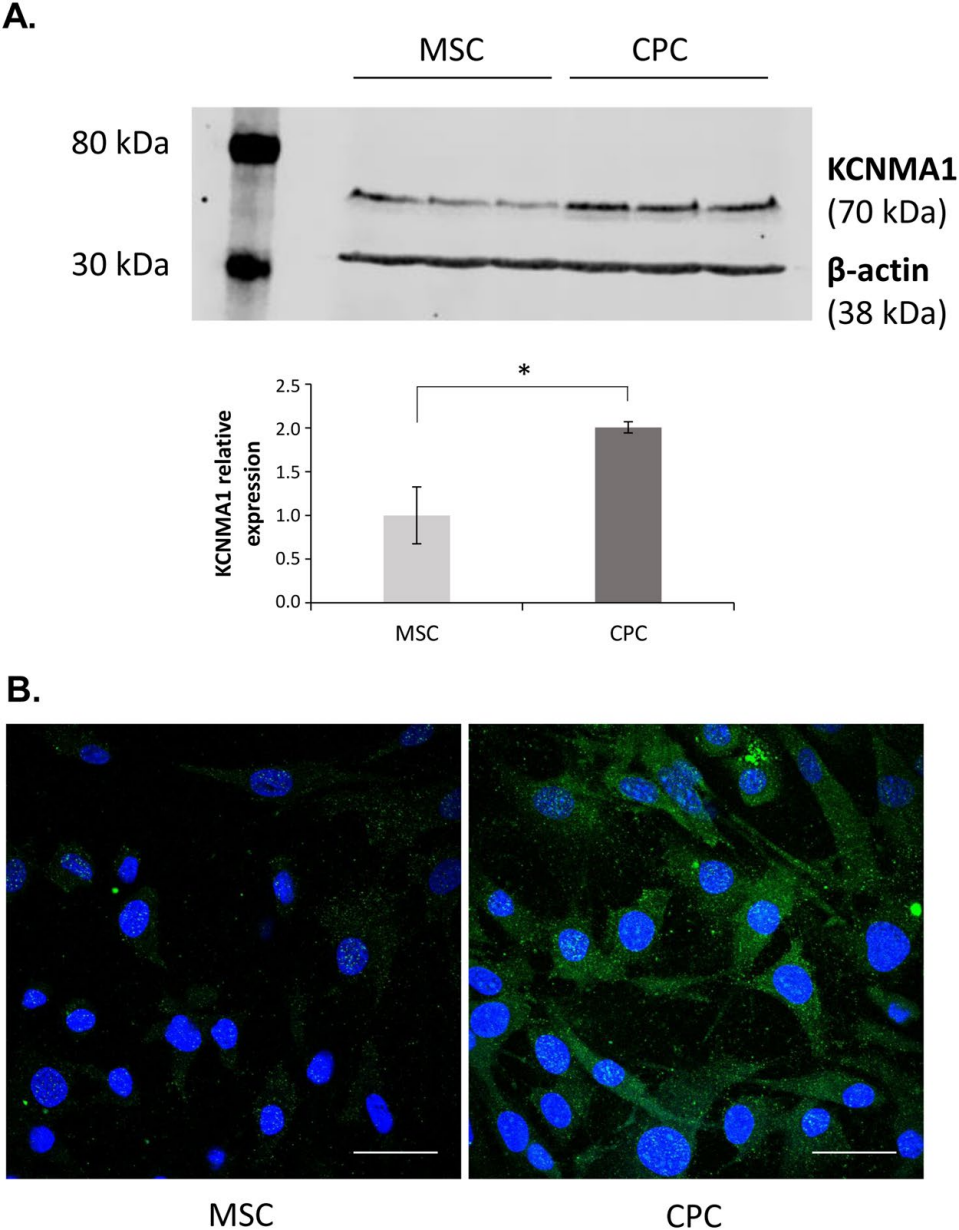
Validation of the efficacy of the aminooxy-biotin based glycocapture method. (**A**) Western blot experiment performed on total cell lysates from human CPC and MSC cultures. After SDS–PAGE, protein bands were visualised using the Odyssey FC imaging system (Li-Cor) and bands were detected using the 800 nm (for KCNMA1) and 700 nm (for β-actin) channels. Optical density values of detected bands were calculated using the Image Studio software and the values for KCNMA1 were normalised to those of β-actin and then to MSC. Representative gel image. Full-length uncropped blots are presented in Fig. S2. Error bars are ± SD, *n* = 3. * indicates statistical significance (*P* < 0.05). (**B**) Intracellular distribution of KCNMA1 in MSCs and CPCs detected by immunocytochemistry. Primary antibodies were visualised using Alexa488-conjugated anti-rabbit secondary antibodies. Nuclear DNA was stained with DAPI. Data shown are representative out of 3 independent experiments. Scale bar, 50 µm.

To validate the efficacy of the aminooxy-biotin method, we have chosen the calcium-activated potassium channel subunit alpha-1 (KCNMA1), a protein with a differential expression pattern in these two cell types (3.4-fold higher expression in CPC). We performed immunocytochemical staining on CPCs and MSCs and studied its expression using western blots of total cell lysates (Fig. 5). Full-length uncropped blots are presented in Supplementary Fig. S2. Both methods have confirmed a lower level of protein in MSCs compared to CPCs; the protein levels in CPCs were two times higher (2.00 ± 0.032; *P* = 0.019) than in MSCs as revealed by western blot analyses, with a very high effect size (Cohen’s d = 3.32). These data correlate well with the quantitative LC-MS/MS analysis.

## Discussion

The aim of this study was to compare and contrast the surfaceomes of MSCs and CPCs using quantitative proteomics. We employed biotinylation and glycocapture together with high throughput shotgun quantitative proteomics to define the surface proteome of these cells. The rationale for this study was to fine-tune an efficient labelling technique to enrich the surfaceome and learn more about the cell biology of MSCs and CPCs.

The discovery of resident articular cartilage progenitor cells (CPCs) in both healthy and OA cartilage characterised by a high chondrogenic potential has opened up new possibilities for the future treatment of cartilage and joint pathologies including OA^22,23^. However, despite the significant advances made in this field over the last 10 years, the detailed characterisation of these cells has still not been completed. Given that the percentage of CPCs in articular cartilage is very low (1.5% in healthy cartilage *vs*. 2.8% in OA cartilage^24^), the development of novel, reliable and reproducible methodologies for their selective identification, isolation and characterisation is necessary. The heterogeneous nature of adult stem and progenitor cells demands the use of cell surface markers to isolate a relatively pure population; however, whilst the surfaceome of MSCs has been quite thoroughly analysed using a variety of techniques including high-throughput MS-based approaches^6,16,25^, no studies have been conducted to analyse the surfaceome of CPCs and there have been no reports comparing the surfaceome of MSCs and CPCs. Defining the composition of the pericellular interface of cartilage progenitor cells and compare it to bone marrow-derived MSCs to which they are closely related is essential to identify potential biomarkers and to understand their biology.

The major aim of this study therefore was to fine-tune an existing methodology that is considered to be highly selective for cell surface proteins^4,5^ and tailor it to reliably identify the surfaceome of migratory CPC with high selectivity and efficiency, and to compare the molecular composition of their surfaceome to that of bone marrow-derived MSCs.

In a previous study we employed a Triton X-114 based phase separation technique using nanoLC-MS/MS combined with shotgun proteomics to identify chondrocyte membrane proteins; of the 315 proteins, a fairly high proportion were membrane proteins (56%), but only 78 unique PM proteins (~25%) were reliably identified^26^. Weekes and colleagues have compared five methodologies for the enrichment of PM proteins and concluded that the isolation of sialylated glycoproteins with aminooxy-biotin was highly reproducible and identified PM proteins at high purity (68% of all annotated proteins)^5^. In another comparative study, Hörmann and colleagues concluded that whilst the sulfo-NHS-SS-biotinylation approach coupled to sodium dodecyl-sulphate SDS elution yielded the highest absolute number of PM proteins (49%; 650/1305), the “glycocapture” technique with aminooxy-biotin resulted in the highest relative amount of PM proteins compared to non-PM proteins (74%; 340/468), albeit with a somewhat lower absolute number of PM proteins^4^.

In the aminooxy-biotinylation approach used in this study, high-quality preparations enriched with cell surface and plasma membrane proteins were prepared first, which were then subjected to analysis by a mass spectrometer to detect low-abundance peptides. The enrichment step was essential, as cell surface biomarkers are often expressed at low copy numbers^6^. Also, because CPCs are sparse in articular cartilage and are therefore difficult to obtain in large amounts, techniques employed to discover biomarkers need to be highly selective and sensitive to the cell-surface proteome. Our methodology has two major advantages over antibody-based profiling approaches to surfaceome characterisation; firstly, it enables identification of the broad spectrum of plasma membrane glycoproteins, and secondly, the labelling is performed on live cells. A further advantage of the aminooxy-biotin strategy is that the covalent-bond based chemical enrichment is more selective than affinity-based interactions because of the strength from the chemical bonding^6^. This is reflected in the high numbers of cell surface proteins identified in this study; in our hands, the aminooxy-biotin based purification approach resulted in 1256 protein IDs, of which 791 proteins (63%) were annotated as surfaceome proteins. Other groups have identified similar membrane protein numbers using this technique and reported a similar marker profile when studying MSC populations^4,16,27^. However, this is the first study whereby the method has been applied to CPCs. It must be noted that some of the proteins labelled as nuclear or cytoplasmic may have resided additionally in the plasma membrane, but were not included in the present analysis.

The applicability of the approach used in this study is demonstrated by identifying cell surface CD markers presently used to define the cellular phenotype of MSCs. We reliably identified all surface markers except for Stro-1; we were unable to detect Stro-1 expression with this method as although Stro-1 has been considered the best-known MSC marker, and Stro-1 antibody has been widely used for the recognition and isolation of various types of MSC, its molecular identity remains completely unknown^28^. As expected, MSCs fulfilled the minimum criteria for mesenchymal stem cells; we also established that CPCs were highly similar to bone marrow-derived MSCs in terms of surface CD marker expression. Our findings are in complete agreement with the original description on this cell type; CPCs were positive for integrins beta-1, alpha-3, and alpha-5, as well as CD13, CD29, CD44, CD73, CD90 and CD105, and were negative for the markers CD34, CD31, CD117, as well as CD45. However, markers conventionally assigned to bone marrow-derived stromal cells including CD18 (integrin beta-2), CD31 and CD271 were negative in CPC populations^23^. Whilst present, not all surface CD markers exhibited the same level of expression; CD49b (integrin alpha-2) was enriched in CPC compared to MSC, whereas the majority of other markers showed lower quantitative MS scores in CPC. Given that one of the immunosuppressive mechanisms of MSCs involves adenosine production by ecto-5′-nucleotidase^29^, the similar relative quantitative data for CD73 in the two cell types suggests a similar immunosuppressive effect, a biological feature with potential utility for *in vivo* tissue engineering purposes. The relative low abundance of the vascular cell adhesion molecule VCAM1 (CD106) is in line with the observed migratory ability of CPCs as VCAM1 has been associated with the inhibition of migration^30^. Therefore, the low abundance of VCAM1 is a useful phenotypic feature. There are conflicting data with regards to endoglin (CD105) expression in MSCs; some studies suggest that the chondrogenic potential of MSCs is linked to CD105 expression^31^, but it has recently been proven that endoglin expression on culture-expanded BM-MSC populations is not associated with the chondroprogenitor phenotype^32^. Taken together, these results confirm the fact that although CPCs are closely related to BM-MSCs, they represent a distinct cell population with a characteristic surface CD marker expression profile.

Our biotinylation approach has contributed to the identification of several molecules that are significantly differentially expressed between the two cell populations, suggesting that their expression profiles are likely to be involved in establishing distinct MSC and chondroprogenitor phenotypes (see Fig. 4). Although it is well beyond the remit of this study to define the full significance of the expression of all identified CPC cell-surface markers, we have chosen KCNMA1, the alpha-1 subunit of the calcium-activated potassium channel KCa1.1 (also known as large conductance calcium-activated potassium channel, subfamily M, alpha member 1, or BK) to validate the efficacy of the glycocapture technique in predicting the differential expression pattern of proteins. We and others have reported that undifferentiated MSC, chondroprogenitor cells and mature chondrocytes express distinct patterns of ion channel mRNAs and functional ion channel proteins (referred to as the channelome^33^) that contribute to physiological cell functions^34–36^. Whole-cell voltage clamped BM-MSCs were showing abundant outward currents rapidly activated at potentials positive to +20 mV, identified as a large-conductance Ca^2+^-activated K^+^ current, conducted by BK channels, due to its high sensitivity to tetraethylammonium and its inhibition by 100 nM iberiotoxin^37,38^. Each BK channel consists of pore-forming alpha subunits arranged as tetramers that act in conjunction with tissue specific regulatory beta and/or gamma subunits^39^. BK channels are required for the proliferation, as well as adipogenic and osteogenic differentiation of MSCs, indicating their essential roles in maintaining the MSC phenotype and also in bone regeneration^40^. KCNMA1 was showing differences between normal and OA synovial fluid mesenchymal progenitor cells (sfMPCs) at the transcriptional and functional levels^41^, indicating that KCNMA1 is one component of the channelome in normal and OA progenitor cells that directly interacts with the ionic composition of the environment. BK channels have recently been shown to represent the predominant Ca^2+^-activated K^+^ channels in MSCs, which can serve as effectors downstream of G-protein-coupled receptor-mediated Ca^2+^ signalling and thus involved in almost every aspect of cellular physiology^42^.

In contrast, the role of ion channels, KCNMA1 in particular, in chondrogenic progenitor cells remains poorly understood. Given that articular cartilage-derived CPCs are believed to be further along in their commitment to the chondrogenic lineage compared to BM-MSCs^8^, it is logical to assume that the gene expression profiles of CPCs exhibit intermediate patterns between MSCs and chondrocytes. The BK channel is functional in chondrocytes and its roles range from being involved in mechano- and osmosensation to volume regulation and to histamine responsiveness^43^; moreover, KCNMA1 was found to be upregulated in patients with progressive OA^44^. The fact that KCNMA1 was enriched in CPCs compared to MSCs may indicate that ionic currents mediated through the BK channel play a role in CPC cellular physiology and that it may also be involved in the differentiation of cartilage progenitor cells. Other potassium channels including the voltage-gated K_V_1.1 have also been reported to control chondrogenesis through modulating the resting membrane potential (RMP) and cytosolic Ca^2+^ oscillations in micromass cultures. The detailed analysis of the contribution of ionic currents to CPC physiology and differentiation through the KCa1.1 K^+^ channel, however, is beyond the scope of the present study and shall be the focus of future research.

## Conclusions

Articular cartilage-derived CPCs may open new avenues for tissue engineering applications, and also for intrinsic cartilage repair processes, particularly during OA. To fully exploit the inherent chondrogenic capacity of CPCs, the biology of this cell type must be much better characterised. To this end, we employed an aminooxy-biotin based glycocapture approach combined with high throughput shotgun quantitative proteomics to define the surface proteome of CPCs and compared the cell-surface-enriched fractions to MSCs, and then a proteomic approach was used to evaluate protein changes between undifferentiated MSCs and CPCs. A high proportion of cell surface proteins were detected as 63% of the proteins identified were membrane or surface proteins. The proteins were annotated, and the surfaceome proteins were categorized. We confirmed that although closely related to BM-MSCs, CPCs are characterised by a distinct cellular interface. Our results provide the first repository for proteomic data on the surfaceome of two closely related cell types with potential in cartilage tissue engineering. However, our study has limitations, as the shotgun approach itself has drawbacks of missing data being a relatively common phenomenon, especially with such extremely low-abundance proteins as certain components of the surfaceome. Some of the proteins designated as unique in this data set might still be present in the other cell type, just not picked for tandem MS with the current settings and therefore should only be used with care.

Fruitful approaches to the systematic analysis of the surfaceome might include screening the list of candidates from this profile. This dataset is a significant step toward a deeper understanding of CPC biology and a higher resolution examination of the surfaceome in these cells. This strategy may also provide novel insights into the phenotypic transformation of the surfaceome in chondrocytes and synoviocytes during the development of OA and inflammatory synovitis.

## Materials and Methods

### Cell cultures

For migratory chondrogenic progenitor cell (CPC) experiments, human telomerase reverse transcriptase (hTERT)-immortalised CPCs derived from osteoarthritic knee articular cartilage were used as previously described^23,45^. Immortalised CPCs from a single clone derived from one patient (female, late-stage OA, 52 years of age; designated CPC241) were cultured in monolayers in 175 cm^2^ cell culture flasks (Nunc, Thermo Fisher Scientific, Waltham, MA, USA) until ~90% confluency in GlutaMAX DMEM (1.0 g/L glucose; Gibco, Thermo Fisher Scientific) containing 10% FBS (foetal bovine serum; Gibco) and 50 μg/mL gentamycin (Sigma-Aldrich, St. Louis, MO, USA).

**Figure 6:**
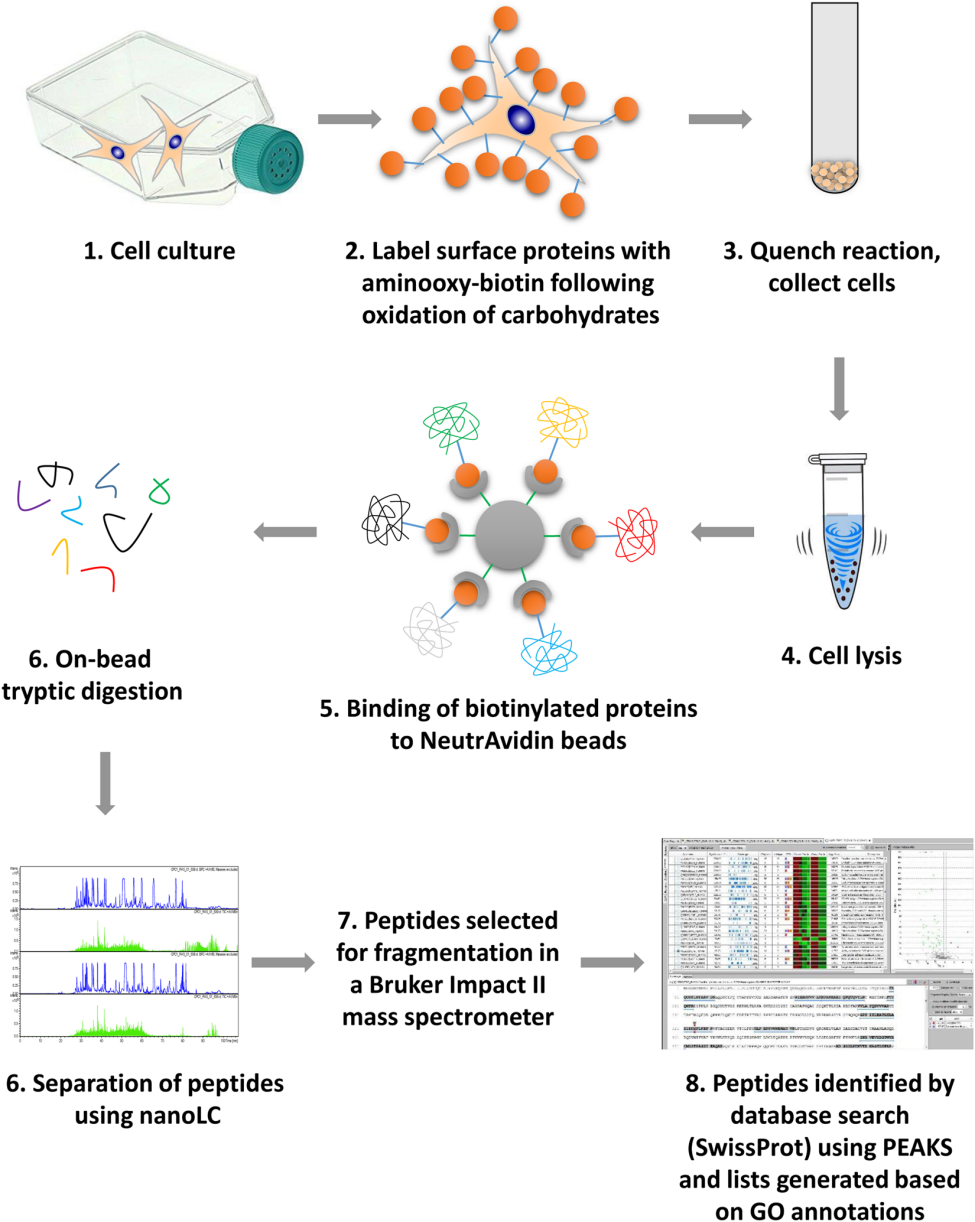
Schematic overview of the experimental design used in this study.

Bone marrow-derived human mesenchymal stem cells (MSCs) were purchased from Lonza (Basel, Switzerland). The cells were received at passage 2 and were expanded until passage 4 in Lonza hMSC medium at 37 °C in a humidified atmosphere of a CO_2_ incubator. MSCs were hTERT-immortalised as described by Okamoto *et al*.^46^ with the modifications detailed in Saeed *et al*.^47^. Immortalised MSCs were expanded in monolayers in 175 cm^2^ cell culture flasks (Nunc, Thermo Fisher Scientific) until ~90% confluency in GlutaMAX DMEM (4.5 g/L glucose; Gibco) containing 10% FCS (Gibco) and 1% Penicillin/Streptomycin solution (Sigma-Aldrich). A schematic overview of the experimental design is shown in Fig. 6.

### Sample preparation and enrichment of cell surface glycoproteins using aminooxy-biotin labelling

Cell surface sialylated membrane glycoproteins were enriched according to the protocols published elsewhere^4,17^, with minor modifications. The protein labelling process involved two steps; first, aldehyde groups were introduced into the glycoproteins by mild periodate oxidation, and then the functionalized proteins were incubated with the aminooxy-biotin reagent, covalently attaching biotin to the aldehyde group^17^. Approximately 90% confluent cultures of CPCs and MSCs were washed twice with phosphate buffered saline (PBS), then cells were incubated in 20 mL of serum-free DMEM for 1 h at 37 °C in a standard tissue culture incubator. Following removal of media, cells were washed thrice with 10 mL of ice-cold PBS per flask, surface sialic acid residues were oxidized and then biotinylated by adding 4 mL oxidation/biotinylation mix consisting of 1 mM sodium meta-periodate (Thermo Fisher Scientific), 100 mM aminooxy-biotin (Biotium Inc., Hayward, CA) and 10 mM aniline (Thermo Fisher Scientific) in ice-cold PBS (pH 6.7). Cells were incubated in this mixture with rocking in the dark at 4 °C for 1 hour. The oxidation mixture was quenched by the addition of glycerol to a final concentration of 1 mM. Cells were washed twice in PBS containing 1 mM CaCl_2_ and 0.5 mM MgCl_2_, then gently scraped off the surface of flasks using a cell scraper and collected by centrifugation (1000 × *g* for 5 min). The resulting cell pellets were resuspended in lysis buffer [containing 1% Triton X-100 (Sigma-Aldrich), 150 mM NaCl (Sigma-Aldrich), 1× protease inhibitor (Sigma-Aldrich), 5 mM iodoacetamide (IAA; Thermo Fisher Scientific), 0.1 mg/mL PMSF and 10 mM Tris-HCl; pH 7.6 (Sigma-Aldrich)], and incubated at 4 °C for 30 min with vortexing the mixture every 5 min.

Cell debris and nuclei were removed by centrifugation at 4 °C at 2,800 × *g* for 10 min and then at 16,000 × *g* for 2 × 15 min. To isolate labelled glycoproteins, 250 μL aliquots of Pierce NeutrAvidin agarose beads (Life Technologies, Eugene, OR, USA) were added to Snap Cap spin columns and incubated with the cell lysates for 2 h at 4 °C with continuous rocking. To eliminate non-specifically bound proteins, multiple washing steps were performed (20 × 600 μL followed by centrifugation at 1000 × *g* for 1 min at each step). Beads were initially washed 20× with lysis buffer, 20× with PBS/0.5% (w/v) SDS and incubated for 20 min at room temperature with PBS/0.5% (w/v) SDS/100 mM dithiothreitol (DTT), then centrifuged. Further washing was performed 20× with UC buffer (6 M urea, 100 mM Tris-HCl pH 8.5), followed by alkylation for 20 min at room temperature with UC buffer containing 50 mM IAA. Beads were then washed again (20× per step with centrifugation after each step) using UC buffer, 5 M NaCl, 100 mM Na_2_CO_3_, PBS and then ultrapure water, resuspended in 400 μL of 50 mM ammonium bicarbonate (AMBIC) containing 5 μg Trypsin Gold (Promega, Madison, WI, USA), then transferred to a microcentrifuge tube and biotinylated glycoproteins were digested on-beads overnight at 37 °C. Then, beads were transferred to new Snap Cap spin columns and the tryptic peptides were collected via centrifugation at 1000 × *g* for 1 min. Finally, the beads were rinsed with 200 μL of 50 mM AMBIC and tryptic fractions were pooled. The tryptic peptide mixtures were centrifuged at 16,000 × *g* for 30 sec and solvents were evaporated in a centrifugal vacuum concentrator (Eppendorf, Hamburg, Germany) for approximately 2 h.

### LC-MS/MS analysis

The tryptic peptides generated using the aminooxy-biotinylation protocol were injected into a 25 cm × 75 μm C_18_ Pepmap column using a Dionex UltiMate 3000 RSLCnano chromatography platform with a flow rate of 300 nL/min to separate peptides at 50 °C column temperature. 3 μL of each sample was injected into the HPLC column. After peptide binding and washing processes on the column, the complex peptide mixture was separated and eluted by a gradient of solution A (100% water +0.1% formic acid) and solution B (100% ACN + 0.1% formic acid) over 60 min, followed by column washing and re-equilibration. The gradient initially started at 2%–40% solvent B followed by a ramp to 95% solvent B and a hold at high solvent. The peptides were delivered to a Bruker Impact II instrument with nanoBooster CaptiveSpray source. MS acquisition rate was 5 Hz, followed by 10–20 Hz MS2 acquisition rate, depending on precursor intensity. The top 20 most intense ions from each MS scan were selected for fragmentation. The nanoLC-MS/MS analysis was performed once on every biological sample (3 biological replicates for each cell type).

### Peptide and protein identification, and quantitative analysis of LC-MS/MS results

Processed data from the analysed samples were compiled into MGF files, which were converted to the mzML format using MSConvert (ProteoWizard 3.0.18212), and then filtered, *de novo* sequenced and assigned with protein ID using PEAKS Studio 8.5 software^48^ (Bioinformatics Solutions, Waterloo, Canada), by searching against the human SwissProt database (March 2018; 20,314 entries). Label-free quantitation (LFQ) analysis was performed using the quantitation module in PEAKS Studio with the following parameters: trypsin restrictions for enzymes and one allowed missed cleavage at one peptide end. Normalization during quantitation was carried out in PEAKS Studio 8.5 based on Total Ion Chromatogram (TIC). The parent mass tolerance was set to 30 ppm using monoisotopic mass, and a fragment ion mass tolerance was set to 0.1 Da. Carbamidomethyl cysteine (+57.0215 on C) was specified in PEAKS as a fixed modification. Methionine oxidation (+15.99 on M), deamidation of asparagine and glutamine (NQ + 0.98) were set as variable modifications. Data were validated using the FDR method built in PEAKS 8.5 and protein identifications were accepted with a confidence score (−10lgP) >20 for peptides and (−10lgP) >15 for proteins; a minimum of 1 peptide per protein after data were filtered for 1% FDR for proteins. The raw datasets generated during and/or analysed during the current study are available in the figshare repository at the following link: 10.6084/m9.figshare.6154421^49^.

### Data analysis and bioinformatics

After mass spectrometric identification using PEAKS Studio, 1256 proteins were classified using the UniProt (http://www.uniprot.org/) database (release 2018_11), taking into account homologous proteins and further literature information. Given the discovery-based shotgun approach applied in this study, as well as the very low abundance of certain surface proteins, we included every protein into the lists that have been reliably identified in at least one biological replicate; if a protein was present above the limit of detection in a sample, as trypsin has been used for on-bead digestion, the probability of at least one peptide being selected for fragmentation is high. For many proteins, assigning a definitive cellular compartment and/or biological function was a difficult task because of the limitations in accurate predictions and lack of experimental evidence. Also, many proteins may reside in multiple cellular compartments. To assign identified proteins to specific organelles or to the plasma membrane, the references to subcellular localisations in the UniProt database, as well as gene ontology (GO) annotations were used.

GO terms, describing molecular function and subcellular localisation, downloaded from UniProt were used to provide an initial view of the function and family affiliation of each identified protein. This information was used to divide them into three functional classes (receptors, enzymes, and transporters), one extracellular matrix component class, and one structural/adhesion protein class, based on already published classification criteria^21^. Proteins that did not fit into any of the above functional classes were marked as unclassified. Given that the GO annotations for certain proteins contained multiple entries, we were unable to assign a single molecular function. To this end, some proteins are included in more than one list. For example, NMDE3, the glutamate receptor ionotropic NMDA 2C entry is listed under both transporters and receptors as NMDARs are ligand-gated ion channels.

To determine the protein expression patterns between the two cell types, fold change values were determined by calculating the ratios for CPC versus MSC (control) using all data from the PEAKS Studio protein identification export. For significantly differentially expressed proteins, the analysis utilised the top-3 peptides from each protein. We used the cut-off threshold of ±1.5 FC for each protein as being up/down regulated in CPC *vs*. MSC, and considered proteins with FC values below the ±1.5 threshold as unchanged.

### Validation of selected proteins by western blotting

Cells were grown in T75 tissue culture flasks until ~90% confluency under standard cell culture conditions as per described above, and then scraped off the surface of flasks using a rubber cell scraper. Cell pellets were resuspended in 100 μL of RIPA lysis and extraction buffer (Thermo Fisher Scientific) and lysed using a sonicator probe. Protein concentration in each sample was adjusted by adding the required amount of 2× Laemmli sample buffer (Bio-Rad, Hercules, CA, USA) and boiled at 95 °C for 5 min. Protein samples containing 20 μg of total protein per lane were loaded onto 10% sodium dodecyl sulphate polyacrylamide gel electrophoresis (SDS–PAGE) gels and separated under reducing conditions using a Bio-Rad Mini-PROTEAN Tetra Cell for immunological detection of proteins. Proteins were transferred onto 0.2 µm nitrocellulose membranes using the Trans-Blot Turbo RTA Mini Nitrocellulose Transfer Kit (Bio-Rad) and a Trans-Blot Turbo rapid western blotting transfer system (Bio-Rad). Transfer time was 8 min at 2.5 A. After blocking in Odyssey Blocking Buffer in PBS (Li-Cor, Lincoln, NE, USA) for 1 hour at room temperature, membranes were incubated with the rabbit anti-KCNMA1 primary polyclonal antibody raised against the C-terminal fragment of the alpha-1 subunit of the large conductance calcium-activated potassium channel (Aviva Systems Biology, San Diego, Ca, USA; cat. number ARP35092_P050; diluted in 1:500) in the blocking buffer at 4 °C overnight, with gentle rotation. After extensive wash steps with PBS containing 0.1% Tween-20 (PBST) membranes were incubated with 0.2 µg/mL IRDye 800CW goat anti-rabbit IgG secondary antibody (Li-Cor; used in 1:5000 dilution) in the blocking buffer at room temperature for one hour. After washing four times in PBST, membranes were transferred to an Odyssey FC imaging system (Li-Cor) and bands were detected using the 800 nm channel.

An antibody raised against the internal house-keeping control protein β-actin (β-actin rabbit monoclonal antibody; Li-Cor; cat. number 926-42210; diluted in 1:1000) was used as a normalisation antibody. Membranes that have been imaged for KCNMA1 were incubated in the blocking buffer for an additional 2 hours at room temperature, and then exposed to the anti β-actin antibody overnight. Following washing three times with PBST, membranes were incubated with 0.2 µg/mL IRDye 680CW goat anti-rabbit IgG secondary antibody (Li-Cor; used in 1:5000 dilution) at room temperature for one hour. After washing four times in PBST, membranes were transferred to an Odyssey FC imaging system (Li-Cor) and bands were detected using the 700 nm channel. Optical density values of detected bands were calculated using the Image Studio software and the values for KCNMA1 were normalised to those of β-actin. Tests for statistical significance were performed using paired Student’s T test. Effect size was calculated using Cohen’s d test (with effect sizes classified as small (d  ≥ 0.2), medium (d  ≥  0.5), large (d ≥ 0.8) and very large (d ≥ 1.3)^50^.

### KCNMA1 immunocytochemistry

Cells were cultured on glass coverslips placed into 24-well tissue culture plates until ~75% confluency. After rinsing with PBS, cultures were fixed in Sainte-Marie’s fixative (3% anhydrous acetic acid in absolute ethanol) for 15 min, and then washed with a descending series of ethanol. After rinsing in 0.2% PBST, nonspecific binding sites were blocked with 1% BSA-PBST at 37 °C for 30 min. Cultures were then incubated with the rabbit anti-KCNMA1 primary polyclonal antibody (Aviva Systems Biology, diluted in 1:250) in the blocking buffer at 4 °C overnight. For visualisation of the primary antibodies, anti-rabbit Alexa488 conjugated secondary antibody (Life Technologies Corporation, Carlsbad, CA, USA) was used at a dilution of 1:1000. The specificity of the reaction was confirmed by incubating the cultures with the secondary antibody only; in these experiments, no signals were detected (see Supplementary Fig. S1). Cultures were mounted in Vectashield mounting medium (Vector Laboratories, Peterborough, England) containing DAPI for nuclear DNA staining. Single 1 μm-thick optical sections were scanned with an Olympus FV1000 confocal microscope. Scanning was carried out with a ×60 oil-immersion lens (NA = 1.42).

## Supplementary information

Supplementary materials

Significantly differentially expressed proteins
