# Supplementary material for: Molecular phenotyping of the surfaceome of migratory chondroprogenitors and mesenchymal stem cells using biotinylation, glycocapture and quantitative LC-MS/MS proteomic analysis
Source: Sci Rep. 2019 Jun 21;9:9018. doi: 10.1038/s41598-019-44957-y (PMC6588563; doi:10.1038/s41598-019-44957-y)
Supplement: Supplementary file 1 — Supplementary materials [file 41598_2019_44957_MOESM1_ESM.pdf]

Supplementary Materials for

Molecular phenotyping of the surfaceome of migratory chondroprogenitors and mesenchymal stem cells using biotinylation, glycocapture and quantitative LC-MS/MS proteomic analysis

Csaba Matta*, David J. Boocock, Christopher R. Fellows, Nicolai Miosge, James E. Dixon, Susan Liddell, Julia Smith & Ali Mobasheri*

*Corresponding authors. E-mail: matta.csaba@med.unideb.hu (C.M.), ali.mobasheri.manuscripts@gmail.com (A.M.)

This PDF file includes:

Fig. S1. Negative control for KCNMA1 IHC.
Fig. S2. Uncropped membrane images for KCNMA1 immunoblots.
Table S1. List of proteins classified as Transporters.
Table S2. List of proteins classified as Receptors.
Table S3. List of proteins classified as Enzymes.
Table S4. List of proteins classified as Extracellular matrix components.
Table S5. List of proteins classified as Adhesion/junction/cytoskeletal proteins.
Table S6. List of proteins that could not be classified into any of the previous groups (unclassified).
Table S7. List of proteins exported from the PEAKS Studio quantitation module, showing significantly differentially expressed proteins following quantitative LC-MS/MS analysis (cut-off fold change >1.5) utilising the Top 3 peptides from each protein

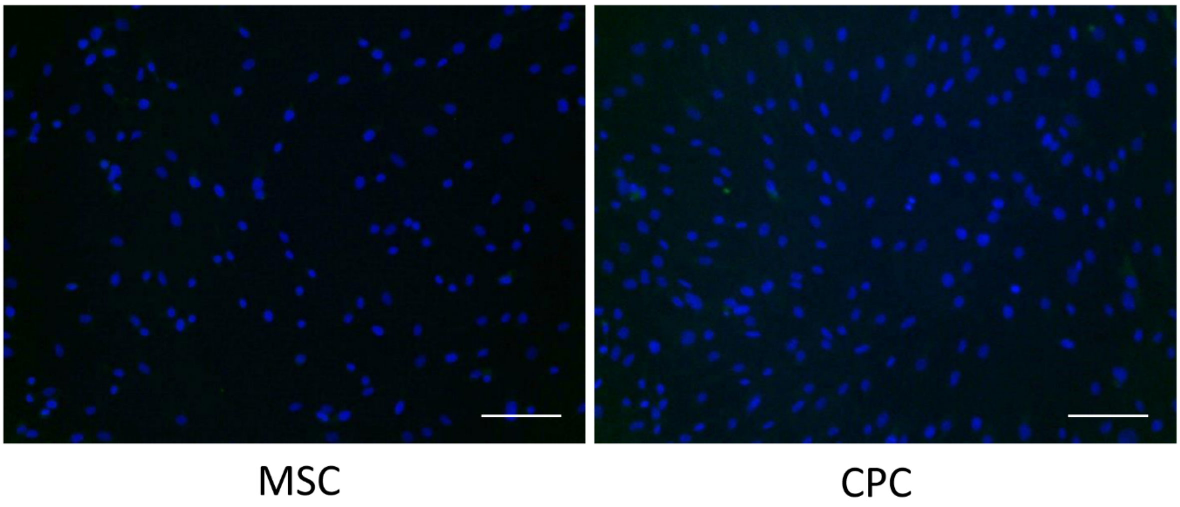

Fig. S1. Negative control experiments for the immunocytochemical detection of intracellular distribution of KCNMA1 in MSCs and CPCs. Cells were incubated with the Alexa488-conjugated anti-rabbit secondary antibodies only (no primary antibodies were added). Nuclear DNA was stained with DAPI. Data shown are representative out of 3 independent experiments. Scale bar, 100 µm.

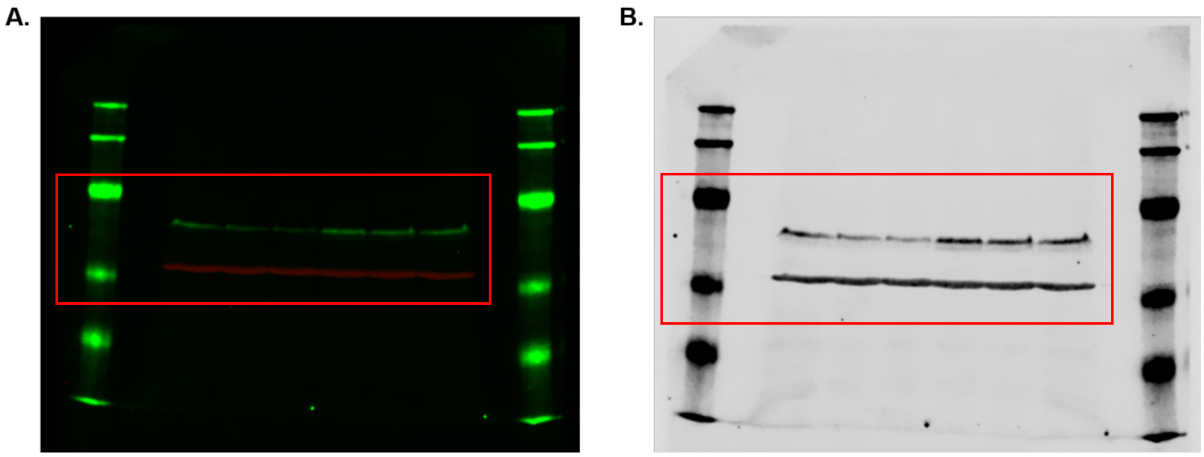

Fig. S2. Uncropped membrane images for KCNMA1 immunoblot experiments. A. KCNMA1 (green) and β-actin (red) bands visualised using the Odyssey® FC imaging system. Bands were detected using the 800 nm (for KCNMA1) and 700 nm (for β-actin) channels. B. Image converted to grayscale and then bands for KCNMA1 were cropped as indicated by the red frame and shown in the manuscript in **Figure 5.**

The following Tables (**Tables S1–S6**) were generated using all data from the PEAKS Studio protein identification export. The table of significantly differentially expressed proteins (**Table S7**) utilises the Top 3 peptides from each protein.

Table S1. List of proteins classified as *Transporters* based on GO annotations. Relative expression levels in CPC vs MSC are based on normalised quantitative MS data.

Accession	Protein ID	Description	-10lgP	Coverage (%) CPC	Coverage (%) MSC	Area CPC	Area MSC	Fold change	Expression
P62258	1433E_HUMAN	14-3-3 protein epsilon	106.17	13	12	0	109 000		MSC only
P27348	1433T_HUMAN	14-3-3 protein theta	145.99	36	36	38 000	162 000	-4.26	Down in CPC
P63104	1433Z_HUMAN	14-3-3 protein zeta/delta	120.08	26	32	254 000	95 100	2.67	Up in CPC
P08195	4F2_HUMAN	4F2 cell-surface antigen heavy chain	372.23	51	62	22 800 000	89 500 000	-3.93	Down in CPC
Q15758	AAAT_HUMAN	Neutral amino acid transporter B(0)	283.74	32	43	8 190 000	15 200 000	-1.86	Down in CPC
P78363	ABCA4_HUMAN	Retinal-specific ATP-binding cassette transporter	64.81	1	1	390 000			CPC only
Q86UK0	ABCAC_HUMAN	ATP-binding cassette sub-family A member 12	62.3	1	1	359	3 420	-9.53	Down in CPC
O95342	ABCBB_HUMAN	Bile salt export pump	63.52	2	2	0			N/A
O60706	ABCC9_HUMAN	ATP-binding cassette sub-family C member 9	52.72	1	1	0			N/A
Q96J66	ABCCB_HUMAN	ATP-binding cassette sub-family C member 11	82.7	11	7	377 000	138 000	2.73	Up in CPC
P12814	ACTN1_HUMAN	Alpha-actinin-1	83.85	0	14		79 800		MSC only
O43707	ACTN4_HUMAN	Alpha-actinin-4	78.19	2	9	10 700	23 900	-2.23	Down in CPC
P07550	ADRB2_HUMAN	Beta-2 adrenergic receptor	47	1	5		34 000		MSC only
P05141	ADT2_HUMAN	ADP/ATP translocase 2	142.5	18	29	246 000	410 000	-1.67	Down in CPC
P16157	ANK1_HUMAN	Ankyrin-1	61.03	2	1	0			N/A
Q01484	ANK2_HUMAN	Ankyrin-2	82.07	2	1	0			N/A
Q12955	ANK3_HUMAN	Ankyrin-3	83.96	1	1	54 700			CPC only
Q4KMQ2	ANO6_HUMAN	Anoctamin-6	190.41	12	22	799 000	588 000	1.36	No change
P07355	ANXA2_HUMAN	Annexin A2	271.24	52	49	4 650 000	7 470 000	-1.61	Down in CPC
P08133	ANXA6_HUMAN	Annexin A6	47.1	0	3		23 700		MSC only
P04114	APOB_HUMAN	Apolipoprotein B-100	76.18	1	1	13 100	209 000	-15.95	Down in CPC
P02649	APOE_HUMAN	Apolipoprotein E	77.45	15	17	38 200	120 000	-3.14	Down in CPC
Q12797	ASPH_HUMAN	Aspartyl/asparaginyl beta-hydroxylase	52.66	1	3		49 000		MSC only
P54707	AT12A_HUMAN	Potassium-transporting ATPase alpha chain 2	106.07	7	8	55 500	224 000	-4.04	Down in CPC
P05023	AT1A1_HUMAN	Sodium/potassium-transporting ATPase subunit alpha-1	277.57	32	31	974 000	1 700 000	-1.75	Down in CPC
P50993	AT1A2_HUMAN	Sodium/potassium-transporting ATPase subunit alpha-2	207.79	16	16	0	278 000		MSC only
P13637	AT1A3_HUMAN	Sodium/potassium-transporting ATPase subunit alpha-3	229.04	19	19	0	71 200		MSC only
Q13733	AT1A4_HUMAN	Sodium/potassium-transporting ATPase subunit alpha-4	166.46	15	14	239 000 000	211 000 000	1.13	No change
P05026	AT1B1_HUMAN	Sodium/potassium-transporting ATPase subunit beta-1	149.44	24	32	417 000	941 000	-2.26	Down in CPC
P54709	AT1B3_HUMAN	Sodium/potassium-transporting ATPase subunit beta-3	223.69	40	57	3 010 000	9 740 000	-3.24	Down in CPC
P20020	AT2B1_HUMAN	Plasma membrane calcium-transporting ATPase 1	236.08	16	22	472 000	664 000	-1.41	No change
Q16720	AT2B3_HUMAN	Plasma membrane calcium-transporting ATPase 3	200.04	8	10	0	11 200		MSC only
P20648	ATP4A_HUMAN	Potassium-transporting ATPase alpha chain 1	118.32	10	10	98 400	3 400 000	-34.55	Down in CPC
P04920	B3A2_HUMAN	Anion exchange protein 2	258.05	27	24	1 320 000	1 100 000	1.20	No change
P48751	B3A3_HUMAN	Anion exchange protein 3	86.91	2	5	21 800	45 500	-2.09	Down in CPC
P35613	BASI_HUMAN	Basigin	277.27	37	42	12 900 000	22 000 000	-1.71	Down in CPC
P54289	CA2D1_HUMAN	Voltage-dependent calcium channel subunit alpha-2/delta-1	261.04	26	28	1 160 000	1 360 000	-1.17	No change
Q9NY47	CA2D2_HUMAN	Voltage-dependent calcium channel subunit alpha-2/delta-2	83.81	3	2	0			N/A

Table S1. List of proteins classified as *Transporters* based on GO annotations *(continued)*

Accession	Protein ID	Description	-10lgP	Coverage (%) CPC	Coverage (%) MSC	Area CPC	Area MSC	Fold change	Expression
Q7Z3S7	CA2D4_HUMAN	Voltage-dependent calcium channel subunit alpha-2/delta-4	52.74	2	3	155 000	282 000	-1.82	Down in CPC
O00555	CAC1A_HUMAN	Voltage-dependent P/Q-type calcium channel subunit alpha-1A	57.13	0	2		95 200		MSC only
Q13936	CAC1C_HUMAN	Voltage-dependent L-type calcium channel subunit alpha-1C	58.24	1	2	0	0		N/A
Q15878	CAC1E_HUMAN	Voltage-dependent R-type calcium channel subunit alpha-1E	42.96	1	0	0			N/A
O95180	CAC1H_HUMAN	Voltage-dependent T-type calcium channel subunit alpha-1H	58.35	1	0	17 100			CPC only
Q9NV96	CC50A_HUMAN	Cell cycle control protein 50A	157.4	19	21	265 000	362 000	-1.37	Down in CPC
O00299	CLIC1_HUMAN	Chloride intracellular channel protein 1	75.11	10	27	8 170	178 000	-21.79	Down in CPC
P29973	CNGA1_HUMAN	cGMP-gated cation channel alpha-1	32.92	3	1	2 340			CPC only
O15431	COPT1_HUMAN	High affinity copper uptake protein 1	77.42	10	10	52 300	142 000	-2.72	Down in CPC
Q96D31	CRCM1_HUMAN	Calcium release-activated calcium channel protein 1	91.24	13	18	21 200	141 000	-6.65	Down in CPC
Q8IWA5	CTL2_HUMAN	Choline transporter-like protein 2	255.8	14	24	370 000	5 510 000	-14.89	Down in CPC
P35222	CTNB1_HUMAN	Catenin beta-1	57.18	4	3	0			N/A
P30825	CTR1_HUMAN	High affinity cationic amino acid transporter 1	142.2	8	16	8 700 000	1 030 000	8.45	Up in CPC
P52569	CTR2_HUMAN	Cationic amino acid transporter 2	90.06	4	3	0			N/A
O60494	CUBN_HUMAN	Cubilin	48.45	0	1		28 100		MSC only
P43003	EAA1_HUMAN	Excitatory amino acid transporter 1	113.78	0	15		194 000		MSC only
P43005	EAA3_HUMAN	Excitatory amino acid transporter 3	146.61	14	19	1 190 000	236 000	5.04	Up in CPC
Q13642	FHL1_HUMAN	Four and a half LIM domains protein 1	44.08	0	7		139 000		MSC only
P21333	FLNA_HUMAN	Filamin-A	359.24	30	37	4 550 000	7 200 000	-1.58	Down in CPC
P11166	GTR1_HUMAN	Solute carrier family 2 facilitated glucose transporter member 1	153.25	12	14	638 000	809 000	-1.27	No change
P11169	GTR3_HUMAN	Solute carrier family 2 facilitated glucose transporter member 3	97.9	12	7	127 000	109 000	1.17	No change
P08238	HS90B_HUMAN	Heat shock protein HSP 90-beta	209.01	29	30	268 000	446 000	-1.66	Down in CPC
Q14974	IMB1_HUMAN	Importin subunit beta-1	63	3	7	29 500	166 000	-5.63	Down in CPC
P06756	ITAV_HUMAN	Integrin alpha-V	358.52	58	62	12 500 000	22 600 000	-1.81	Down in CPC
Q12791	KCMA1_HUMAN	Calcium-activated potassium channel subunit alpha-1	121.28	7	3	56 300	16 500	3.41	Up in CPC
P63252	KCNJ2_HUMAN	Inward rectifier potassium channel 2	34.42	2	1	8 660			CPC only
O43526	KCNQ2_HUMAN	Potassium voltage-gated channel subfamily KQT member 2	39.02	1	2	262 000			CPC only
Q01650	LAT1_HUMAN	Large neutral amino acids transporter small subunit 1	199.35	11	22	855 000	3 990 000	-4.67	Down in CPC
Q8IWT6	LRC8A_HUMAN	Volume-regulated anion channel subunit LRRC8A	154.95	11	15	248 000	446 000	-1.80	Down in CPC
Q8TDW0	LRC8C_HUMAN	Volume-regulated anion channel subunit LRRC8C	121.19	10	10	275 000	89 700	3.07	Up in CPC
Q7L1W4	LRC8D_HUMAN	Volume-regulated anion channel subunit LRRC8D	75.18	3	4	16 900	17 200	-1.02	No change
Q07954	LRP1_HUMAN	Prolow-density lipoprotein receptor-related protein 1	435.24	31	27	20 400 000	19 200 000	1.06	No change
P98164	LRP2_HUMAN	Low-density lipoprotein receptor-related protein 2	55.07	1	0	0			N/A
Q5S007	LRRK2_HUMAN	Leucine-rich repeat serine/threonine-protein kinase 2	58.27	1	1	98 800			CPC only
P53985	MOT1_HUMAN	Monocarboxylate transporter 1	96.56	4	4	59 900	220 000	-3.67	Down in CPC
O15427	MOT4_HUMAN	Monocarboxylate transporter 4	68.6	0	3		11 100		MSC only
Q00325	MPCP_HUMAN	Phosphate carrier protein mitochondrial	98.25	9	11	84 100	140 000	-1.66	Down in CPC
P20645	MPRD_HUMAN	Cation-dependent mannose-6-phosphate receptor	101.86	12	19	155 000	401 000	-2.59	Down in CPC
P33527	MRP1_HUMAN	Multidrug resistance-associated protein 1	357.42	44	44	11 400 000	6 040 000	1.89	Up in CPC
Q92887	MRP2_HUMAN	Canalicular multispecific organic anion transporter 1	94.25	8	5	25 300	319 000	-12.61	Down in CPC

Table S1. List of proteins classified as *Transporters* based on GO annotations *(continued)*

Accession	Protein ID	Description	-10lgP	Coverage (%) CPC	Coverage (%) MSC	Area CPC	Area MSC	Fold change	Expression
O15438	MRP3_HUMAN	Canalicular multispecific organic anion transporter 2	209.14	14	14	311 000	1 140 000	-3.67	Down in CPC
O15439	MRP4_HUMAN	Multidrug resistance-associated protein 4	237.13	16	23	2 150 000	6 640 000	-3.09	Down in CPC
Q5T3U5	MRP7_HUMAN	Multidrug resistance-associated protein 7	38.04	1	0	6 470			CPC only
P32418	NAC1_HUMAN	Sodium/calcium exchanger 1	87.7	4	5	19 800	82 900	-4.19	Down in CPC
Q96PU5	NED4L_HUMAN	E3 ubiquitin-protein ligase NEDD4-like	26.4	1	2		0		N/A
Q14957	NMDE3_HUMAN	Glutamate receptor ionotropic NMDA 2C	35.55	1	1	0			N/A
Q9Y639	NPTN_HUMAN	Neuroplastin	208.4	27	37	6 630 000	7 670 000	-1.16	No change
Q96RD7	PANX1_HUMAN	Pannexin-1	75.23	2	8	25 200	110 000	-4.37	Down in CPC
Q92508	PIEZ1_HUMAN	Piezo-type mechanosensitive ion channel component 1	279.67	16	18	1 650 000	2 920 000	-1.77	Down in CPC
P98161	PKD1_HUMAN	Polycystin-1	85.94	1	2	37 600	0		CPC only
P62937	PPIA_HUMAN	Peptidyl-prolyl cis-trans isomerase A	107.15	18	30	244 000	673 000	-2.76	Down in CPC
P04156	PRIO_HUMAN	Major prion protein	144.6	20	22	1 480 000	955 000	1.55	Up in CPC
P20338	RAB4A_HUMAN	Ras-related protein Rab-4A	62.69	9	7	0			N/A
P62834	RAP1A_HUMAN	Ras-related protein Rap-1A	46.2	7	14	13 400	5 820	2.30	Up in CPC
Q86UR5	RIMS1_HUMAN	Regulating synaptic membrane exocytosis protein 1	44.31	1	1	12 600			CPC only
Q9UQ26	RIMS2_HUMAN	Regulating synaptic membrane exocytosis protein 2	43.93	3	0	440 000			CPC only
P21817	RYR1_HUMAN	Ryanodine receptor 1	86.43	1	2	67 500	97 800	-1.45	No change
Q92736	RYR2_HUMAN	Ryanodine receptor 2	89.7	1	1	85 900			CPC only
P60903	S10AA_HUMAN	Protein S100-A10	100.24	41	35	431 000	669 000	-1.55	Down in CPC
Q13621	S12A1_HUMAN	Solute carrier family 12 member 1	96.75	4	2	35 700			CPC only
P55011	S12A2_HUMAN	Solute carrier family 12 member 2	264.7	26	35	2 010 000	3 390 000	-1.69	Down in CPC
Q9UP95	S12A4_HUMAN	Solute carrier family 12 member 4	199.94	17	24	245 000	813 000	-3.32	Down in CPC
Q9UHW9	S12A6_HUMAN	Solute carrier family 12 member 6	102.5	6	4	4 900			CPC only
Q9Y666	S12A7_HUMAN	Solute carrier family 12 member 7	155.98	11	22	236 000	1 090 000	-4.62	Down in CPC
Q8WUM9	S20A1_HUMAN	Sodium-dependent phosphate transporter 1	52.67	0	2		46 400		MSC only
Q9UGH3	S23A2_HUMAN	Solute carrier family 23 member 2	67.28	7	3	39 500	28 400	1.39	No change
P50443	S26A2_HUMAN	Sulfate transporter	59.8	4	3	9 000	45 500	-5.06	Down in CPC
Q99808	S29A1_HUMAN	Equilibrative nucleoside transporter 1	164.03	17	16	234 000	448 000	-1.91	Down in CPC
Q96QD8	S38A2_HUMAN	Sodium-coupled neutral amino acid transporter 2	188.32	12	20	367 000	2 400 000	-6.54	Down in CPC
Q8WUX1	S38A5_HUMAN	Sodium-coupled neutral amino acid transporter 5	85.45	8	1	122 000			CPC only
Q15043	S39AE_HUMAN	Zinc transporter ZIP14	177.53	15	20	1 690 000	1 560 000	1.08	No change
Q6U841	S4A10_HUMAN	Sodium-driven chloride bicarbonate exchanger	113.28	9	6	39 600	8 610	4.60	Up in CPC
Q9Y6R1	S4A4_HUMAN	Electrogenic sodium bicarbonate cotransporter 1	177.9	10	18	115 000	590 000	-5.13	Down in CPC
Q9Y6M7	S4A7_HUMAN	Sodium bicarbonate cotransporter 3	236.1	26	23	1 170 000	1 460 000	-1.25	No change
Q2Y0W8	S4A8_HUMAN	Electroneutral sodium bicarbonate exchanger 1	108.23	6	5	478 000	93 900	5.09	Up in CPC
P43007	SATT_HUMAN	Neutral amino acid transporter A	261.36	30	31	1 400 000	2 530 000	-1.81	Down in CPC
P53794	SC5A3_HUMAN	Sodium/myo-inositol cotransporter	131.66	14	14	199 000	1 500 000	-7.54	Down in CPC
Q9Y289	SC5A6_HUMAN	Sodium-dependent multivitamin transporter	128.81	9	9	189 000	264 000	-1.40	No change
P31641	SC6A6_HUMAN	Sodium- and chloride-dependent taurine transporter	108.48	9	9	232 000	295 000	-1.27	No change
P48067	SC6A9_HUMAN	Sodium- and chloride-dependent glycine transporter 1	83.89	3	4	142 000	76 400	1.86	Up in CPC

Table S1. List of proteins classified as *Transporters* based on GO annotations *(continued)*

Accession	Protein ID	Description	-10lgP	Coverage (%) CPC	Coverage (%) MSC	Area CPC	Area MSC	Fold change	Expression
Q9UQD0	SCN8A_HUMAN	Sodium channel protein type 8 subunit alpha	25.32	1	0	9 550			CPC only
P37088	SCNNA_HUMAN	Amiloride-sensitive sodium channel subunit alpha	43.48	1	1	0			N/A
P19634	SL9A1_HUMAN	Sodium/hydrogen exchanger 1	156.95	19	14	683 000	502 000	1.36	No change
Q92581	SL9A6_HUMAN	Sodium/hydrogen exchanger 6	83.6	3	7	9 860	156 000	-15.82	Down in CPC
P02786	TFR1_HUMAN	Transferrin receptor protein 1	308.46	50	55	7 140 000	13 300 000	-1.86	Down in CPC
O94759	TRPM2_HUMAN	Transient receptor potential cation channel subfamily M member 2	58.65	1	1	7 010			CPC only
Q8TD43	TRPM4_HUMAN	Transient receptor potential cation channel subfamily M member 4	47.94	0	2		4 940		MSC only
P21796	VDAC1_HUMAN	Voltage-dependent anion-selective channel protein 1	29.83	2	3		2 610 000		MSC only
P45880	VDAC2_HUMAN	Voltage-dependent anion-selective channel protein 2	156.72	24	38	341 000	815 000	-2.39	Down in CPC
Q9Y277	VDAC3_HUMAN	Voltage-dependent anion-selective channel protein 3	63.98	4	5		42 300		MSC only
Q9Y3S1	WNK2_HUMAN	Serine/threonine-protein kinase WNK2	30.74	0	1		0		N/A
P55060	XPO2_HUMAN	Exportin-2	72.7	1	3		39 000		MSC only
Q9Y6M5	ZNT1_HUMAN	Zinc transporter 1	165.06	25	24	410 000	975 000	-2.38	Down in CPC

Table S2. List of proteins classified as *Receptors* based on GO annotations. Relative expression levels in CPC vs MSC are based on normalised quantitative MS data.

Accession	Protein ID	Description	-10lgP	Coverage (%) CPC	Coverage (%) MSC	Area CPC	Area MSC	Fold change	Expression
P01891	1A68_HUMAN	HLA class I histocompatibility antigen A-68 alpha chain	277.41	33	48		1 610 000		MSC only
Q15758	AAAT_HUMAN	Neutral amino acid transporter B(0)	283.74	32	43	8 190 000	15 200 000	-1.86	Down in CPC
Q86UK0	ABCAC_HUMAN	ATP-binding cassette sub-family A member 12	62.3	1	1	359	3 420	-9.53	Down in CPC
O60706	ABCC9_HUMAN	ATP-binding cassette sub-family C member 9	52.72	1	1	0			N/A
P12821	ACE_HUMAN	Angiotensin-converting enzyme	146.61	4	15	442 000	1 450 000	-3.28	Down in CPC
Q07912	ACK1_HUMAN	Activated CDC42 kinase 1	51.4	3	1	29 200			CPC only
P12814	ACTN1_HUMAN	Alpha-actinin-1	83.85	0	14		79 800		MSC only
O43707	ACTN4_HUMAN	Alpha-actinin-4	78.19	2	9	10 700	23 900	-2.23	Down in CPC
O14672	ADA10_HUMAN	Disintegrin and metalloproteinase domain-containing protein 10	152.88	12	15	123 000	509 000	-4.14	Down in CPC
P78536	ADA17_HUMAN	Disintegrin and metalloproteinase domain-containing protein 17	152.65	7	10	250 000	174 000	1.44	No change
P07550	ADRB2_HUMAN	Beta-2 adrenergic receptor	47	1	5		34 000		MSC only
O14514	AGRB1_HUMAN	Adhesion G protein-coupled receptor B1	63.23	1	2		26 900		MSC only
O95490	AGRL2_HUMAN	Adhesion G protein-coupled receptor L2	70.25	3	1	680 000			CPC only
Q9UM73	ALK_HUMAN	ALK tyrosine kinase receptor	67.02	4	1	35 100	0		CPC only
P15144	AMPN_HUMAN	Aminopeptidase N	434.11	69	53	149 000 000	67 700 000	2.20	Up in CPC
P30533	AMRP_HUMAN	Alpha-2-macroglobulin receptor-associated protein	67.55	10	4	86 600	50 300	1.72	Up in CPC
P20594	ANPRB_HUMAN	Atrial natriuretic peptide receptor 2	150.62	9	11	1 780 000	163 000	10.92	Up in CPC
P17342	ANPRC_HUMAN	Atrial natriuretic peptide receptor 3	141.7	7	23	14 600	886 000	-60.68	Down in CPC
Q9H6X2	ANTR1_HUMAN	Anthrax toxin receptor 1	139.96	11	17	90 500	1 100 000	-12.15	Down in CPC
P58335	ANTR2_HUMAN	Anthrax toxin receptor 2	127.95	12	10	329 000	385 000	-1.17	No change
P04083	ANXA1_HUMAN	Annexin A1	168.9	32	29	330 000	246 000	1.34	No change
P08133	ANXA6_HUMAN	Annexin A6	47.1	0	3		23 700		MSC only
P04114	APOB_HUMAN	Apolipoprotein B-100	76.18	1	1	13 100	209 000	-15.95	Down in CPC
P02656	APOC3_HUMAN	Apolipoprotein C-III	58.33	16	16	176 000	80 100	2.20	Up in CPC
P02649	APOE_HUMAN	Apolipoprotein E	77.45	15	17	38 200	120 000	-3.14	Down in CPC
Q96P48	ARAP1_HUMAN	Arf-GAP with Rho-GAP domain ANK repeat and PH domain-containing protein 1	32.95	0	1		86 400		MSC only
P16615	AT2A2_HUMAN	Sarcoplasmic/endoplasmic reticulum calcium ATPase 2	100.63	2	6	7 260	16 600	-2.29	Down in CPC
O75882	ATRN_HUMAN	Attractin	40.51	2	1	47 700			MSC only
P50895	BCAM_HUMAN	Basal cell adhesion molecule	227.6	22	36	269 000	1 990 000	-7.40	Down in CPC
P30411	BKRB2_HUMAN	B2 bradykinin receptor	72.37	0	6		61 800		MSC only
P36894	BMR1A_HUMAN	Bone morphogenetic protein receptor type-1A	26.23	0	2		19 000		MSC only
P38398	BRCA1_HUMAN	Breast cancer type 1 susceptibility protein	52.02	1	1	92 700			CPC only
Q7KYR7	BT2A1_HUMAN	Butyrophilin subfamily 2 member A1	81.84	2	13		140 000		MSC only
Q86VB7	C163A_HUMAN	Scavenger receptor cysteine-rich type 1 protein M130	54.39	1	2		28 700		MSC only
Q9BY67	CADM1_HUMAN	Cell adhesion molecule 1	260.09	36	18	5 820 000	234 000	24.87	Up in CPC
P04040	CATA_HUMAN	Catalase	63.05	3	3	0	3 790		MSC only
P48509	CD151_HUMAN	CD151 antigen	34.01	4	4	220 000	220 000	1.00	No change

Table S2. List of proteins classified as *Receptors* based on GO annotations *(continued)*

Accession	Protein ID	Description	-10lgP	Coverage (%) CPC	Coverage (%) MSC	Area CPC	Area MSC	Fold change	Expression
Q13740	CD166_HUMAN	CD166 antigen	355.25	52	55	19 500 000	45 000 000	-2.31	Down in CPC
Q5ZPR3	CD276_HUMAN	CD276 antigen	265.02	26	29	4 720 000	4 130 000	1.14	No change
P16070	CD44_HUMAN	CD44 antigen	293.92	29	27	62 700 000	96 000 000	-1.53	Down in CPC
Q08722	CD47_HUMAN	Leukocyte surface antigen CD47	84.74	9	11	527 000	362 000	1.46	No change
P60033	CD81_HUMAN	CD81 antigen	38.15	8	8	23 900	18 500	1.29	No change
P48960	CD97_HUMAN	CD97 antigen	279.95	18	23	2 560 000	5 760 000	-2.25	Down in CPC
Q9NYQ7	CELR3_HUMAN	Cadherin EGF LAG seven-pass G-type receptor 3	69.85	1	2		361 000		MSC only
Q00610	CLH1_HUMAN	Clathrin heavy chain 1	55.19	0	2		10 300		MSC only
Q99788	CML1_HUMAN	Chemokine-like receptor 1	27.34	0	5		6 100		MSC only
A5YKK6	CNOT1_HUMAN	CCR4-NOT transcription complex subunit 1	71.64	1	2		10 900		MSC only
P23528	COF1_HUMAN	Cofilin-1	149.78	20	46	282 000	1 100 000	-3.90	Down in CPC
Q5KU26	COL12_HUMAN	Collectin-12	121.77	2	8		320 000		MSC only
P35222	CTNB1_HUMAN	Catenin beta-1	57.18	4	3	0			N/A
O60716	CTND1_HUMAN	Catenin delta-1	132.96	11	13	221 000	180 000	1.23	No change
O60494	CUBN_HUMAN	Cubilin	48.45	0	1		28 100		MSC only
P08174	DAF_HUMAN	Complement decay-accelerating factor	144.03	12	12	673 000	675 000	1.00	No change
Q14118	DAG1_HUMAN	Dystroglycan	239.26	23	25	2 120 000	4 000 000	-1.89	Down in CPC
Q16832	DDR2_HUMAN	Discoidin domain-containing receptor 2	239.95	25	30	459 000	1 050 000	-2.29	Down in CPC
Q8N1I0	DOCK4_HUMAN	Dedicator of cytokinesis protein 4	40.37	1	1	0			N/A
P27487	DPP4_HUMAN	Dipeptidyl peptidase 4	282.88	23	39	760 000	10 600 000	-13.95	Down in CPC
Q16610	ECM1_HUMAN	Extracellular matrix protein 1	56.12	5	4	22 800	24 800	-1.09	No change
O43854	EDIL3_HUMAN	EGF-like repeat and discoidin I-like domain-containing protein 3	54.47	1	11	134 000	246 000	-1.84	Down in CPC
P98172	EFNB1_HUMAN	Ephrin-B1	123.08	3	27		118 000		MSC only
P52799	EFNB2_HUMAN	Ephrin-B2	79.39	2	12		59 500		MSC only
Q15768	EFNB3_HUMAN	Ephrin-B3	34.41	4	1	28 800			CPC only
P00533	EGFR_HUMAN	Epidermal growth factor receptor	339.4	33	44	5 790 000	14 900 000	-2.57	Down in CPC
P17813	EGLN_HUMAN	Endoglin	316.98	36	48	2 270 000	14 400 000	-6.34	Down in CPC
P14625	ENPL_HUMAN	Endoplasmin	114.68	10	10	92 500	114 000	-1.23	No change
P22413	ENPP1_HUMAN	Ectonucleotide pyrophosphatase/phosphodiesterase family member 1	263.41	27	35	2 640 000	3 200 000	-1.21	No change
Q9UNN8	EPCR_HUMAN	Endothelial protein C receptor	154.45	29	37	652 000	844 000	-1.29	No change
P29317	EPHA2_HUMAN	Ephrin type-A receptor 2	280.33	19	42	437 000	3 960 000	-9.06	Down in CPC
P29320	EPHA3_HUMAN	Ephrin type-A receptor 3	120.52	10	9	265 000	238 000	1.11	No change
P54764	EPHA4_HUMAN	Ephrin type-A receptor 4	128.81	7	8	756 000	722 000	1.05	No change
P54756	EPHA5_HUMAN	Ephrin type-A receptor 5	169.29	11	14	114 000	147 000	-1.29	No change
Q9UF33	EPHA6_HUMAN	Ephrin type-A receptor 6	100.26	8	4	1 190 000	647 000	1.84	Up in CPC
Q15375	EPHA7_HUMAN	Ephrin type-A receptor 7	113.36	7	8	3 630 000	191 000	19.01	Up in CPC
P29322	EPHA8_HUMAN	Ephrin type-A receptor 8	80.01	4	5	84 000			CPC only
Q5JZY3	EPHAA_HUMAN	Ephrin type-A receptor 10	66.99	6	3	302 000	17 800	16.97	Up in CPC
P54762	EPHB1_HUMAN	Ephrin type-B receptor 1	160.97	12	12	270 000	6 190	43.62	Up in CPC
P29323	EPHB2_HUMAN	Ephrin type-B receptor 2	215.46	20	23	602 000	1 020 000	-1.69	Down in CPC

Table S2. List of proteins classified as *Receptors* based on GO annotations *(continued)*

Accession	Protein ID	Description	-10lgP	Coverage (%) CPC	Coverage (%) MSC	Area CPC	Area MSC	Fold change	Expression
P54753	EPHB3_HUMAN	Ephrin type-B receptor 3	131.56	9	8	4 680	1 980	2.36	Up in CPC
P54760	EPHB4_HUMAN	Ephrin type-B receptor 4	174.55	10	18	62 600	2 140 000	-34.19	Down in CPC
P42566	EPS15_HUMAN	Epidermal growth factor receptor substrate 15	46.09	1	3		60 500		MSC only
Q9NZ08	ERAP1_HUMAN	Endoplasmic reticulum aminopeptidase 1	46.93	2	2	34 600	22 400	1.54	Up in CPC
P04626	ERBB2_HUMAN	Receptor tyrosine-protein kinase erbB-2	166.68	9	13	151 000	521 000	-3.45	Down in CPC
Q15303	ERBB4_HUMAN	Receptor tyrosine-protein kinase erbB-4	120.81	5	9	0	2 520 000		MSC only
P02751	FINC_HUMAN	Fibronectin	365.38	6	40	49 500	14 600 000	-294.95	Down in CPC
P21333	FLNA_HUMAN	Filamin-A	359.24	30	37	4 550 000	7 200 000	-1.58	Down in CPC
O43155	FLRT2_HUMAN	Leucine-rich repeat transmembrane protein FLRT2	71.97	14	0	241 000			CPC only
Q9UP38	FZD1_HUMAN	Frizzled-1	108.95	10	8	338 000	456 000	-1.35	No change
Q14332	FZD2_HUMAN	Frizzled-2	97.24	5	11		67 700		MSC only
O75084	FZD7_HUMAN	Frizzled-7	90.63	5	7		84 400		MSC only
Q9UQC2	GAB2_HUMAN	GRB2-associated-binding protein 2	31.27	2	1	0			N/A
Q7Z4P5	GDF7_HUMAN	Growth/differentiation factor 7	62.73	4	8		0		N/A
Q03113	GNA12_HUMAN	Guanine nucleotide-binding protein subunit alpha-12	81.18	6	15		28 300		MSC only
Q14344	GNA13_HUMAN	Guanine nucleotide-binding protein subunit alpha-13	71.42	10	3	29 700			CPC only
P63096	GNAI1_HUMAN	Guanine nucleotide-binding protein G(i) subunit alpha-1	80.37	8	12	45 300	128 000	-2.83	Down in CPC
P04899	GNAI2_HUMAN	Guanine nucleotide-binding protein G(i) subunit alpha-2	165.16	23	25	179 000	514 000	-2.87	Down in CPC
Q5JWF2	GNAS1_HUMAN	Guanine nucleotide-binding protein G(s) subunit alpha isoforms Xlas	86.5	5	3	33 200	64 900	-1.95	Down in CPC
P19087	GNAT2_HUMAN	Guanine nucleotide-binding protein G(t) subunit alpha-2	67.96	8	3	0			N/A
Q5T848	GP158_HUMAN	Probable G-protein coupled receptor 158	72.85	2	2	147 000			CPC only
Q14439	GP176_HUMAN	G-protein coupled receptor 176	168.75	18	19	735 000	714 000	1.03	No change
Q9NZH0	GPC5B_HUMAN	G-protein coupled receptor family C group 5 member B	36.36	0	3		0		N/A
Q9Y625	GPC6_HUMAN	Glypican-6	140.2	11	14	143 000	78 500	1.82	Up in CPC
Q8WXG9	GPR98_HUMAN	G-protein coupled receptor 98	85.72	1	1		190 000		MSC only
Q96JK4	HIPL1_HUMAN	HHIP-like protein 1	33.12	2	0	31 700			CPC only
P13747	HLAE_HUMAN	HLA class I histocompatibility antigen alpha chain E	158.81	15	23	0	154 000		MSC only
P17693	HLAG_HUMAN	HLA class I histocompatibility antigen alpha chain G	122.29	20	17	77 400	69 800	1.11	No change
P01893	HLAH_HUMAN	Putative HLA class I histocompatibility antigen alpha chain H	229.92	30	41	78 500	6 880	11.41	Up in CPC
P0DMV8	HS71A_HUMAN	Heat shock 70 kDa protein 1A	92.51	8	12	33 000	137 000	-4.15	Down in CPC
P0DMV9	HS71B_HUMAN	Heat shock 70 kDa protein 1B	92.51	8	12	33 000	137 000	-4.15	Down in CPC
P08238	HS90B_HUMAN	Heat shock protein HSP 90-beta	209.01	29	30	268 000	446 000	-1.66	Down in CPC
P11142	HSP7C_HUMAN	Heat shock cognate 71 kDa protein	167.77	15	29	160 000	390 000	-2.44	Down in CPC
Q14627	I13R2_HUMAN	Interleukin-13 receptor subunit alpha-2	128.27	14	1	267 000			CPC only
P05362	ICAM1_HUMAN	Intercellular adhesion molecule 1	258.48	24	46	200 000	3 530 000	-17.65	Down in CPC
P08069	IGF1R_HUMAN	Insulin-like growth factor 1 receptor	107.59	4	6	30 400	120 000	-3.95	Down in CPC
Q9NPH3	IL1AP_HUMAN	Interleukin-1 receptor accessory protein	145.94	18	20	2 090 000	506 000	4.13	Up in CPC
P40189	IL6RB_HUMAN	Interleukin-6 receptor subunit beta	193	22	21	517 000	767 000	-1.48	Down in CPC
P06213	INSR_HUMAN	Insulin receptor	94.09	5	4	114 000	102 000	1.12	No change
P56199	ITA1_HUMAN	Integrin alpha-1	254.73	13	25	1 010 000	3 630 000	-3.59	Down in CPC

Table S2. List of proteins classified as *Receptors* based on GO annotations *(continued)*

Accession	Protein ID	Description	-10lgP	Coverage (%) CPC	Coverage (%) MSC	Area CPC	Area MSC	Fold change	Expression
O75578	ITA10_HUMAN	Integrin alpha-10	165.45	7	14	263 000	557 000	-2.12	Down in CPC
Q9UKX5	ITA11_HUMAN	Integrin alpha-11	312.04	31	36	13 600 000	23 900 000	-1.76	Down in CPC
P17301	ITA2_HUMAN	Integrin alpha-2	317.04	38	32	8 960 000	2 160 000	4.15	Up in CPC
P26006	ITA3_HUMAN	Integrin alpha-3	338.92	43	53	9 950 000	22 500 000	-2.26	Down in CPC
P13612	ITA4_HUMAN	Integrin alpha-4	123.76	5	10	90 300	272 000	-3.01	Down in CPC
P08648	ITA5_HUMAN	Integrin alpha-5	379.98	54	51	22 700 000	23 100 000	-1.02	No change
Q13797	ITA9_HUMAN	Integrin alpha-9	48.76	1	2	1 420 000	1 070 000	1.33	No change
P06756	ITAV_HUMAN	Integrin alpha-V	358.52	58	62	12 500 000	22 600 000	-1.81	Down in CPC
P20702	ITAX_HUMAN	Integrin alpha-X	48.79	0	2		1 600 000		MSC only
P05556	ITB1_HUMAN	Integrin beta-1	334.1	44	44	33 200 000	50 100 000	-1.51	Down in CPC
P05106	ITB3_HUMAN	Integrin beta-3	212.68	10	20	124 000	1 230 000	-9.92	Down in CPC
P18084	ITB5_HUMAN	Integrin beta-5	245.84	31	34	2 820 000	2 720 000	1.04	No change
Q9BX67	JAM3_HUMAN	Junctional adhesion molecule C	118.87	16	17	135 000	390 000	-2.89	Down in CPC
P17252	KPCA_HUMAN	Protein kinase C alpha type	35.13	3	0	7 270			CPC only
P24043	LAMA2_HUMAN	Laminin subunit alpha-2	49.12	1	1	443 000	0		CPC only
Q16787	LAMA3_HUMAN	Laminin subunit alpha-3	76.9	1	2	4 810 000	152 000	31.64	Up in CPC
O15230	LAMA5_HUMAN	Laminin subunit alpha-5	66.25	1	1	89 200			CPC only
P55268	LAMB2_HUMAN	Laminin subunit beta-2	57.33	2	1	27 900			CPC only
P11279	LAMP1_HUMAN	Lysosome-associated membrane glycoprotein 1	55.79	2	8	76 500	87 000	-1.14	No change
P18428	LBP_HUMAN	Lipopolysaccharide-binding protein	39.41	2	4		0		N/A
P01130	LDLR_HUMAN	Low-density lipoprotein receptor	184.56	17	18	540 000	344 000	1.57	Up in CPC
P19256	LFA3_HUMAN	Lymphocyte function-associated antigen 3	69.87	5	5	169 000	365 000	-2.16	Down in CPC
Q969X1	LFG3_HUMAN	Protein lifeguard 3	41.68	5	5	21 600	44 700	-2.07	Down in CPC
Q96FE5	LIGO1_HUMAN	Leucine-rich repeat and immunoglobulin-like domain-containing nogo receptor-interacting protein 1	28.12	1	1	0			N/A
Q9Y4K0	LOXL2_HUMAN	Lysyl oxidase homolog 2	48.04	1	3		3 960		MSC only
Q92633	LPAR1_HUMAN	Lysophosphatidic acid receptor 1	97.12	11	17	228 000	3 500 000	-15.35	Down in CPC
Q8TF66	LRC15_HUMAN	Leucine-rich repeat-containing protein 15	146.61	23	21	500 000	481 000	1.04	No change
Q07954	LRP1_HUMAN	Prolow-density lipoprotein receptor-related protein	435.24	31	27	20 400 000	19 200 000	1.06	No change
P98164	LRP2_HUMAN	Low-density lipoprotein receptor-related protein 2	55.07	1	0	0			N/A
Q96NW7	LRRC7_HUMAN	Leucine-rich repeat-containing protein 7	43.01	1	0		487 000		MSC only
Q5S007	LRRK2_HUMAN	Leucine-rich repeat serine/threonine-protein kinase 2	58.27	1	1	98 800			CPC only
Q8N2S1	LTBP4_HUMAN	Latent-transforming growth factor beta-binding protein 4	59.59	1	1		0		N/A
P08581	MET_HUMAN	Hepatocyte growth factor receptor	209.7	16	24	475 000	1 430 000	-3.01	Down in CPC
Q08431	MFGM_HUMAN	Lactadherin	122.38	6	13	15 000	66 600	-4.44	Down in CPC
P50281	MMP14_HUMAN	Matrix metalloproteinase-14	279.95	47	41	5 830 000	8 110 000	-1.39	No change
P20645	MPRD_HUMAN	Cation-dependent mannose-6-phosphate receptor	101.86	12	19	155 000	401 000	-2.59	Down in CPC
P11717	MPRI_HUMAN	Cation-independent mannose-6-phosphate receptor	279.71	16	19	769 000	1 280 000	-1.66	Down in CPC
P22897	MRC1_HUMAN	Macrophage mannose receptor 1	32.34	0	1		16 100		MSC only
Q96AM1	MRGRF_HUMAN	Mas-related G-protein coupled receptor member F	72.72	7	7	140 000	79 900	1.75	Up in CPC
P35579	MYH9_HUMAN	Myosin-9	293	17	32	313 000	2 600 000	-8.31	Down in CPC

Table S2. List of proteins classified as *Receptors* based on GO annotations *(continued)*

Accession	Protein ID	Description	-10lgP	Coverage (%) CPC	Coverage (%) MSC	Area CPC	Area MSC	Fold change	Expression
P13591	NCAM1_HUMAN	Neural cell adhesion molecule 1	287.44	12	46	493 000	10 900 000	-22.11	Down in CPC
Q9Y6Q9	NCOA3_HUMAN	Nuclear receptor coactivator 3	44.41	2	0	10 600			CPC only
Q15223	NECT1_HUMAN	Nectin-1	96.31	6	12	286 000	101 000	2.83	Up in CPC
Q92692	NECT2_HUMAN	Nectin-2	223.15	12	40	172 000	1 410 000	-8.20	Down in CPC
Q9NQS3	NECT3_HUMAN	Nectin-3	217.22	27	29	12 700 000	2 200 000	5.77	Up in CPC
Q92859	NEO1_HUMAN	Neogenin	113.33	2	6	35 700	57 600	-1.61	Down in CPC
Q14957	NMDE3_HUMAN	Glutamate receptor ionotropic NMDA 2C	35.55	1	1	0			N/A
P35228	NOS2_HUMAN	Nitric oxide synthase inducible	25.82	0	1		11 500		MSC only
Q04721	NOTC2_HUMAN	Neurogenic locus notch homolog protein 2	204.21	8	9	608 000	1 280 000	-2.11	Down in CPC
Q9Y639	NPTN_HUMAN	Neuroplastin	208.4	27	37	6 630 000	7 670 000	-1.16	No change
O14786	NRP1_HUMAN	Neuropilin-1	350.27	53	52	14 200 000	14 700 000	-1.04	No change
O60462	NRP2_HUMAN	Neuropilin-2	176.38	23	18	861 000	737 000	1.17	No change
Q96RD7	PANX1_HUMAN	Pannexin-1	75.23	2	8	25 200	110 000	-4.37	Down in CPC
P07237	PDIA1_HUMAN	Protein disulfide-isomerase	65.5	3	11	9 220	352 000	-38.18	Down in CPC
P16234	PGFRA_HUMAN	Platelet-derived growth factor receptor alpha	234.72	23	18	1 110 000	964 000	1.15	No change
P09619	PGFRB_HUMAN	Platelet-derived growth factor receptor beta	268.7	27	30	2 630 000	5 330 000	-2.03	Down in CPC
P08F94	PKHD1_HUMAN	Fibrocystin	67.53	1	1		48 200		MSC only
P14923	PLAK_HUMAN	Junction plakoglobin	172.09	20	17	959 000	525 000	1.83	Up in CPC
Q9UIW2	PLXA1_HUMAN	Plexin-A1	239.05	20	16	981 000	741 000	1.32	No change
O75051	PLXA2_HUMAN	Plexin-A2	150.23	4	6	17 700	67 400	-3.81	Down in CPC
P51805	PLXA3_HUMAN	Plexin-A3	185.54	7	8	22 900	92 400	-4.03	Down in CPC
O43157	PLXB1_HUMAN	Plexin-B1	56.43	1	1		3 000		MSC only
O15031	PLXB2_HUMAN	Plexin-B2	321.35	27	33	2 950 000	5 420 000	-1.84	Down in CPC
Q9ULL4	PLXB3_HUMAN	Plexin-B3	74.54	2	1	31 200			CPC only
Q9Y4D7	PLXD1_HUMAN	Plexin-D1	249.95	7	20	160 000	1 910 000	-11.94	Down in CPC
P04156	PRIO_HUMAN	Major prion protein	144.6	20	22	1 480 000	955 000	1.55	Up in CPC
Q9Y6C5	PTC2_HUMAN	Protein patched homolog 2	67.38	1	2		0		N/A
Q13308	PTK7_HUMAN	Inactive tyrosine-protein kinase 7	309.11	24	36	1 210 000	9 630 000	-7.96	Down in CPC
P18433	PTPRA_HUMAN	Receptor-type tyrosine-protein phosphatase alpha	149.02	16	14	838 000	699 000	1.20	No change
P23467	PTPRB_HUMAN	Receptor-type tyrosine-protein phosphatase beta	66.7	2	2	363 000			CPC only
P23469	PTPRE_HUMAN	Receptor-type tyrosine-protein phosphatase epsilon	104.65	2	8	102 000	212 000	-2.08	Down in CPC
P10586	PTPRF_HUMAN	Receptor-type tyrosine-protein phosphatase F	70.93	1	4		39 700		MSC only
P23470	PTPRG_HUMAN	Receptor-type tyrosine-protein phosphatase gamma	264.18	13	23	841 000	3 940 000	-4.68	Down in CPC
Q12913	PTPRJ_HUMAN	Receptor-type tyrosine-protein phosphatase eta	200.42	6	12	87 700	523 000	-5.96	Down in CPC
Q15262	PTPRK_HUMAN	Receptor-type tyrosine-protein phosphatase kappa	147.38	7	9	81 500	1 200 000	-14.72	Down in CPC
P28827	PTPRM_HUMAN	Receptor-type tyrosine-protein phosphatase mu	43.39	2	1	19 600			CPC only
P15151	PVR_HUMAN	Poliovirus receptor	118.11	10	11	611 000	980 000	-1.60	Down in CPC
P20338	RAB4A_HUMAN	Ras-related protein Rab-4A	62.69	9	7	0			N/A
Q92930	RAB8B_HUMAN	Ras-related protein Rab-8B	78.59	11	16		6 350		MSC only
Q8NFJ5	RAI3_HUMAN	Retinoic acid-induced protein 3	80.41	4	5	0	78 200		MSC only

Table S2. List of proteins classified as *Receptors* based on GO annotations *(continued)*

Accession	Protein ID	Description	-10lgP	Coverage (%) CPC	Coverage (%) MSC	Area CPC	Area MSC	Fold change	Expression
Q6ZVN8	RGMC_HUMAN	Hemojuvelin	43.99	2	4		0		N/A
Q9Y6N7	ROBO1_HUMAN	Roundabout homolog 1	213.04	15	17	34 600 000	35 000 000	-1.01	No change
Q9HCK4	ROBO2_HUMAN	Roundabout homolog 2	67.36	2	5	4 520 000	4 490 000	1.01	No change
Q8WZ75	ROBO4_HUMAN	Roundabout homolog 4	36.44	1	1	7 430			CPC only
P08922	ROS1_HUMAN	Proto-oncogene tyrosine-protein kinase ROS	79.09	2	2	955 000	588 000	1.62	Up in CPC
Q8WUM9	S20A1_HUMAN	Sodium-dependent phosphate transporter 1	52.67	0	2		46 400		MSC only
Q13275	SEM3F_HUMAN	Semaphorin-3F	39.49	1	1		125 000		MSC only
O75326	SEM7A_HUMAN	Semaphorin-7A	232.19	29	40	1 720 000	2 640 000	-1.53	Down in CPC
Q12884	SEPR_HUMAN	Prolyl endopeptidase FAP	265.84	40	37	3 870 000	6 550 000	-1.69	Down in CPC
P50454	SERPH_HUMAN	Serpin H1	161.59	24	29	590 000	631 000	-1.07	No change
Q9Y566	SHAN1_HUMAN	SH3 and multiple ankyrin repeat domains protein 1	68.28	2	1		0		N/A
P29353	SHC1_HUMAN	SHC-transforming protein 1	49.46	2	6		4 000 000		MSC only
P51532	SMCA4_HUMAN	Transcription activator BRG1	56.7	2	1	32 800			CPC only
Q99523	SORT_HUMAN	Sortilin	81.21	2	3	0	36 800		MSC only
P29508	SPB3_HUMAN	Serpin B3	36.82	0	6		74 900		MSC only
Q13501	SQSTM_HUMAN	Sequestosome-1	39.93	2	3		12 500		MSC only
Q12846	STX4_HUMAN	Syntaxin-4	23.26	6	0	10 200			CPC only
Q9UGT4	SUSD2_HUMAN	Sushi domain-containing protein 2	39.86	1	2		5 210		MSC only
Q9UQP3	TENN_HUMAN	Tenascin-N	52.84	2	1	0			N/A
P22105	TENX_HUMAN	Tenascin-X	67.61	1	1	278 000	5 560	50.00	Up in CPC
P02786	TFR1_HUMAN	Transferrin receptor protein 1	308.46	50	55	7 140 000	13 300 000	-1.86	Down in CPC
P01137	TGFB1_HUMAN	Transforming growth factor beta-1	42.11	1	6		19 400		MSC only
P37173	TGFR2_HUMAN	TGF-beta receptor type-2	66.1	7	4	93 600	107 000	-1.14	Down in CPC
P04216	THY1_HUMAN	Thy-1 membrane glycoprotein	173.3	24	24	3 320 000	7 040 000	-2.12	Down in CPC
Q9Y490	TLN1_HUMAN	Talin-1	268.01	21	29	2 020 000	2 630 000	-1.30	Down in CPC
P25445	TNR6_HUMAN	Tumor necrosis factor receptor superfamily member 6	151.94	21	24	1 410 000	207 000	6.81	Up in CPC
O14763	TR10B_HUMAN	Tumor necrosis factor receptor superfamily member 10B	76.61	5	4	13 000	9 760	1.33	No change
Q9Y2W1	TR150_HUMAN	Thyroid hormone receptor-associated protein 3	52	1	4		34 800		MSC only
P07204	TRBM_HUMAN	Thrombomodulin	29.28	1	3		9 660		MSC only
O14817	TSN4_HUMAN	Tetraspanin-4	119.43	10	24	14 900	75 100	-5.04	Down in CPC
P30530	UFO_HUMAN	Tyrosine-protein kinase receptor UFO	215.35	13	24	420 000	2 060 000	-4.90	Down in CPC
Q9BZM4	ULBP3_HUMAN	UL16-binding protein 3	88.46	25	0	93 600			CPC only
Q03405	UPAR_HUMAN	Urokinase plasminogen activator surface receptor	131.12	16	9	408 000	231 000	1.77	Up in CPC
O75445	USH2A_HUMAN	Usherin	72.07	1	1	9 200			CPC only
P46939	UTRO_HUMAN	Utrophin	90.43	1	3	6 960	686 000	-98.56	Down in CPC
P19320	VCAM1_HUMAN	Vascular cell adhesion protein 1	281.07	3	52	10 500	3 940 000	-375.24	Down in CPC
P35916	VGFR3_HUMAN	Vascular endothelial growth factor receptor 3	61.66	0	3		14 400		MSC only
P04004	VTNC_HUMAN	Vitronectin	88.37	4	4	255 000	234 000	1.09	No change
P55060	XPO2_HUMAN	Exportin-2	72.7	1	3		39 000		MSC only

Table S3. List of proteins classified as *Enzymes* based on GO annotations. Relative expression levels in CPC vs MSC are based on normalised quantitative MS data.

Accession	Protein ID	Description	-10lgP	Coverage (%) CPC	Coverage (%) MSC	Area CPC	Area MSC	Fold change	Expression
P21589	5NTD_HUMAN	5'-nucleotidase	325.74	55	52	6 110 000	6 340 000	-1.04	No change
P17174	AATC_HUMAN	Aspartate aminotransferase cytoplasmic	23.65	1	3		21 400		MSC only
P78363	ABCA4_HUMAN	Retinal-specific ATP-binding cassette transporter	64.81	1	1	390 000			CPC only
Q86UK0	ABCAC_HUMAN	ATP-binding cassette sub-family A member 12	62.3	1	1	359	3 420	-9.53	Down in CPC
Q86UQ4	ABCAD_HUMAN	ATP-binding cassette sub-family A member 13	70.58	1	1	215 000	74 100	2.90	Up in CPC
O95342	ABCBB_HUMAN	Bile salt export pump	63.52	2	2	0			N/A
O60706	ABCC9_HUMAN	ATP-binding cassette sub-family C member 9	52.72	1	1	0			N/A
Q96J66	ABCCB_HUMAN	ATP-binding cassette sub-family C member 11	82.7	11	7	377 000	138 000	2.73	Up in CPC
P12821	ACE_HUMAN	Angiotensin-converting enzyme	146.61	4	15	442 000	1 450 000	-3.28	Down in CPC
Q07912	ACK1_HUMAN	Activated CDC42 kinase 1	51.4	3	1	29 200			CPC only
O14672	ADA10_HUMAN	Disintegrin and metalloproteinase domain-containing protein 10	152.88	12	15	123 000	509 000	-4.14	Down in CPC
P78536	ADA17_HUMAN	Disintegrin and metalloproteinase domain-containing protein 17	152.65	7	10	250 000	174 000	1.44	No change
Q08828	ADCY1_HUMAN	Adenylate cyclase type 1	58.66	2	2	50 100	26 600	1.88	Up in CPC
O60266	ADCY3_HUMAN	Adenylate cyclase type 3	52.83	2	2	17 000	13 300	1.28	No change
P51828	ADCY7_HUMAN	Adenylate cyclase type 7	82.99	4	4	40 700	76 300	-1.87	Down in CPC
O60503	ADCY9_HUMAN	Adenylate cyclase type 9	46.33	0	3		20 100		MSC only
Q3SY69	AL1L2_HUMAN	Mitochondrial 10-formyltetrahydrofolate dehydrogenase	36.47	1	1		0		N/A
Q9UM73	ALK_HUMAN	ALK tyrosine kinase receptor	67.02	4	1	35 100	0		CPC only
P15144	AMPN_HUMAN	Aminopeptidase N	434.11	69	53	149 000 000	67 700 000	2.20	Up in CPC
Q4KMQ2	ANO6_HUMAN	Anoctamin-6	190.41	12	22	799 000	588 000	1.36	No change
P20594	ANPRB_HUMAN	Atrial natriuretic peptide receptor 2	150.62	9	11	1 780 000	163 000	10.92	Up in CPC
P04083	ANXA1_HUMAN	Annexin A1	168.9	32	29	330 000	246 000	1.34	No change
P84077	ARF1_HUMAN	ADP-ribosylation factor 1	23.45	0	6		0		N/A
P61204	ARF3_HUMAN	ADP-ribosylation factor 3	23.45	0	6		0		N/A
P84085	ARF5_HUMAN	ADP-ribosylation factor 5	23.45	0	6		0		N/A
P05089	ARGI1_HUMAN	Arginase-1	96.63	7	8	13 800	29 500	-2.14	Down in CPC
P29374	ARI4A_HUMAN	AT-rich interactive domain-containing protein 4A	54.72	0	3		0		N/A
Q12797	ASPH_HUMAN	Aspartyl/asparaginyl beta-hydroxylase	52.66	1	3		49 000		MSC only
P54707	AT12A_HUMAN	Potassium-transporting ATPase alpha chain 2	106.07	7	8	55 500	224 000	-4.04	Down in CPC
P05023	AT1A1_HUMAN	Sodium/potassium-transporting ATPase subunit alpha-1	277.57	32	31	974 000	1 700 000	-1.75	Down in CPC
P50993	AT1A2_HUMAN	Sodium/potassium-transporting ATPase subunit alpha-2	207.79	16	16	0	278 000		MSC only
P13637	AT1A3_HUMAN	Sodium/potassium-transporting ATPase subunit alpha-3	229.04	19	19	0	71 200		MSC only
Q13733	AT1A4_HUMAN	Sodium/potassium-transporting ATPase subunit alpha-4	166.46	15	14	239 000 000	211 000 000	1.13	No change
P05026	AT1B1_HUMAN	Sodium/potassium-transporting ATPase subunit beta-1	149.44	24	32	417 000	941 000	-2.26	Down in CPC
P54709	AT1B3_HUMAN	Sodium/potassium-transporting ATPase subunit beta-3	223.69	40	57	3 010 000	9 740 000	-3.24	Down in CPC
P16615	AT2A2_HUMAN	Sarcoplasmic/endoplasmic reticulum calcium ATPase 2	100.63	2	6	7 260	16 600	-2.29	Down in CPC
P20020	AT2B1_HUMAN	Plasma membrane calcium-transporting ATPase 1	236.08	16	22	472 000	664 000	-1.41	No change

Table S3. List of proteins classified as *Enzymes* based on GO annotations *(continued)*

Accession	Protein ID	Description	-10lgP	Coverage (%) CPC	Coverage (%) MSC	Area CPC	Area MSC	Fold change	Expression
Q16720	AT2B3_HUMAN	Plasma membrane calcium-transporting ATPase 3	200.04	8	10	0	11 200		MSC only
P23634	AT2B4_HUMAN	Plasma membrane calcium-transporting ATPase 4	249.09	19	22	352 000	14 200 000	-40.34	Down in CPC
P20648	ATP4A_HUMAN	Potassium-transporting ATPase alpha chain 1	118.32	10	10	98 400	3 400 000	-34.55	Down in CPC
O43861	ATP9B_HUMAN	Probable phospholipid-transporting ATPase IIB	42.36	1	1		105 000		MSC only
P11021	BIP_HUMAN	Endoplasmic reticulum chaperone BiP	171.19	16	24	208 000	933 000	-4.49	Down in CPC
P36894	BMR1A_HUMAN	Bone morphogenetic protein receptor type-1A	26.23	0	2		19 000		MSC only
P38398	BRCA1_HUMAN	Breast cancer type 1 susceptibility protein	52.02	1	1	92 700			CPC only
Q10588	BST1_HUMAN	ADP-ribosyl cyclase/cyclic ADP-ribose hydrolase 2	167.69	19	22	75 900	818 000	-10.78	Down in CPC
O43570	CAH12_HUMAN	Carbonic anhydrase 12	56.33	13	6	91 600	0		CPC only
P07384	CAN1_HUMAN	Calpain-1 catalytic subunit	83.59	7	4	52 000	70 200	-1.35	No change
P17655	CAN2_HUMAN	Calpain-2 catalytic subunit	69.06	3	5	6 500	28 900	-4.45	Down in CPC
P04040	CATA_HUMAN	Catalase	63.05	3	3	0	3 790		MSC only
P07339	CATD_HUMAN	Cathepsin D	37.35	7	3	143 000	5 900	24.24	Up in CPC
Q14004	CDK13_HUMAN	Cyclin-dependent kinase 13	48.81	0	2		0		N/A
P04632	CPNS1_HUMAN	Calpain small subunit 1	57.2	12	10	15 100	53 500	-3.54	Down in CPC
P81605	DCD_HUMAN	Dermcidin	110.59	19	47	271 000	784 000	-2.89	Down in CPC
Q16832	DDR2_HUMAN	Discoidin domain-containing receptor 2	239.95	25	30	459 000	1 050 000	-2.29	Down in CPC
Q9BQ39	DDX50_HUMAN	ATP-dependent RNA helicase DDX50	30.19	5	1	184 000			CPC only
Q9H2U1	DHX36_HUMAN	ATP-dependent RNA helicase DHX36	61.68	3	1	17 200			CPC only
Q09013	DMPK_HUMAN	Myotonin-protein kinase	27.79	1	1		122 000		MSC only
P49184	DNSL1_HUMAN	Deoxyribonuclease-1-like 1	161.37	33	32	428 000	382 000	1.12	No change
P27487	DPP4_HUMAN	Dipeptidyl peptidase 4	282.88	23	39	760 000	10 600 000	-13.95	Down in CPC
Q6XUX3	DUSTY_HUMAN	Dual serine/threonine and tyrosine protein kinase	44.16	2	1	0			N/A
P42892	ECE1_HUMAN	Endothelin-converting enzyme 1	247.61	22	32	688 000	4 070 000	-5.92	Down in CPC
P68104	EF1A1_HUMAN	Elongation factor 1-alpha 1	154.86	17	19	219 000	739 000	-3.37	Down in CPC
Q5VTE0	EF1A3_HUMAN	Putative elongation factor 1-alpha-like 3	154.86	17	19	219 000	739 000	-3.37	Down in CPC
P13639	EF2_HUMAN	Elongation factor 2	183.28	13	14	591 000	436 000	1.36	No change
P98172	EFNB1_HUMAN	Ephrin-B1	123.08	3	27		118 000		MSC only
P52799	EFNB2_HUMAN	Ephrin-B2	79.39	2	12		59 500		MSC only
Q15768	EFNB3_HUMAN	Ephrin-B3	34.41	4	1	28 800			CPC only
P49411	EFTU_HUMAN	Elongation factor Tu mitochondrial	45.35	3	1	9 830			CPC only
P00533	EGFR_HUMAN	Epidermal growth factor receptor	339.4	33	44	5 790 000	14 900 000	-2.57	Down in CPC
Q9NZN4	EHD2_HUMAN	EH domain-containing protein 2	44.8	2	5	116 000	98 500	1.18	No change
P22413	ENPP1_HUMAN	Ectonucleotide pyrophosphatase/phosphodiesterase family member 1	263.41	27	35	2 640 000	3 200 000	-1.21	No change
P29317	EPHA2_HUMAN	Ephrin type-A receptor 2	280.33	19	42	437 000	3 960 000	-9.06	Down in CPC
P29320	EPHA3_HUMAN	Ephrin type-A receptor 3	120.52	10	9	265 000	238 000	1.11	No change
P54764	EPHA4_HUMAN	Ephrin type-A receptor 4	128.81	7	8	756 000	722 000	1.05	No change
P54756	EPHA5_HUMAN	Ephrin type-A receptor 5	169.29	11	14	114 000	147 000	-1.29	No change
Q9UF33	EPHA6_HUMAN	Ephrin type-A receptor 6	100.26	8	4	1 190 000	647 000	1.84	Up in CPC
Q15375	EPHA7_HUMAN	Ephrin type-A receptor 7	113.36	7	8	3 630 000	191 000	19.01	Up in CPC
P29322	EPHA8_HUMAN	Ephrin type-A receptor 8	80.01	4	5	84 000			CPC only

Table S3. List of proteins classified as *Enzymes* based on GO annotations *(continued)*

Accession	Protein ID	Description	-10lgP	Coverage (%) CPC	Coverage (%) MSC	Area CPC	Area MSC	Fold change	Expression
Q5JZY3	EPHAA_HUMAN	Ephrin type-A receptor 10	66.99	6	3	302 000	17 800	16.97	Up in CPC
P54762	EPHB1_HUMAN	Ephrin type-B receptor 1	160.97	12	12	270 000	6 190	43.62	Up in CPC
P29323	EPHB2_HUMAN	Ephrin type-B receptor 2	215.46	20	23	602 000	1 020 000	-1.69	Down in CPC
P54753	EPHB3_HUMAN	Ephrin type-B receptor 3	131.56	9	8	4 680	1 980	2.36	Up in CPC
P54760	EPHB4_HUMAN	Ephrin type-B receptor 4	174.55	10	18	62 600	2 140 000	-34.19	Down in CPC
Q9NZ08	ERAP1_HUMAN	Endoplasmic reticulum aminopeptidase 1	46.93	2	2	34 600	22 400	1.54	Up in CPC
P04626	ERBB2_HUMAN	Receptor tyrosine-protein kinase erbB-2	166.68	9	13	151 000	521 000	-3.45	Down in CPC
Q15303	ERBB4_HUMAN	Receptor tyrosine-protein kinase erbB-4	120.81	5	9	0	2 520 000		MSC only
P49327	FAS_HUMAN	Fatty acid synthase	84.71	1	4		78 300		MSC only
P22087	FBRL_HUMAN	rRNA 2'-O-methyltransferase fibrillarin	60.69	9	8	0	18 700		MSC only
P04406	G3P_HUMAN	Glyceraldehyde-3-phosphate dehydrogenase	136.45	27	27	67 700	361 000	-5.33	Down in CPC
Q9UQC2	GAB2_HUMAN	GRB2-associated-binding protein 2	31.27	2	1	0			N/A
P36269	GGT5_HUMAN	Glutathione hydrolase 5 proenzyme	176.59	7	32	1 420 000	897 000	1.58	Up in CPC
Q03113	GNA12_HUMAN	Guanine nucleotide-binding protein subunit alpha-12	81.18	6	15		28 300		MSC only
Q14344	GNA13_HUMAN	Guanine nucleotide-binding protein subunit alpha-13	71.42	10	3	29 700			CPC only
P63096	GNAI1_HUMAN	Guanine nucleotide-binding protein G(i) subunit alpha-1	80.37	8	12	45 300	128 000	-2.83	Down in CPC
P04899	GNAI2_HUMAN	Guanine nucleotide-binding protein G(i) subunit alpha-2	165.16	23	25	179 000	514 000	-2.87	Down in CPC
Q5JWF2	GNAS1_HUMAN	Guanine nucleotide-binding protein G(s) subunit alpha isoforms XLas	86.5	5	3	33 200	64 900	-1.95	Down in CPC
P19087	GNAT2_HUMAN	Guanine nucleotide-binding protein G(t) subunit alpha-2	67.96	8	3	0			N/A
P38646	GRP75_HUMAN	Stress-70 protein mitochondrial	68.38	4	7	41 700	179 000	-4.29	Down in CPC
P0DMV8	HS71A_HUMAN	Heat shock 70 kDa protein 1A	92.51	8	12	33 000	137 000	-4.15	Down in CPC
P0DMV9	HS71B_HUMAN	Heat shock 70 kDa protein 1B	92.51	8	12	33 000	137 000	-4.15	Down in CPC
P07900	HS90A_HUMAN	Heat shock protein HSP 90-alpha	166.57	18	18		77 700		MSC only
P08238	HS90B_HUMAN	Heat shock protein HSP 90-beta	209.01	29	30	268 000	446 000	-1.66	Down in CPC
P54652	HSP72_HUMAN	Heat shock-related 70 kDa protein 2	137.41	14	18	274 000	31 300	8.75	Up in CPC
P17066	HSP76_HUMAN	Heat shock 70 kDa protein 6	83.94	4	12	0	60 200		MSC only
P11142	HSP7C_HUMAN	Heat shock cognate 71 kDa protein	167.77	15	29	160 000	390 000	-2.44	Down in CPC
P60842	IF4A1_HUMAN	Eukaryotic initiation factor 4A-I	58.7	11	5	15 100	13 900	1.09	No change
P08069	IGF1R_HUMAN	Insulin-like growth factor 1 receptor	107.59	4	6	30 400	120 000	-3.95	Down in CPC
P06213	INSR_HUMAN	Insulin receptor	94.09	5	4	114 000	102 000	1.12	No change
P05106	ITB3_HUMAN	Integrin beta-3	212.68	10	20	124 000	1 230 000	-9.92	Down in CPC
O60229	KALRN_HUMAN	Kalirin	58.54	1	1	4 800			CPC only
Q9BVG8	KIFC3_HUMAN	Kinesin-like protein KIFC3	26.57	0	2		26 600		MSC only
P07288	KLK3_HUMAN	Prostate-specific antigen	35.16	0	5		89 700		MSC only
P17252	KPCA_HUMAN	Protein kinase C alpha type	35.13	3	0	7 270			CPC only
P14618	KPYM_HUMAN	Pyruvate kinase PKM	265.16	45	60	1 630 000	2 760 000	-1.69	Down in CPC
P30613	KPYR_HUMAN	Pyruvate kinase PKLR	76.15	3	4		14 700		MSC only
Q9UIQ6	LCAP_HUMAN	Leucyl-cystinyl aminopeptidase	201.12	13	21	295 000	1 860 000	-6.31	Down in CPC
Q8WWI1	LMO7_HUMAN	LIM domain only protein 7	88.4	1	2		17 500		MSC only
Q9Y4K0	LOXL2_HUMAN	Lysyl oxidase homolog 2	48.04	1	3		3 960		MSC only
Q5S007	LRRK2_HUMAN	Leucine-rich repeat serine/threonine-protein kinase 2	58.27	1	1	98 800			CPC only

Table S3. List of proteins classified as *Enzymes* based on GO annotations *(continued)*

Accession	Protein ID	Description	-10lgP	Coverage (%) CPC	Coverage (%) MSC	Area CPC	Area MSC	Fold change	Expression
Q9UPN3	MACF1_HUMAN	Microtubule-actin cross-linking factor 1 isoforms 1/2/3/5	95.38	1	2	80 600	210 000	-2.61	Down in CPC
P27448	MARK3_HUMAN	MAP/microtubule affinity-regulating kinase 3	43.01	2	1	0			N/A
P08581	MET_HUMAN	Hepatocyte growth factor receptor	209.7	16	24	475 000	1 430 000	-3.01	Down in CPC
P50281	MMP14_HUMAN	Matrix metalloproteinase-14	279.95	47	41	5 830 000	8 110 000	-1.39	No change
P51511	MMP15_HUMAN	Matrix metalloproteinase-15	72.2	3	5		42 300		MSC only
P33527	MRP1_HUMAN	Multidrug resistance-associated protein 1	357.42	44	44	11 400 000	6 040 000	1.89	Up in CPC
Q92887	MRP2_HUMAN	Canalicular multispecific organic anion transporter 1	94.25	8	5	25 300	319 000	-12.61	Down in CPC
O15438	MRP3_HUMAN	Canalicular multispecific organic anion transporter 2	209.14	14	14	311 000	1 140 000	-3.67	Down in CPC
O15439	MRP4_HUMAN	Multidrug resistance-associated protein 4	237.13	16	23	2 150 000	6 640 000	-3.09	Down in CPC
Q5T3U5	MRP7_HUMAN	Multidrug resistance-associated protein 7	38.04	1	0	6 470			CPC only
P35580	MYH10_HUMAN	Myosin-10	153.72	8	8	67 100	292 000	-4.35	Down in CPC
Q7Z406	MYH14_HUMAN	Myosin-14	127.07	6	10	2 990 000	1 460 000	2.05	Up in CPC
P35579	MYH9_HUMAN	Myosin-9	293	17	32	313 000	2 600 000	-8.31	Down in CPC
Q13402	MYO7A_HUMAN	Unconventional myosin-VIIa	63.55	2	1	110 000			CPC only
P00387	NB5R3_HUMAN	NADH-cytochrome b5 reductase 3	203.82	30	36	795 000	1 650 000	-2.08	Down in CPC
Q9Y6Q9	NCOA3_HUMAN	Nuclear receptor coactivator 3	44.41	2	0	10 600			CPC only
P29120	NEC1_HUMAN	Neuroendocrine convertase 1	33.59	2	1	154 000			CPC only
Q96PU5	NED4L_HUMAN	E3 ubiquitin-protein ligase NEDD4-like	26.4	1	2		0		N/A
P08473	NEP_HUMAN	Neprilysin	153.62	19	12	353 000	227 000	1.56	Up in CPC
Q92542	NICA_HUMAN	Nicastrin	113.84	7	5	323 000	271 000	1.19	No change
P35228	NOS2_HUMAN	Nitric oxide synthase inducible	25.82	0	1		11 500		MSC only
Q5VST9	OBSCN_HUMAN	Obscurin	93.78	1	1	28 500			CPC only
Q6ZRI0	OTOG_HUMAN	Otogelin	66.02	1	1	106 000			CPC only
Q13219	PAPP1_HUMAN	Pappalysin-1	52.98	1	2		57 200		MSC only
Q92824	PCSK5_HUMAN	Proprotein convertase subtilisin/kexin type 5	39.53	0	1		0		N/A
P07237	PDIA1_HUMAN	Protein disulfide-isomerase	65.5	3	11	9 220	352 000	-38.18	Down in CPC
P30101	PDIA3_HUMAN	Protein disulfide-isomerase A3	47.86	5	7	191 000	80 400	2.38	Up in CPC
O43933	PEX1_HUMAN	Peroxisome biogenesis factor 1	30.95	1	0	42 800			CPC only
P16234	PGFRA_HUMAN	Platelet-derived growth factor receptor alpha	234.72	23	18	1 110 000	964 000	1.15	No change
P09619	PGFRB_HUMAN	Platelet-derived growth factor receptor beta	268.7	27	30	2 630 000	5 330 000	-2.03	Down in CPC
P07205	PGK2_HUMAN	Phosphoglycerate kinase 2	31.75	1	4		10 800		MSC only
Q02809	PLOD1_HUMAN	Procollagen-lysine 2-oxoglutarate 5-dioxygenase 1	43.18	1	4		307 000		MSC only
O00469	PLOD2_HUMAN	Procollagen-lysine 2-oxoglutarate 5-dioxygenase 2	72.27	3	3	5 000	11 100	-2.22	Down in CPC
O14494	PLPP1_HUMAN	Phospholipid phosphatase 1	70.63	0	14		166 000		MSC only
O14495	PLPP3_HUMAN	Phospholipid phosphatase 3	144.64	13	21	480 000	2 050 000	-4.27	Down in CPC
Q6T4P5	PLPR3_HUMAN	Phospholipid phosphatase-related protein type 3	63.9	2	5		0		N/A
P05186	PPBT_HUMAN	Alkaline phosphatase tissue-nonspecific isozyme	112.38	0	14		503 000		MSC only
P62937	PPIA_HUMAN	Peptidyl-prolyl cis-trans isomerase A	107.15	18	30	244 000	673 000	-2.76	Down in CPC
Q06830	PRDX1_HUMAN	Peroxiredoxin-1	100.75	19	24	74 200	217 000	-2.92	Down in CPC
P32119	PRDX2_HUMAN	Peroxiredoxin-2	80.4	11	17		45 400		MSC only
P30041	PRDX6_HUMAN	Peroxiredoxin-6	76	7	21	5 400	38 400	-7.11	Down in CPC

Table S3. List of proteins classified as *Enzymes* based on GO annotations *(continued)*

Accession	Protein ID	Description	-10lgP	Coverage (%) CPC	Coverage (%) MSC	Area CPC	Area MSC	Fold change	Expression
P55786	PSA_HUMAN	Puromycin-sensitive aminopeptidase	91.18	8	10	114 000	220 000	-1.93	Down in CPC
Q13308	PTK7_HUMAN	Inactive tyrosine-protein kinase 7	309.11	24	36	1 210 000	9 630 000	-7.96	Down in CPC
Q12923	PTN13_HUMAN	Tyrosine-protein phosphatase non-receptor type 13	47.7	1	1	0	0		N/A
P18433	PTPRA_HUMAN	Receptor-type tyrosine-protein phosphatase alpha	149.02	16	14	838 000	699 000	1.20	No change
P23467	PTPRB_HUMAN	Receptor-type tyrosine-protein phosphatase beta	66.7	2	2	363 000			CPC only
P23469	PTPRE_HUMAN	Receptor-type tyrosine-protein phosphatase epsilon	104.65	2	8	102 000	212 000	-2.08	Down in CPC
P10586	PTPRF_HUMAN	Receptor-type tyrosine-protein phosphatase F	70.93	1	4		39 700		MSC only
P23470	PTPRG_HUMAN	Receptor-type tyrosine-protein phosphatase gamma	264.18	13	23	841 000	3 940 000	-4.68	Down in CPC
Q12913	PTPRJ_HUMAN	Receptor-type tyrosine-protein phosphatase eta	200.42	6	12	87 700	523 000	-5.96	Down in CPC
Q15262	PTPRK_HUMAN	Receptor-type tyrosine-protein phosphatase kappa	147.38	7	9	81 500	1 200 000	-14.72	Down in CPC
P28827	PTPRM_HUMAN	Receptor-type tyrosine-protein phosphatase mu	43.39	2	1	19 600			CPC only
P22102	PUR2_HUMAN	Trifunctional purine biosynthetic protein adenosine-3	75.48	5	3	5 150 000	0		CPC only
P51153	RAB13_HUMAN	Ras-related protein Rab-13	68.18	5	15		35 500		MSC only
P61106	RAB14_HUMAN	Ras-related protein Rab-14	66.07	17	14	194 000	416 000	-2.14	Down in CPC
Q9H0U4	RAB1B_HUMAN	Ras-related protein Rab-1B	85.67	11	21		173 000		MSC only
Q9BZG1	RAB34_HUMAN	Ras-related protein Rab-34	82.1	12	17	24 100	68 700	-2.85	Down in CPC
Q7Z6P3	RAB44_HUMAN	Ras-related protein Rab-44	65.85	3	3	125 000			CPC only
P20338	RAB4A_HUMAN	Ras-related protein Rab-4A	62.69	9	7	0			N/A
P61006	RAB8A_HUMAN	Ras-related protein Rab-8A	85.36	13	25		23 800		MSC only
Q92930	RAB8B_HUMAN	Ras-related protein Rab-8B	78.59	11	16		6 350		MSC only
P63000	RAC1_HUMAN	Ras-related C3 botulinum toxin substrate 1	38.2	7	7	14 200	15 500	-1.09	No change
P62834	RAP1A_HUMAN	Ras-related protein Rap-1A	46.2	7	14	13 400	5 820	2.30	Up in CPC
P08922	ROS1_HUMAN	Proto-oncogene tyrosine-protein kinase ROS	79.09	2	2	955 000	588 000	1.62	Up in CPC
P23396	RS3_HUMAN	40S ribosomal protein S3	67.95	12	11	46 900	16 600	2.83	Up in CPC
P21817	RYR1_HUMAN	Ryanodine receptor 1	86.43	1	2	67 500	97 800	-1.45	No change
Q92736	RYR2_HUMAN	Ryanodine receptor 2	89.7	1	1	85 900			CPC only
Q12884	SEPR_HUMAN	Prolyl endopeptidase FAP	265.84	40	37	3 870 000	6 550 000	-1.69	Down in CPC
O43175	SERA_HUMAN	D-3-phosphoglycerate dehydrogenase	152.93	11	18	495 000	733 000	-1.48	No change
P51532	SMCA4_HUMAN	Transcription activator BRG1	56.7	2	1	32 800			CPC only
Q13501	SQSTM_HUMAN	Sequestosome-1	39.93	2	3		12 500		MSC only
Q658P3	STEA3_HUMAN	Metalloreductase STEAP3	90.11	10	8	306 000	212 000	1.44	No change
P07814	SYEP_HUMAN	Bifunctional glutamate/proline--tRNA ligase	83.29	2	1	27 700	42 200	-1.52	Down in CPC
Q71U36	TBA1A_HUMAN	Tubulin alpha-1A chain	247.76	42	49		6 130		MSC only
Q9H4B7	TBB1_HUMAN	Tubulin beta-1 chain	115.54	6	7		3 590		MSC only
P68371	TBB4B_HUMAN	Tubulin beta-4B chain	214.46	38	42	20 800	34 700	-1.67	Down in CPC
P07437	TBB5_HUMAN	Tubulin beta chain	227.03	42	48	298 000	762 000	-2.56	Down in CPC
Q9BUF5	TBB6_HUMAN	Tubulin beta-6 chain	184.54	26	28	9 350	54 800	-5.86	Down in CPC
Q3ZCM7	TBB8_HUMAN	Tubulin beta-8 chain	161.41	15	21		454 000		MSC only
A6NNZ2	TBB8L_HUMAN	Tubulin beta-8 chain-like protein LOC260334	155.95	18	18	161 000	299 000	-1.86	Down in CPC
P37173	TGFR2_HUMAN	TGF-beta receptor type-2	66.1	7	4	93 600	107 000	-1.14	No change
P22735	TGM1_HUMAN	Protein-glutamine gamma-glutamyltransferase K	94.29	5	5	23 000	84 000	-3.65	Down in CPC

Table S3. List of proteins classified as *Enzymes* based on GO annotations *(continued)*

Accession	Protein ID	Description	-10lgP	Coverage (%) CPC	Coverage (%) MSC	Area CPC	Area MSC	Fold change	Expression
Q08188	TGM3_HUMAN	Protein-glutamine gamma-glutamyltransferase E	103.08	8	12	95 800	189 000	-1.97	Down in CPC
P10599	THIO_HUMAN	Thioredoxin	95.36	23	23	284 000	394 000	-1.39	No change
Q9UHN6	TMEM2_HUMAN	Cell surface hyaluronidase	89.85	5	6	145 000	154 000	-1.06	No change
Q63HR2	TNS2_HUMAN	Tensin-2	40.66	1	1	50 200	40 500	1.24	No change
Q8NBS9	TXND5_HUMAN	Thioredoxin domain-containing protein 5	74.54	4	6	7 500	10 200	-1.36	No change
Q5T4S7	UBR4_HUMAN	E3 ubiquitin-protein ligase UBR4	143.33	2	4	35 300	197 000	-5.58	Down in CPC
P30530	UFO_HUMAN	Tyrosine-protein kinase receptor UFO	215.35	13	24	420 000	2 060 000	-4.90	Down in CPC
Q9UI12	VATH_HUMAN	V-type proton ATPase subunit H	61.72	1	8		139 000		MSC only
P19320	VCAM1_HUMAN	Vascular cell adhesion protein 1	281.07	3	52	10 500	3 940 000	-375.24	Down in CPC
P35916	VGFR3_HUMAN	Vascular endothelial growth factor receptor 3	61.66	0	3		14 400		MSC only
Q9Y3S1	WNK2_HUMAN	Serine/threonine-protein kinase WNK2	30.74	0	1		0		N/A

Table S4. List of proteins classified as *Extracellular matrix components* based on GO annotations. Relative expression levels in CPC vs MSC are based on normalised quantitative MS data.

Accession	Protein ID	Description	-10lgP	Coverage (%) CPC	Coverage (%) MSC	Area CPC	Area MSC	Fold change	Expression
O00468	AGRIN_HUMAN	Agrin	62.49	1	1	7 910			CPC only
P04114	APOB_HUMAN	Apolipoprotein B-100	76.18	1	1	13 100	209 000	-15.95	Down in CPC
P02656	APOC3_HUMAN	Apolipoprotein C-III	58.33	16	16	176 000	80 100	2.20	Up in CPC
P02649	APOE_HUMAN	Apolipoprotein E	77.45	15	17	38 200	120 000	-3.14	Down in CPC
P07339	CATD_HUMAN	Cathepsin D	37.35	7	3	143 000	5 900	24.24	Up in CPC
Q6YHK3	CD109_HUMAN	CD109 antigen	353.74	43	42	18 100 000	10 900 000	1.66	Up in CPC
O75339	CILP1_HUMAN	Cartilage intermediate layer protein 1	66.63	3	3	374 000	0		CPC only
P02452	CO1A1_HUMAN	Collagen alpha-1(I) chain	182.24	11	27	281 000	1 170 000	-4.16	Down in CPC
P08123	CO1A2_HUMAN	Collagen alpha-2(I) chain	213.92	26	20	160 000	15 800 000	-98.75	Down in CPC
P02458	CO2A1_HUMAN	Collagen alpha-1(II) chain	90.35	4	3	63 900			CPC only
P02461	CO3A1_HUMAN	Collagen alpha-1(III) chain	85.47	5	5	3 290	354 000	-107.60	Down in CPC
P53420	CO4A4_HUMAN	Collagen alpha-4(IV) chain	68.74	2	2	162 000	2 460 000	-15.19	Down in CPC
P29400	CO4A5_HUMAN	Collagen alpha-5(IV) chain	71.85	2	1		42 200		MSC only
Q14031	CO4A6_HUMAN	Collagen alpha-6(IV) chain	35.98	1	1		143 000		MSC only
P20908	CO5A1_HUMAN	Collagen alpha-1(V) chain	71.95	2	2		64 300		MSC only
P12109	CO6A1_HUMAN	Collagen alpha-1(VI) chain	211.14	20	22	1 190 000	900 000	1.32	No change
P12110	CO6A2_HUMAN	Collagen alpha-2(VI) chain	136.44	6	9	117 000	1 070 000	-9.15	Down in CPC
P12111	CO6A3_HUMAN	Collagen alpha-3(VI) chain	337.92	26	33	2 100 000	6 340 000	-3.02	Down in CPC
A6NMZ7	CO6A6_HUMAN	Collagen alpha-6(VI) chain	79.6	2	3	136 000	0		CPC only
Q02388	CO7A1_HUMAN	Collagen alpha-1(VII) chain	96.61	3	2	22 100	20 500	1.08	No change
Q14055	CO9A2_HUMAN	Collagen alpha-2(IX) chain	68.57	5	5		27 700		Down in CPC
Q99715	COCA1_HUMAN	Collagen alpha-1(XII) chain	77.72	2	1	0			N/A
P39060	COIA1_HUMAN	Collagen alpha-1(XVIII) chain	58.97	2	2		0		N/A
Q14993	COJA1_HUMAN	Collagen alpha-1(XIX) chain	65.15	3	3		23 500		MSC only
Q5KU26	COL12_HUMAN	Collectin-12	121.77	2	8		320 000		MSC only
Q8NFW1	COMA1_HUMAN	Collagen alpha-1(XXII) chain	75.13	3	3		0		N/A
Q86Y22	CONA1_HUMAN	Collagen alpha-1(XXIII) chain	57.98	4	3	10 200			CPC only
Q17RW2	COOA1_HUMAN	Collagen alpha-1(XXIV) chain	62.89	2	2		10 900		MSC only
Q9BXS0	COPA1_HUMAN	Collagen alpha-1(XXV) chain	55.7	6	4	18 600 000	12 400 000	1.50	Up in CPC
Q8IZC6	CORA1_HUMAN	Collagen alpha-1(XXVII) chain	73.57	3	2	135 000			CPC only
Q6UVK1	CSPG4_HUMAN	Chondroitin sulfate proteoglycan 4	444.51	48	52	38 200 000	49 400 000	-1.29	No change
Q14118	DAG1_HUMAN	Dystroglycan	239.26	23	25	2 120 000	4 000 000	-1.89	Down in CPC
Q16610	ECM1_HUMAN	Extracellular matrix protein 1	56.12	5	4	22 800	24 800	-1.09	No change
O43854	EDIL3_HUMAN	EGF-like repeat and discoidin I-like domain-containing protein 3	54.47	1	11	134 000	246 000	-1.84	Down in CPC
P15502	ELN_HUMAN	Elastin	49.56	5	2	33 900	1 790	18.94	Up in CPC

Table S4. List of proteins classified as *Extracellular matrix components* based on GO annotations *(continued)*

Accession	Protein ID	Description	-10lgP	Coverage (%) CPC	Coverage (%) MSC	Area CPC	Area MSC	Fold change	Expression
P14625	ENPL_HUMAN	Endoplasmin	114.68	10	10	92 500	114 000	-1.23	No change
P20930	FILA_HUMAN	Filaggrin	72.44	2	0	34 900			CPC only
P02751	FINC_HUMAN	Fibronectin	365.38	6	40	49 500	14 600 000	-294.95	Down in CPC
Q4ZHG4	FNDC1_HUMAN	Fibronectin type III domain-containing protein 1	65.19	1	3		0		N/A
Q5SZK8	FREM2_HUMAN	FRAS1-related extracellular matrix protein 2	62.17	1	1		0		N/A
P35052	GPC1_HUMAN	Glypican-1	206.21	25	32	1 770 000	1 570 000	1.13	No change
Q9Y625	GPC6_HUMAN	Glypican-6	140.2	11	14	143 000	78 500	1.82	Up in CPC
Q8NDA2	HMCN2_HUMAN	Hemicentin-2	85.98	1	2		29 600		MSC only
P05362	ICAM1_HUMAN	Intercellular adhesion molecule 1	258.48	24	46	200 000	3 530 000	-17.65	Down in CPC
A6NGN9	IGLO5_HUMAN	IgLON family member 5	36.48	3	1	0			N/A
P19827	ITIH1_HUMAN	Inter-alpha-trypsin inhibitor heavy chain H1	38.7	1	2		57 600		MSC only
P14618	KPYM_HUMAN	Pyruvate kinase PKM	265.16	45	60	1 630 000	2 760 000	-1.69	Down in CPC
P24043	LAMA2_HUMAN	Laminin subunit alpha-2	49.12	1	1	443 000	0		CPC only
Q16787	LAMA3_HUMAN	Laminin subunit alpha-3	76.9	1	2	4 810 000	152 000	31.64	Up in CPC
O15230	LAMA5_HUMAN	Laminin subunit alpha-5	66.25	1	1	89 200			CPC only
P55268	LAMB2_HUMAN	Laminin subunit beta-2	57.33	2	1	27 900			CPC only
Q13751	LAMB3_HUMAN	Laminin subunit beta-3	60.63	2	2		13 900		MSC only
P09382	LEG1_HUMAN	Galectin-1	179.48	44	63	896 000	1 510 000	-1.69	Down in CPC
Q96FE5	LIGO1_HUMAN	Leucine-rich repeat and immunoglobulin-like domain-containing nogo receptor-interacting protein 1	28.12	1	1	0			N/A
Q9Y4K0	LOXL2_HUMAN	Lysyl oxidase homolog 2	48.04	1	3		3 960		MSC only
Q9NS15	LTBP3_HUMAN	Latent-transforming growth factor beta-binding protein 3	42.6	1	1	17 500			CPC only
Q8N2S1	LTBP4_HUMAN	Latent-transforming growth factor beta-binding protein 4	59.59	1	1		0		N/A
Q08431	MFGM_HUMAN	Lactadherin	122.38	6	13	15 000	66 600	-4.44	Down in CPC
P43121	MUC18_HUMAN	Cell surface glycoprotein MUC18	295.81	33	50	1 630 000	10 700 000	-6.56	Down in CPC
Q7Z5P9	MUC19_HUMAN	Mucin-19	85.01	1	1	5 160	0		CPC only
Q9NR99	MXRA5_HUMAN	Matrix-remodeling-associated protein 5	58.03	1	1		190 000		MSC only
Q68BL7	OLM2A_HUMAN	Olfactomedin-like protein 2A	35.61	0	4		71 900		MSC only
P34741	SDC2_HUMAN	Syndecan-2	131.24	7	10	167 000	300 000	-1.80	Down in CPC
O75056	SDC3_HUMAN	Syndecan-3	41.84	5	0	17 200			CPC only
Q9BZZ2	SN_HUMAN	Sialoadhesin	64.74	2	1	0			N/A
P50452	SPB8_HUMAN	Serpin B8	39.34	4	3	18 600			CPC only
P50453	SPB9_HUMAN	Serpin B9	33.77	0	7		0		N/A
Q9UKZ4	TEN1_HUMAN	Teneurin-1	40.94	1	0	0			N/A
Q9UQP3	TENN_HUMAN	Tenascin-N	52.84	2	1	0			N/A
P22105	TENX_HUMAN	Tenascin-X	67.61	1	1	278 000	5 560	50.00	Up in CPC
P13726	TF_HUMAN	Tissue factor	148.08	28	43	75 900	718 000	-9.46	Down in CPC
P01137	TGFB1_HUMAN	Transforming growth factor beta-1	42.11	1	6		19 400		MSC only
P04216	THY1_HUMAN	Thy-1 membrane glycoprotein	173.3	24	24	3 320 000	7 040 000	-2.12	Down in CPC
P04004	VTNC_HUMAN	Vitronectin	88.37	4	4	255 000	234 000	1.09	No change

Table S5. List of proteins classified as *Adhesion/junction/cytoskeletal* based on GO annotations. Relative expression levels in CPC vs MSC are based on normalised quantitative MS data.

Accession	Protein ID	Description	-10lgP	Coverage (%) CPC	Coverage (%) MSC	Area CPC	Area MSC	Fold change	Expression
P31946	1433B_HUMAN	14-3-3 protein beta/alpha	95.54	25	12	347 000	14 800	23.45	Up in CPC
P62258	1433E_HUMAN	14-3-3 protein epsilon	106.17	13	12	0	109 000		MSC only
P27348	1433T_HUMAN	14-3-3 protein theta	145.99	36	36	38 000	162 000	-4.26	Down in CPC
P63104	1433Z_HUMAN	14-3-3 protein zeta/delta	120.08	26	32	254 000	95 100	2.67	Up in CPC
P62736	ACTA_HUMAN	Actin aortic smooth muscle	205.27	27	38	544 000	1 340 000	-2.46	Down in CPC
P60709	ACTB_HUMAN	Actin cytoplasmic 1	271.56	44	59	1 850 000	4 000 000	-2.16	Down in CPC
Q9BYX7	ACTBM_HUMAN	Putative beta-actin-like protein 3	150.93	16	9	0			N/A
P63261	ACTG_HUMAN	Actin cytoplasmic 2	271.56	44	59	1 850 000	4 000 000	-2.16	Down in CPC
P12814	ACTN1_HUMAN	Alpha-actinin-1	83.85	0	14		79 800		MSC only
O43707	ACTN4_HUMAN	Alpha-actinin-4	78.19	2	9	10 700	23 900	-2.23	Down in CPC
O14672	ADA10_HUMAN	Disintegrin and metalloproteinase domain-containing protein 10	152.88	12	15	123 000	509 000	-4.14	Down in CPC
P78536	ADA17_HUMAN	Disintegrin and metalloproteinase domain-containing protein 17	152.65	7	10	250 000	174 000	1.44	No change
O14514	AGRB1_HUMAN	Adhesion G protein-coupled receptor B1 2	63.23	1	2		26 900		MSC only
Q09666	AHNK_HUMAN	Neuroblast differentiation-associated protein AHNAK	365.81	26	27	8 410 000	8 950 000	-1.06	No change
P16157	ANK1_HUMAN	Ankyrin-1	61.03	2	1	0			N/A
Q01484	ANK2_HUMAN	Ankyrin-2	82.07	2	1	0			N/A
Q12955	ANK3_HUMAN	Ankyrin-3	83.96	1	1	54 700			CPC only
P04083	ANXA1_HUMAN	Annexin A1	168.9	32	29	330 000	246 000	1.34	No change
P07355	ANXA2_HUMAN	Annexin A2	271.24	52	49	4 650 000	7 470 000	-1.61	Down in CPC
P08133	ANXA6_HUMAN	Annexin A6	47.1	0	3		23 700		MSC only
P84077	ARF1_HUMAN	ADP-ribosylation factor 1	23.45	0	6		0		N/A
P04920	B3A2_HUMAN	Anion exchange protein 2	258.05	27	24	1 320 000	1 100 000	1.20	No change
P35613	BASI_HUMAN	Basigin	277.27	37	42	12 900 000	22 000 000	-1.71	Down in CPC
P11021	BIP_HUMAN	Endoplasmic reticulum chaperone BiP	171.19	16	24	208 000	933 000	-4.49	Down in CPC
Q9UPA5	BSN_HUMAN	Protein bassoon	81.03	2	1	58 800	11 700	5.03	Up in CPC
Q8TCZ2	C99L2_HUMAN	CD99 antigen-like protein 2	173.77	48	49	1 440 000	1 830 000	-1.27	No change
P55287	CAD11_HUMAN	Cadherin-11	151.86	9	25	114 000	495 000	-4.34	Down in CPC
P55290	CAD13_HUMAN	Cadherin-13	257.58	30	29	2 830 000	3 070 000	-1.08	No change
P55291	CAD15_HUMAN	Cadherin-15	120.17	2	10	376 000	176 000	2.14	Up in CPC
Q9HBT6	CAD20_HUMAN	Cadherin-20	63.05	3	8	23 500	2 710	8.67	Up in CPC
Q9H251	CAD23_HUMAN	Cadherin-23	69.29	1	1	0			N/A
P19022	CADH2_HUMAN	Cadherin-2	310.42	32	36	5 690 000	9 490 000	-1.67	Down in CPC
P55283	CADH4_HUMAN	Cadherin-4	99.5	4	5	74 100	225 000	-3.04	Down in CPC
Q9BY67	CADM1_HUMAN	Cell adhesion molecule 1	260.09	36	18	5 820 000	234 000	24.87	Up in CPC

Table S5. List of proteins classified as *Adhesion/junction/cytoskeletal* based on GO annotations *(continued)*

Accession	Protein ID	Description	-10lgP	Coverage (%) CPC	Coverage (%) MSC	Area CPC	Area MSC	Fold change	Expression
Q05682	CALD1_HUMAN	Caldesmon	123.44	5	11	183 000	386 000	-2.11	Down in CPC
P07384	CAN1_HUMAN	Calpain-1 catalytic subunit	83.59	7	4	52 000	70 200	-1.35	No change
P17655	CAN2_HUMAN	Calpain-2 catalytic subunit	69.06	3	5	6 500	28 900	-4.45	Down in CPC
P47756	CAPZB_HUMAN	F-actin-capping protein subunit beta	100.53	15	19	36 500	65 100	-1.78	Down in CPC
P04040	CATA_HUMAN	Catalase	63.05	3	3	0	3 790		MSC only
P48509	CD151_HUMAN	CD151 antigen	34.01	4	4	220 000	220 000	1.00	No change
Q13740	CD166_HUMAN	CD166 antigen	355.25	52	55	19 500 000	45 000 000	-2.31	Down in CPC
P16070	CD44_HUMAN	CD44 antigen	293.92	29	27	62 700 000	96 000 000	-1.53	Down in CPC
P13987	CD59_HUMAN	CD59 glycoprotein	150.34	36	36	2 250 000	1 790 000	1.26	No change
P60033	CD81_HUMAN	CD81 antigen	38.15	8	8	23 900	18 500	1.29	No change
P48960	CD97_HUMAN	CD97 antigen	279.95	18	23	2 560 000	5 760 000	-2.25	Down in CPC
P14209	CD99_HUMAN	CD99 antigen	216.11	48	72	1 240 000	3 170 000	-2.56	Down in CPC
Q15517	CDSN_HUMAN	Corneodesmosin	91.55	8	9	96 000	147 000	-1.53	Down in CPC
Q00610	CLH1_HUMAN	Clathrin heavy chain 1	55.19	0	2		10 300		MSC only
P23528	COF1_HUMAN	Cofilin-1	149.78	20	46	282 000	1 100 000	-3.90	Down in CPC
P35221	CTNA1_HUMAN	Catenin alpha-1	259.14	33	40	1 460 000	414 000	3.53	Up in CPC
P26232	CTNA2_HUMAN	Catenin alpha-2	137.43	8	14	0	73 900		MSC only
P35222	CTNB1_HUMAN	Catenin beta-1	57.18	4	3	0			N/A
O60716	CTND1_HUMAN	Catenin delta-1	132.96	11	13	221 000	180 000	1.23	No change
Q6UVK1	CSPG4_HUMAN	Chondroitin sulfate proteoglycan 4	444.51	48	52	38 200 000	49 400 000	-1.29	No change
P21291	CSRP1_HUMAN	Cysteine and glycine-rich protein 1	118.15	11	19	40 100	218 000	-5.44	Down in CPC
Q14118	DAG1_HUMAN	Dystroglycan	239.26	23	25	2 120 000	4 000 000	-1.89	Down in CPC
Q16832	DDR2_HUMAN	Discoidin domain-containing receptor 2	239.95	25	30	459 000	1 050 000	-2.29	Down in CPC
P17661	DESM_HUMAN	Desmin	86.25	8	5	50 700			CPC only
P15924	DESP_HUMAN	Desmoplakin	267.55	20	24	8 220 000	2 500 000	3.29	Up in CPC
P60981	DEST_HUMAN	Destrin	78.36	7	19	10 300	205 000	-19.90	Down in CPC
P11532	DMD_HUMAN	Dystrophin	56.93	1	1	24 700	63 500	-2.57	Down in CPC
P27487	DPP4_HUMAN	Dipeptidyl peptidase 4	282.88	23	39	760 000	10 600 000	-13.95	Down in CPC
O43491	E41L2_HUMAN	Band 4.1-like protein 2	47.81	0	2		21 000		MSC only
Q9H329	E41LB_HUMAN	Band 4.1-like protein 4B	27.4	2	0	0			N/A
P68104	EF1A1_HUMAN	Elongation factor 1-alpha 1	154.86	17	19	219 000	739 000	-3.37	Down in CPC
P52799	EFNB2_HUMAN	Ephrin-B2	79.39	2	12		59 500		MSC only
P00533	EGFR_HUMAN	Epidermal growth factor receptor	339.4	33	44	5 790 000	14 900 000	-2.57	Down in CPC
P17813	EGLN_HUMAN	Endoglin	316.98	36	48	2 270 000	14 400 000	-6.34	Down in CPC
Q9NZN4	EHD2_HUMAN	EH domain-containing protein 2	44.8	2	5	116 000	98 500	1.18	No change
P14625	ENPL_HUMAN	Endoplasmin	114.68	10	10	92 500	114 000	-1.23	No change
Q9UNN8	EPCR_HUMAN	Endothelial protein C receptor	154.45	29	37	652 000	844 000	-1.29	No change
P29317	EPHA2_HUMAN	Ephrin type-A receptor 2	280.33	19	42	437 000	3 960 000	-9.06	Down in CPC
P58107	EPIPL_HUMAN	Epiplakin	73.63	1	1	25 100	172 000	-6.85	Down in CPC
Q14517	FAT1_HUMAN	Protocadherin Fat 1	247.82	16	6	4 910 000	1 630 000	3.01	Up in CPC
Q13642	FHL1_HUMAN	Four and a half LIM domains protein 1	44.08	0	7		139 000		MSC only

Table S5. List of proteins classified as *Adhesion/junction/cytoskeletal* based on GO annotations *(continued)*

Accession	Protein ID	Description	-10lgP	Coverage (%) CPC	Coverage (%) MSC	Area CPC	Area MSC	Fold change	Expression
P21333	FLNA_HUMAN	Filamin-A	359.24	30	37	4 550 000	7 200 000	-1.58	Down in CPC
O75369	FLNB_HUMAN	Filamin-B	169.75	9	12	62 400 000	31 200 000	2.00	Up in CPC
Q14315	FLNC_HUMAN	Filamin-C	263.38	13	20	3 100 000	2 070 000	1.50	Up in CPC
O43155	FLRT2_HUMAN	Leucine-rich repeat transmembrane protein FLRT2	71.97	14	0	241 000			CPC only
Q5JV73	FRPD3_HUMAN	FERM and PDZ domain-containing protein 3	68.28	3	2	47 900	16 900	2.83	Up in CPC
Q9UP38	FZD1_HUMAN	Frizzled-1	108.95	10	8	338 000	456 000	-1.35	No change
Q14332	FZD2_HUMAN	Frizzled-2	97.24	5	11		67 700		MSC only
P04406	G3P_HUMAN	Glyceraldehyde-3-phosphate dehydrogenase	136.45	27	27	67 700	361 000	-5.33	Down in CPC
P04921	GLPC_HUMAN	Glycophorin-C	130.51	20	20	447 000	992 000	-2.22	Down in CPC
Q03113	GNA12_HUMAN	Guanine nucleotide-binding protein subunit alpha-12	81.18	6	15		28 300		MSC only
Q14344	GNA13_HUMAN	Guanine nucleotide-binding protein subunit alpha-13	71.42	10	3	29 700			CPC only
P38646	GRP75_HUMAN	Stress-70 protein mitochondrial	68.38	4	7	41 700	179 000	-4.29	Down in CPC
P11166	GTR1_HUMAN	Solute carrier family 2 facilitated glucose transporter member 1	153.25	12	14	638 000	809 000	-1.27	No change
Q9ULI3	HEG1_HUMAN	Protein HEG homolog 1	155.52	11	6	747 000	285 000	2.62	Up in CPC
P0DMV8	HS71A_HUMAN	Heat shock 70 kDa protein 1A	92.51	8	12	33 000	137 000	-4.15	Down in CPC
P0DMV9	HS71B_HUMAN	Heat shock 70 kDa protein 1B	92.51	8	12	33 000	137 000	-4.15	Down in CPC
P11142	HSP7C_HUMAN	Heat shock cognate 71 kDa protein	167.77	15	29	160 000	390 000	-2.44	Down in CPC
P05362	ICAM1_HUMAN	Intercellular adhesion molecule 1	258.48	24	46	200 000	3 530 000	-17.65	Down in CPC
P46940	IQGA1_HUMAN	Ras GTPase-activating-like protein IQGAP1	68.17	1	3		65 700		MSC only
P56199	ITA1_HUMAN	Integrin alpha-1	254.73	13	25	1 010 000	3 630 000	-3.59	Down in CPC
Q9UKX5	ITA11_HUMAN	Integrin alpha-11	312.04	31	36	13 600 000	23 900 000	-1.76	Down in CPC
P17301	ITA2_HUMAN	Integrin alpha-2	317.04	38	32	8 960 000	2 160 000	4.15	Up in CPC
P26006	ITA3_HUMAN	Integrin alpha-3	338.92	43	53	9 950 000	22 500 000	-2.26	Down in CPC
P13612	ITA4_HUMAN	Integrin alpha-4	123.76	5	10	90 300	272 000	-3.01	Down in CPC
P08648	ITA5_HUMAN	Integrin alpha-5	379.98	54	51	22 700 000	23 100 000	-1.02	No change
P23229	ITA6_HUMAN	Integrin alpha-6	278.4	30	38	2 610 000	2 530 000	1.03	No change
Q13683	ITA7_HUMAN	Integrin alpha-7	228.01	3	28	18 000	2 460 000	-136.67	Down in CPC
P11215	ITAM_HUMAN	Integrin alpha-M	48.24	3	0	0			N/A
P06756	ITAV_HUMAN	Integrin alpha-V	358.52	58	62	12 500 000	22 600 000	-1.81	Down in CPC
P05556	ITB1_HUMAN	Integrin beta-1	334.1	44	44	33 200 000	50 100 000	-1.51	Down in CPC
P05106	ITB3_HUMAN	Integrin beta-3	212.68	10	20	124 000	1 230 000	-9.92	Down in CPC
P18084	ITB5_HUMAN	Integrin beta-5	245.84	31	34	2 820 000	2 720 000	1.04	No change
Q9BX67	JAM3_HUMAN	Junctional adhesion molecule C	118.87	16	17	135 000	390 000	-2.89	Down in CPC
O60229	KALRN_HUMAN	Kalirin	58.54	1	1	4 800			CPC only
Q96J84	KIRR1_HUMAN	Kin of IRRE-like protein 1	234.19	37	45	709 000	1 590 000	-2.24	Down in CPC
Q8WWI1	LMO7_HUMAN	LIM domain only protein 7	88.4	1	2		17 500		MSC only
Q07954	LRP1_HUMAN	Prolow-density lipoprotein receptor-related protein 1	435.24	31	27	20 400 000	19 200 000	1.06	No change
Q96NW7	LRRC7_HUMAN	Leucine-rich repeat-containing protein 7	43.01	1	0		487 000		MSC only
Q9UPN3	MACF1_HUMAN	Microtubule-actin cross-linking factor 1 isoforms 1/2/3/5	95.38	1	2	80 600	210 000	-2.61	Down in CPC
P29966	MARCS_HUMAN	Myristoylated alanine-rich C-kinase substrate	76.39	17	15	31 200	29 800	1.05	No change
P50281	MMP14_HUMAN	Matrix metalloproteinase-14	279.95	47	41	5 830 000	8 110 000	-1.39	No change

Table S5. List of proteins classified as *Adhesion/junction/cytoskeletal* based on GO annotations *(continued)*

Accession	Protein ID	Description	-10lgP	Coverage (%) CPC	Coverage (%) MSC	Area CPC	Area MSC	Fold change	Expression
P11717	MPRI_HUMAN	Cation-independent mannose-6-phosphate receptor	279.71	16	19	769 000	1 280 000	-1.66	Down in CPC
O95297	MPZL1_HUMAN	Myelin protein zero-like protein 1	146.56	23	24	107 000	564 000	-5.27	Down in CPC
Q9Y4B5	MTCL1_HUMAN	Microtubule cross-linking factor 1	57.86	1	1	0			N/A
P43121	MUC18_HUMAN	Cell surface glycoprotein MUC18	295.81	33	50	1 630 000	10 700 000	-6.56	Down in CPC
Q96S97	MYADM_HUMAN	Myeloid-associated differentiation marker	54.32	0	12		53 400		MSC only
P35579	MYH9_HUMAN	Myosin-9	293	17	32	313 000	2 600 000	-8.31	Down in CPC
Q9Y2A7	NCKP1_HUMAN	Nck-associated protein 1	37.66	1	1		0		N/A
Q96SB3	NEB2_HUMAN	Neurabin-2	37.56	1	1	65 400			CPC only
Q15223	NECT1_HUMAN	Nectin-1	96.31	6	12	286 000	101 000	2.83	Up in CPC
Q92692	NECT2_HUMAN	Nectin-2	223.15	12	40	172 000	1 410 000	-8.20	Down in CPC
Q9NQS3	NECT3_HUMAN	Nectin-3	217.22	27	29	12 700 000	2 200 000	5.77	Up in CPC
P08473	NEP_HUMAN	Neprilysin	153.62	19	12	353 000	227 000	1.56	Up in CPC
O94856	NFASC_HUMAN	Neurofascin	229.27	21	19	914 000	862 000	1.06	No change
Q6T4R5	NHS_HUMAN	Nance-Horan syndrome protein	46.41	2	1	76 400	5 650	13.52	Up in CPC
Q92542	NICA_HUMAN	Nicastrin	113.84	7	5	323 000	271 000	1.19	No change
P35228	NOS2_HUMAN	Nitric oxide synthase inducible	25.82	0	1		11 500		MSC only
O14786	NRP1_HUMAN	Neuropilin-1	350.27	53	52	14 200 000	14 700 000	-1.04	No change
Q8WX93	PALLD_HUMAN	Palladin	58.06	1	1		6 970		MSC only
O60330	PCDGC_HUMAN	Protocadherin gamma-A12	129.3	6	8	37 100			CPC only
Q9Y6V0	PCLO_HUMAN	Protein piccolo	74.04	1	0	13 800			CPC only
P07237	PDIA1_HUMAN	Protein disulfide-isomerase	65.5	3	11	9 220	352 000	-38.18	Down in CPC
P30101	PDIA3_HUMAN	Protein disulfide-isomerase A3	47.86	5	7	191 000	80 400	2.38	Up in CPC
O15018	PDZD2_HUMAN	PDZ domain-containing protein 2	74.12	1	2		0		N/A
P09619	PGFRB_HUMAN	Platelet-derived growth factor receptor beta	268.7	27	30	2 630 000	5 330 000	-2.03	Down in CPC
Q8IVE3	PKHH2_HUMAN	Pleckstrin homology domain-containing family H member 2	65.56	1	1		0		N/A
Q13835	PKP1_HUMAN	Plakophilin-1	93.46	7	6	108 000	346 000	-3.20	Down in CPC
Q99569	PKP4_HUMAN	Plakophilin-4	49.51	2	0	441 000			CPC only
P14923	PLAK_HUMAN	Junction plakoglobin	172.09	20	17	959 000	525 000	1.83	Up in CPC
Q15149	PLEC_HUMAN	Plectin	122.77	6	8	2 730 000	2 930 000	-1.07	No change
P62937	PPIA_HUMAN	Peptidyl-prolyl cis-trans isomerase A	107.15	18	30	244 000	673 000	-2.76	Down in CPC
Q13308	PTK7_HUMAN	Inactive tyrosine-protein kinase 7	309.11	24	36	1 210 000	9 630 000	-7.96	Down in CPC
Q12923	PTN13_HUMAN	Tyrosine-protein phosphatase non-receptor type 13	47.7	1	1	0	0		N/A
Q12913	PTPRJ_HUMAN	Receptor-type tyrosine-protein phosphatase eta	200.42	6	12	87 700	523 000	-5.96	Down in CPC
Q15262	PTPRK_HUMAN	Receptor-type tyrosine-protein phosphatase kappa	147.38	7	9	81 500	1 200 000	-14.72	Down in CPC
P28827	PTPRM_HUMAN	Receptor-type tyrosine-protein phosphatase mu	43.39	2	1	19 600			CPC only
P15151	PVR_HUMAN	Poliovirus receptor	118.11	10	11	611 000	980 000	-1.60	Down in CPC
P63000	RAC1_HUMAN	Ras-related C3 botulinum toxin substrate 1	38.2	7	7	14 200	15 500	-1.09	No change
Q86UR5	RIMS1_HUMAN	Regulating synaptic membrane exocytosis protein 1	44.31	1	1	12 600			CPC only
Q9UQ26	RIMS2_HUMAN	Regulating synaptic membrane exocytosis protein 2	43.93	3	0	440 000			CPC only
P36578	RL4_HUMAN	60S ribosomal protein L4	49.87	1	7		20 400		MSC only
P23396	RS3_HUMAN	40S ribosomal protein S3	67.95	12	11	46 900	16 600	2.83	Up in CPC

Table S5. List of proteins classified as *Adhesion/junction/cytoskeletal* based on GO annotations *(continued)*

Accession	Protein ID	Description	-10lgP	Coverage (%) CPC	Coverage (%) MSC	Area CPC	Area MSC	Fold change	Expression
P62241	RS8_HUMAN	40S ribosomal protein S8	33.45	0	5		27 500		MSC only
P46781	RS9_HUMAN	40S ribosomal protein S9	20.3	0	4		108 000		MSC only
Q12884	SEPR_HUMAN	Prolyl endopeptidase FAP	265.84	40	37	3 870 000	6 550 000	-1.69	Down in CPC
P19634	SL9A1_HUMAN	Sodium/hydrogen exchanger 1	156.95	19	14	683 000	502 000	1.36	No change
Q08AE8	SPIR1_HUMAN	Protein spire homolog 1	54.36	1	7		154 000		MSC only
P11277	SPTB1_HUMAN	Spectrin beta chain erythrocytic	54.92	1	1	0	1 010 000		MSC only
Q13813	SPTN1_HUMAN	Spectrin alpha chain non-erythrocytic 1	50.77	1	1	10 300	24 300	-2.36	Down in CPC
O15020	SPTN2_HUMAN	Spectrin beta chain non-erythrocytic 2	72.22	2	1	110 000	548 000	-4.98	Down in CPC
Q71U36	TBA1A_HUMAN	Tubulin alpha-1A chain	247.76	42	49		6 130		MSC only
P68371	TBB4B_HUMAN	Tubulin beta-4B chain	214.46	38	42	20 800	34 700	-1.67	Down in CPC
P07437	TBB5_HUMAN	Tubulin beta chain	227.03	42	48	298 000	762 000	-2.56	Down in CPC
Q9UKZ4	TEN1_HUMAN	Teneurin-1	40.94	1	0	0			N/A
Q9HBL0	TENS1_HUMAN	Tensin-1	47.15	0	2		6 490		MSC only
P04216	THY1_HUMAN	Thy-1 membrane glycoprotein	173.3	24	24	3 320 000	7 040 000	-2.12	Down in CPC
Q9Y490	TLN1_HUMAN	Talin-1	268.01	21	29	2 020 000	2 630 000	-1.30	No change
Q9Y4G6	TLN2_HUMAN	Talin-2	146.39	5	6		65 400		MSC only
Q63HR2	TNS2_HUMAN	Tensin-2	40.66	1	1	50 200	40 500	1.24	No change
P67936	TPM4_HUMAN	Tropomyosin alpha-4 chain	152.11	14	35	39 200	371 000	-9.46	Down in CPC
O14817	TSN4_HUMAN	Tetraspanin-4	119.43	10	24	14 900	75 100	-5.04	Down in CPC
Q03405	UPAR_HUMAN	Urokinase plasminogen activator surface receptor	131.12	16	9	408 000	231 000	1.77	Up in CPC
P46939	UTRO_HUMAN	Utrophin	90.43	1	3	6 960	686 000	-98.56	Down in CPC
P18206	VINC_HUMAN	Vinculin	64.09	1	1		0		N/A

Table S6. List of proteins that could not be classified into any of the previous groups *(unclassified)* based on GO annotations. Relative expression levels in CPC vs MSC are based on normalised quantitative MS data.

Accession	Protein ID	Description	-10lgP	Coverage (%) CPC	Coverage (%) MSC	Area CPC	Area MSC	Fold change	Expression
P31947	1433S_HUMAN	14-3-3 protein sigma	90.56	10	11	0			N/A
P30443	1A01_HUMAN	HLA class I histocompatibility antigen A-1 alpha chain	294.53	55	45	110 000	0		CPC only
P04439	1A03_HUMAN	HLA class I histocompatibility antigen A-3 alpha chain	294.66	58	39	857 000			CPC only
P13746	1A11_HUMAN	HLA class I histocompatibility antigen A-11 alpha chain	294.05	53	47		0		N/A
P16188	1A30_HUMAN	HLA class I histocompatibility antigen A-30 alpha chain	289	41	55		129 000		MSC only
P30453	1A34_HUMAN	HLA class I histocompatibility antigen A-34 alpha chain	264.12	38	50		0		N/A
P30455	1A36_HUMAN	HLA class I histocompatibility antigen A-36 alpha chain	294.53	55	45	110 000	0		CPC only
Q09160	1A80_HUMAN	HLA class I histocompatibility antigen A-80 alpha chain	255.09	27	33	48 200			CPC only
P30504	1C04_HUMAN	HLA class I histocompatibility antigen Cw-4 alpha chain	247.81	34	54	3 690	14 800	-4.01	Down in CPC
P30505	1C08_HUMAN	HLA class I histocompatibility antigen Cw-8 alpha chain	244.26	29	48		9 620		MSC only
Q29960	1C16_HUMAN	HLA class I histocompatibility antigen Cw-16 alpha chain	243.81	31	46		0		N/A
Q8N4X5	AF1L2_HUMAN	Actin filament-associated protein 1-like 2	45.26	1	3		0		N/A
Q8IVF2	AHNK2_HUMAN	Protein AHNAK2	86.06	1	1	109 000	596 000	-5.47	Down in CPC
P02768	ALBU_HUMAN	Serum albumin	178.61	10	13	13 400	38 400	-2.87	Down in CPC
Q5JTC6	AMER1_HUMAN	APC membrane recruitment protein 1	55.33	1	2		41 800		MSC only
Q6UB99	ANR11_HUMAN	Ankyrin repeat domain-containing protein 11	70.43	2	1	59 400	0		CPC only
Q8N1W1	ARG28_HUMAN	Rho guanine nucleotide exchange factor 28	68.73	2	2	5 880			CPC only
Q12774	ARHG5_HUMAN	Rho guanine nucleotide exchange factor 5	45.88	1	1	0			N/A
Q8IZT6	ASPM_HUMAN	Abnormal spindle-like microcephaly-associated protein	63.66	1	1	1 400			CPC only
Q5T9A4	ATD3B_HUMAN	ATPase family AAA domain-containing protein 3B	47.41	4	1	0			N/A
O75363	BCAS1_HUMAN	Breast carcinoma-amplified sequence 1	30.63	0	4		1 120 000		MSC only
Q12830	BPTF_HUMAN	Nucleosome-remodeling factor subunit BPTF	64.95	1	2		11 000		MSC only
Q10589	BST2_HUMAN	Bone marrow stromal antigen 2	42.21	3	6		29 400		MSC only
Q86VP6	CAND1_HUMAN	Cullin-associated NEDD8-dissociated protein 1	65.78	0	5		119 000		MSC only
Q5VZK9	CARL1_HUMAN	F-actin-uncapping protein LRRC16A	34.36	2	0	207 000			CPC only
Q9HCU0	CD248_HUMAN	Endosialin	118.48	4	9	122 000	395 000	-3.24	Down in CPC
P08962	CD63_HUMAN	CD63 antigen	82.36	5	5	2 510 000	1 530 000	1.64	Up in CPC
P27701	CD82_HUMAN	CD82 antigen	173.32	21	28	396 000	3 650 000	-9.22	Down in CPC
Q9H5V8	CDCP1_HUMAN	CUB domain-containing protein 1	221.47	8	24	245 000	1 730 000	-7.06	Down in CPC
Q5SW79	CE170_HUMAN	Centrosomal protein of 170 kDa	62.67	2	1	726 000			CPC only
Q6ZU64	CFA65_HUMAN	Cilia- and flagella-associated protein 65	47.42	2	1	0			N/A
Q9H2X0	CHRD_HUMAN	Chordin	44.04	2	2		0		N/A
Q9NY35	CLDN1_HUMAN	Claudin domain-containing protein 1	90.46	15	22	106 000	454 000	-4.28	Down in CPC
Q9H6B4	CLMP_HUMAN	CXADR-like membrane protein	153.03	17	27	153 000	1 090 000	-7.12	Down in CPC
O96005	CLPT1_HUMAN	Cleft lip and palate transmembrane protein 1	49.82	2	1	3 770			CPC only
Q8NE01	CNNM3_HUMAN	Metal transporter CNNM3	70.44	1	10		252 000		MSC only

Table S6. List of proteins that could not be classified into any of the previous groups *(unclassified)* based on GO annotations *(continued)*

Accession	Protein ID	Description	-10lgP	Coverage (%) CPC	Coverage (%) MSC	Area CPC	Area MSC	Fold change	Expression
Q12860	CNTN1_HUMAN	Contactin-1	46.03	1	3		214 000		MSC only
O75128	COBL_HUMAN	Protein cordon-bleu	51.83	3	0	614 000			CPC only
P53621	COPA_HUMAN	Coatomer subunit alpha	82.1	3	8	91 700	115 000	-1.25	No change
P53618	COPB_HUMAN	Coatomer subunit beta	75.5	2	3		0		N/A
Q9BV73	CP250_HUMAN	Centrosome-associated protein CEP250	68.33	1	2		0		N/A
Q96FN4	CPNE2_HUMAN	Copine-2	46.26	1	6		2 620 000		MSC only
P01040	CYTA_HUMAN	Cystatin-A	72.14	31	31	29 500	55 300	-1.87	Down in CPC
Q96PD2	DCBD2_HUMAN	Discoidin CUB and LCCL domain-containing protein 2	260.28	23	30	1 260 000	3 420 000	-2.71	Down in CPC
Q16531	DDB1_HUMAN	DNA damage-binding protein 1	43.27	0	2		0		N/A
Q18PE1	DOK7_HUMAN	Protein Dok-7	46.85	2	3		0		N/A
Q08554	DSC1_HUMAN	Desmocollin-1	63.64	3	2	65 300	147 000	-2.25	Down in CPC
Q02413	DSG1_HUMAN	Desmoglein-1	197.47	13	12	879 000	1 660 000	-1.89	Down in CPC
O14640	DVL1_HUMAN	Segment polarity protein dishevelled homolog DVL-1	63.46	4	4	10 100	0		CPC only
Q14204	DYHC1_HUMAN	Cytoplasmic dynein 1 heavy chain 1	183.68	4	12	370 000	1 560 000	-4.22	Down in CPC
Q8NCM8	DYHC2_HUMAN	Cytoplasmic dynein 2 heavy chain 1	76.61	1	1	15 000			CPC only
A2CJ06	DYTN_HUMAN	Dystrotelin	34.64	3	2	0			N/A
P26641	EF1G_HUMAN	Elongation factor 1-gamma	28.55	0	3		0		N/A
Q9BSW2	EFC4B_HUMAN	EF-hand calcium-binding domain-containing protein 4B	33.41	0	5		61 000		MSC only
Q8WYP5	ELYS_HUMAN	Protein ELYS	47.91	1	1		44 800		MSC only
P54852	EMP3_HUMAN	Epithelial membrane protein 3	82.48	21	13	883 000	1 290 000	-1.46	No change
A0FGR8	ESYT2_HUMAN	Extended synaptotagmin-2	46.17	2	1	0			N/A
Q01844	EWS_HUMAN	RNA-binding protein EWS	80.72	5	4	117 000	179 000	-1.53	Down in CPC
Q9H0X4	F234A_HUMAN	Protein FAM234A	230.77	18	37	485 000	1 780 000	-3.67	Down in CPC
P12259	FA5_HUMAN	Coagulation factor V	90.6	2	2	50 600	47 300	1.07	No change
Q8TDW7	FAT3_HUMAN	Protocadherin Fat 3	77.68	1	1	182 000			CPC only
Q5D862	FILA2_HUMAN	Filaggrin-2	245.29	13	9	1 410 000	1 580 000	-1.12	No change
O95466	FMNL1_HUMAN	Formin-like protein 1	42.49	0	2		6 900		MSC only
Q9P2B2	FPRP_HUMAN	Prostaglandin F2 receptor negative regulator	268.48	7	35	20 500	4 870 000	-237.56	Down in CPC
P54826	GAS1_HUMAN	Growth arrest-specific protein 1	52.82	2	11		62 900		MSC only
Q13439	GOGA4_HUMAN	Golgin subfamily A member 4	63.85	1	1		0		N/A
Q96QA5	GSDMA_HUMAN	Gasdermin-A	71.22	5	9	18 500	74 300	-4.02	Down in CPC
P62805	H4_HUMAN	Histone H4	32.44	15	0	0			N/A
Q58FF7	H90B3_HUMAN	Putative heat shock protein HSP 90-beta-3	151.88	15	16	19 500	0		CPC only
P14866	HNRPL_HUMAN	Heterogeneous nuclear ribonucleoprotein L	70.19	7	7	6 130	214 000	-34.91	Down in CPC
Q86YZ3	HORN_HUMAN	Hornerin	297.79	12	13	1 550 000	2 590 000	-1.67	Down in CPC
Q9H1B7	I2BPL_HUMAN	Interferon regulatory factor 2-binding protein-like	93.17	12	18	56 000	167 000	-2.98	Down in CPC
P48735	IDHP_HUMAN	Isocitrate dehydrogenase [NADP] mitochondrial	83.57	4	5	201 000	55 500	3.62	Up in CPC
Q8IVU1	IGDC3_HUMAN	Immunoglobulin superfamily DCC subclass member 3	36.36	1	3		500 000		MSC only
Q969P0	IGSF8_HUMAN	Immunoglobulin superfamily member 8	190.32	21	21	385 000	605 000	-1.57	Down in CPC
O14498	ISLR_HUMAN	Immunoglobulin superfamily containing leucine-rich repeat protein	29.41	4	0	48 000			CPC only
Q06033	ITIH3_HUMAN	Inter-alpha-trypsin inhibitor heavy chain H3	49.22	1	2		0		N/A

Table S6. List of proteins that could not be classified into any of the previous groups *(unclassified)* based on GO annotations *(continued)*

Accession	Protein ID	Description	-10lgP	Coverage (%) CPC	Coverage (%) MSC	Area CPC	Area MSC	Fold change	Expression
P13645	K1C10_HUMAN	Keratin type I cytoskeletal 10	430.89	85	81	66 500 000	86 800 000	-1.31	No change
Q99456	K1C12_HUMAN	Keratin type I cytoskeletal 12	100.24	11	17	12 500	63 000	-5.04	Down in CPC
P13646	K1C13_HUMAN	Keratin type I cytoskeletal 13	195.2	18	31	190 000	163 000	1.17	No change
P02533	K1C14_HUMAN	Keratin type I cytoskeletal 14	324.16	61	57	4 450 000	5 750 000	-1.29	No change
P19012	K1C15_HUMAN	Keratin type I cytoskeletal 15	181.92	27	30	62 700	206 000	-3.29	Down in CPC
P08779	K1C16_HUMAN	Keratin type I cytoskeletal 16	292.59	59	58	1 600 000	2 010 000	-1.26	No change
Q04695	K1C17_HUMAN	Keratin type I cytoskeletal 17	215.6	47	45	618 000	514 000	1.20	No change
P08727	K1C19_HUMAN	Keratin type I cytoskeletal 19	172.6	24	25	36 800	514 000	-13.97	Down in CPC
Q2M2I5	K1C24_HUMAN	Keratin type I cytoskeletal 24	140	8	7	0			N/A
Q7Z3Z0	K1C25_HUMAN	Keratin type I cytoskeletal 25	130.74	13	17		0		N/A
Q7Z3Y9	K1C26_HUMAN	Keratin type I cytoskeletal 26	96.24	7	7		0		N/A
Q7Z3Y8	K1C27_HUMAN	Keratin type I cytoskeletal 27	174.35	20	19	96 200	1 390 000	-14.45	Down in CPC
Q7Z3Y7	K1C28_HUMAN	Keratin type I cytoskeletal 28	152.52	10	21	6 970	428 000	-61.41	Down in CPC
P35527	K1C9_HUMAN	Keratin type I cytoskeletal 9	520.81	93	90	352 000	766 000	-2.18	Down in CPC
Q14532	K1H2_HUMAN	Keratin type I cuticular Ha2	126.25	13	12	138 000			CPC only
P35908	K22E_HUMAN	Keratin type II cytoskeletal 2 epidermal	418.76	86	85	1 110 000	1 440 000	-1.30	No change
Q01546	K22O_HUMAN	Keratin type II cytoskeletal 2 oral	174.06	14	13	756 000	113	6690.27	Up in CPC
P04264	K2C1_HUMAN	Keratin type II cytoskeletal 1	515.78	77	75	315 000	246 000	1.28	No change
Q7Z794	K2C1B_HUMAN	Keratin type II cytoskeletal 1b	201.39	22	34	260 000	826 000	-3.18	Down in CPC
P12035	K2C3_HUMAN	Keratin type II cytoskeletal 3	180.62	16	23	0	163 000		MSC only
P19013	K2C4_HUMAN	Keratin type II cytoskeletal 4	156.62	13	22	20 600	605 000	-29.37	Down in CPC
P13647	K2C5_HUMAN	Keratin type II cytoskeletal 5	298.25	47	52	4 200 000	6 530 000	-1.55	Down in CPC
P02538	K2C6A_HUMAN	Keratin type II cytoskeletal 6A	275.84	45	50	113 000	193 000	-1.71	Down in CPC
P04259	K2C6B_HUMAN	Keratin type II cytoskeletal 6B	268.76	43	48	0	24 700		MSC only
P48668	K2C6C_HUMAN	Keratin type II cytoskeletal 6C	274.55	41	48		0		N/A
P08729	K2C7_HUMAN	Keratin type II cytoskeletal 7	140.25	12	20	270 000	17 900	15.08	Up in CPC
Q3SY84	K2C71_HUMAN	Keratin type II cytoskeletal 71	118.04	16	16	123 000	52 000	2.37	Up in CPC
Q14CN4	K2C72_HUMAN	Keratin type II cytoskeletal 72	105.61	21	14	1 120 000	173 000	6.47	Up in CPC
Q86Y46	K2C73_HUMAN	Keratin type II cytoskeletal 73	122.46	19	11	251 000			CPC only
Q7RTS7	K2C74_HUMAN	Keratin type II cytoskeletal 74	119.35	18	17	333 000	13 700	24.31	Up in CPC
O95678	K2C75_HUMAN	Keratin type II cytoskeletal 75	204.13	23	32	327 000	505 000	-1.54	Down in CPC
Q8N1N4	K2C78_HUMAN	Keratin type II cytoskeletal 78	198.4	37	29	777 000	1 080 000	-1.39	No change
Q5XKE5	K2C79_HUMAN	Keratin type II cytoskeletal 79	197.85	19	22	129 000	761 000	-5.90	Down in CPC
P05787	K2C8_HUMAN	Keratin type II cytoskeletal 8	154.86	17	18	29 000	49 700	-1.71	Down in CPC
Q14678	KANK1_HUMAN	KN motif and ankyrin repeat domain-containing protein 1	45.24	1	2		0		N/A
Q8IZU9	KIRR3_HUMAN	Kin of IRRE-like protein 3	35.59	3	0	37 700			CPC only
Q5T749	KPRP_HUMAN	Keratinocyte proline-rich protein	171.82	9	22	183 000	163 000	1.12	No change
O76011	KRT34_HUMAN	Keratin type I cuticular Ha4	112.01	10	17		221 000		MSC only
Q92764	KRT35_HUMAN	Keratin type I cuticular Ha5	100.13	11	8	164 000			CPC only
O76013	KRT36_HUMAN	Keratin type I cuticular Ha6	94.04	6	13	0	10 500		MSC only
Q14533	KRT81_HUMAN	Keratin type II cuticular Hb1	121.86	9	9	0	31 800		MSC only

Table S6. List of proteins that could not be classified into any of the previous groups *(unclassified)* based on GO annotations *(continued)*

Accession	Protein ID	Description	-10lgP	Coverage (%) CPC	Coverage (%) MSC	Area CPC	Area MSC	Fold change	Expression
P78385	KRT83_HUMAN	Keratin type II cuticular Hb3	121.86	9	9	0	31 800		MSC only
Q9NSB2	KRT84_HUMAN	Keratin type II cuticular Hb4	162.13	22	29	5 600 000	352 000	15.91	Up in CPC
O43790	KRT86_HUMAN	Keratin type II cuticular Hb6	121.86	9	9	0	31 800		MSC only
O76009	KT33A_HUMAN	Keratin type I cuticular Ha3-I	145.93	27	35	153 000	1 040	147.12	Up in CPC
Q14525	KT33B_HUMAN	Keratin type I cuticular Ha3-II	154.9	17	33		80 200		MSC only
P13473	LAMP2_HUMAN	Lysosome-associated membrane glycoprotein 2	68.42	4	9		67 200		MSC only
O75334	LIPA2_HUMAN	Liprin-alpha-2	54.3	1	1	79 700			CPC only
P50851	LRBA_HUMAN	Lipopolysaccharide-responsive and beige-like anchor protein	64.03	1	1	0			N/A
Q13449	LSAMP_HUMAN	Limbic system-associated membrane protein	142.38	21	12	75 100	280 000	-3.73	Down in CPC
Q86X29	LSR_HUMAN	Lipolysis-stimulated lipoprotein receptor	46.55	2	3		1 740 000		MSC only
P61626	LYSC_HUMAN	Lysozyme C	53.95	8	8	23 500	18 100	1.30	No change
Q96T58	MINT_HUMAN	Msx2-interacting protein	73.17	1	1	45 300	99 300	-2.19	Down in CPC
P15941	MUC1_HUMAN	Mucin-1	42.62	1	2	7 410	32 400	-4.37	Down in CPC
Q9UKN1	MUC12_HUMAN	Mucin-12	58.39	0	0	0			N/A
Q02817	MUC2_HUMAN	Mucin-2	45.88	1	0	17 700			CPC only
Q9BRK3	MXRA8_HUMAN	Matrix remodeling-associated protein 8	122.29	22	24	604 000	757 000	-1.25	No change
P35749	MYH11_HUMAN	Myosin-11	171.95	13	12	1 670 000	1 730 000	-1.04	No change
Q9UKX3	MYH13_HUMAN	Myosin-13	108.62	3	5	0	126 000		MSC only
B0I1T2	MYO1G_HUMAN	Unconventional myosin-Ig	40.59	1	2		0		N/A
Q9NZM1	MYOF_HUMAN	Myoferlin	183.16	12	12	352 000	668 000	-1.90	Down in CPC
Q7Z3B1	NEGR1_HUMAN	Neuronal growth regulator 1	163.76	20	29	2 610 000	683 000	3.82	Up in CPC
P18615	NELFE_HUMAN	Negative elongation factor E	33.92	4	1	0			N/A
Q8N4C6	NIN_HUMAN	Ninein	44.07	1	0	8 540			CPC only
Q8NFA2	NOXO1_HUMAN	NADPH oxidase organizer 1	42.6	1	6		17 300		MSC only
Q9P121	NTRI_HUMAN	Neurotrimin	147.45	7	18	78 700	701 000	-8.91	Down in CPC
P57721	PCBP3_HUMAN	Poly(rC)-binding protein 3	80.6	5	8		264 000		MSC only
Q9HCL0	PCD18_HUMAN	Protocadherin-18	62.34	2	2	3 350	4 110	-1.23	No change
Q9Y5I3	PCDA1_HUMAN	Protocadherin alpha-1	51.66	2	1		38 200		MSC only
Q9UN74	PCDA4_HUMAN	Protocadherin alpha-4	61.03	3	2		0		N/A
Q9Y5E6	PCDB3_HUMAN	Protocadherin beta-3	59.59	4	4		15 800		MSC only
Q9Y5H4	PCDG1_HUMAN	Protocadherin gamma-A1	122.09	7	10	65 100	202 000	-3.10	Down in CPC
Q9Y5H1	PCDG2_HUMAN	Protocadherin gamma-A2	117.71	6	8	233 000	125 000	1.86	Up in CPC
Q9Y5H0	PCDG3_HUMAN	Protocadherin gamma-A3	127.04	7	8	165 000	556 000	-3.37	Down in CPC
Q9Y5G9	PCDG4_HUMAN	Protocadherin gamma-A4	119.33	4	7		0		N/A
Q9Y5G8	PCDG5_HUMAN	Protocadherin gamma-A5	123.87	6	9	0	78 900		MSC only
Q9Y5G7	PCDG6_HUMAN	Protocadherin gamma-A6	135.54	8	11	11 700	57 700	-4.93	Down in CPC
Q9Y5G6	PCDG7_HUMAN	Protocadherin gamma-A7	126.74	6	10	38 900	513 000	-13.19	Down in CPC
Q9Y5G5	PCDG8_HUMAN	Protocadherin gamma-A8	128.07	9	8	122 000	13 300	9.17	Up in CPC
Q9Y5G4	PCDG9_HUMAN	Protocadherin gamma-A9	126.1	6	7	0	72 000		MSC only
Q9Y5H3	PCDGA_HUMAN	Protocadherin gamma-A10	125.6	3	9		11 700		MSC only
Q9Y5H2	PCDGB_HUMAN	Protocadherin gamma-A11	130.4	5	10	232 000	1 350 000	-5.82	Down in CPC

Table S6. List of proteins that could not be classified into any of the previous groups *(unclassified)* based on GO annotations *(continued)*

Accession	Protein ID	Description	-10lgP	Coverage (%) CPC	Coverage (%) MSC	Area CPC	Area MSC	Fold change	Expression
Q9Y5G3	PCDGD_HUMAN	Protocadherin gamma-B1	123.77	6	7	45 600			CPC only
Q9Y5G2	PCDGE_HUMAN	Protocadherin gamma-B2	129.54	8	11	15 200	276 000	-18.16	Down in CPC
Q9Y5G1	PCDGF_HUMAN	Protocadherin gamma-B3	132.11	5	11	15 800	1 620 000	-102.53	Down in CPC
Q9UN71	PCDGG_HUMAN	Protocadherin gamma-B4	120.43	5	7	0			N/A
Q9Y5G0	PCDGH_HUMAN	Protocadherin gamma-B5	120.64	5	10	34 200	309 000	-9.04	Down in CPC
Q9Y5F9	PCDGI_HUMAN	Protocadherin gamma-B6	126.74	5	9	741	142 000	-191.63	Down in CPC
Q9Y5F8	PCDGJ_HUMAN	Protocadherin gamma-B7	119.31	5	5	25 400			CPC only
Q9UN70	PCDGK_HUMAN	Protocadherin gamma-C3	143.32	9	10	31 000	347 000	-11.19	Down in CPC
Q9Y5F7	PCDGL_HUMAN	Protocadherin gamma-C4	136.66	12	11	377 000	8 720	43.23	Up in CPC
Q9Y5F6	PCDGM_HUMAN	Protocadherin gamma-C5	123.56	9	8	94 000	22 000 000	-234.04	Down in CPC
Q9BQ51	PD1L2_HUMAN	Programmed cell death 1 ligand 2	69.8	6	6	8 230	56 400	-6.85	Down in CPC
P41219	PERI_HUMAN	Peripherin	85.48	7	6	13 300			MSC only
O75038	PLCH2_HUMAN	1-phosphatidylinositol 4 5-bisphosphate phosphodiesterase eta-2	50.75	1	2	2 130	0		MSC only
O14939	PLD2_HUMAN	Phospholipase D2	24.38	0	1		0		N/A
O00592	PODXL_HUMAN	Podocalyxin	174.63	11	18	341 000	1 450 000	-4.25	Down in CPC
A5A3E0	POTEF_HUMAN	POTE ankyrin domain family member F	189.08	15	18	324 000	774 000	-2.39	Down in CPC
Q70Z35	PREX2_HUMAN	Phosphatidylinositol 3 4 5-trisphosphate-dependent Rac exchanger 2 protein	45.92	1	1		0		N/A
Q7Z6L0	PRRT2_HUMAN	Proline-rich transmembrane protein 2	32.43	4	0	0			N/A
Q99460	PSMD1_HUMAN	26S proteasome non-ATPase regulatory subunit 1	36.4	1	2		16 900		MSC only
Q13200	PSMD2_HUMAN	26S proteasome non-ATPase regulatory subunit 2	44.83	1	3		49 800		MSC only
Q9UL46	PSME2_HUMAN	Proteasome activator complex subunit 2	57.4	2	11		27 400		MSC only
Q08999	RBL2_HUMAN	Retinoblastoma-like protein 2	49.26	1	2		0		N/A
O95980	RECK_HUMAN	Reversion-inducing cysteine-rich protein with Kazal motifs	228.78	18	8	561 000	984 000	-1.75	Down in CPC
Q5UIP0	RIF1_HUMAN	Telomere-associated protein RIF1	59.87	1	1	403 000			CPC only
P13489	RINI_HUMAN	Ribonuclease inhibitor	104.96	16	11	582 000	139 000	4.19	Up in CPC
Q6ZS17	RIPR1_HUMAN	Rho family-interacting cell polarization regulator 1	70.57	3	2	12 200			CPC only
P62750	RL23A_HUMAN	60S ribosomal protein L23a	51.82	0	8		45 300		MSC only
P83731	RL24_HUMAN	60S ribosomal protein L24	52.32	0	8		83 300		MSC only
Q8TEU7	RPGF6_HUMAN	Rap guanine nucleotide exchange factor 6	53.3	1	2		0		N/A
Q8N2Y8	RUSC2_HUMAN	Iporin	66.95	2	3		32 800		MSC only
Q6SPF0	SAMD1_HUMAN	Atherin	42.64	2	3		1 410 000		MSC only
Q6UWP8	SBSN_HUMAN	Suprabasin	65.94	8	4	211 000	816 000	-3.87	Down in CPC
O14828	SCAM3_HUMAN	Secretory carrier-associated membrane protein 3	23.83	5	5	10 300	7 110	1.45	No change
P18827	SDC1_HUMAN	Syndecan-1	148.4	12	14	154 000	640 000	-4.16	Down in CPC
Q9NRX5	SERC1_HUMAN	Serine incorporator 1	65.59	1	4		39 300		MSC only
P78324	SHPS1_HUMAN	Tyrosine-protein phosphatase non-receptor type substrate 1	223.87	29	31	382 000	1 950 000	-5.10	Down in CPC
Q0VAQ4	SMAGP_HUMAN	Small cell adhesion glycoprotein	81.97	35	35	544 000	455 000	1.20	No change
O94964	SOGA1_HUMAN	Protein SOGA1	38.06	1	1		14 200		MSC only
P0C7V6	SP202_HUMAN	Putative transcription factor SPT20 homolog-like 2	39.71	2	2	56 200	3 850	14.60	Up in CPC
Q96P63	SPB12_HUMAN	Serpin B12	68.9	8	8	106 000	126 000	-1.19	No change
P48594	SPB4_HUMAN	Serpin B4	47.22	6	7	168 000	345 000	-2.05	Down in CPC

Table S6. List of proteins that could not be classified into any of the previous groups *(unclassified)* based on GO annotations *(continued)*

Accession	Protein ID	Description	-10lgP	Coverage (%) CPC	Coverage (%) MSC	Area CPC	Area MSC	Fold change	Expression
Q96JI7	SPTCS_HUMAN	Spatacsin	60.56	1	2		79 400		MSC only
Q16629	SRSF7_HUMAN	Serine/arginine-rich splicing factor 7	130.56	24	21	34 000	106 000	-3.12	Down in CPC
Q16563	SYPL1_HUMAN	Synaptophysin-like protein 1	153.35	24	29	1 960 000	2 760 000	-1.41	No change
Q24JP5	T132A_HUMAN	Transmembrane protein 132A	101.4	6	9	5 950	72 000	-12.10	Down in CPC
Q9P273	TEN3_HUMAN	Teneurin-3	269.19	8	25	2 410 000	5 000 000	-2.07	Down in CPC
Q86V81	THOC4_HUMAN	THO complex subunit 4	31.31	2	3		9 910		MSC only
Q8IVF5	TIAM2_HUMAN	T-lymphoma invasion and metastasis-inducing protein 2	55.79	1	1		6 650		MSC only
Q9H0C3	TM117_HUMAN	Transmembrane protein 117	25.71	6	0	13 800			CPC only
Q4V9L6	TM119_HUMAN	Transmembrane protein 119	179.6	23	21	3 050 000	473 000	6.45	Up in CPC
Q9H813	TM206_HUMAN	Transmembrane protein 206	34.05	2	1	15 200			CPC only
Q92973	TNPO1_HUMAN	Transportin-1	108.46	3	9	39 700	108 000	-2.72	Down in CPC
Q9NXH8	TOR4A_HUMAN	Torsin-4A	60.39	2	7		23 700		MSC only
Q13641	TPBG_HUMAN	Trophoblast glycoprotein	192.82	19	26	903 000	1 470 000	-1.63	Down in CPC
P08582	TRFM_HUMAN	Melanotransferrin	242.02	6	42	71 000	1 500 000	-21.13	Down in CPC
P07477	TRY1_HUMAN	Trypsin-1	118.8	9	13	0	59 700		MSC only
P07478	TRY2_HUMAN	Trypsin-2	92.67	9	7	0			N/A
P35030	TRY3_HUMAN	Trypsin-3	97.05	7	9	8 980	63 100	-7.03	Down in CPC
Q9NNX1	TUFT1_HUMAN	Tuftelin	23.01	1	3		11 300		MSC only
P22314	UBA1_HUMAN	Ubiquitin-like modifier-activating enzyme 1	64.83	2	3	17 200	33 900	-1.97	Down in CPC
P0CG47	UBB_HUMAN	Polyubiquitin-B	122.91	17	21		0		N/A
P0CG48	UBC_HUMAN	Polyubiquitin-C	122.91	6	7		0		N/A
Q92738	US6NL_HUMAN	USP6 N-terminal-like protein	33.3	2	1	7 450			CPC only
Q6EMK4	VASN_HUMAN	Vasorin	232.77	24	24	2 540 000	2 130 000	1.19	No change
Q9HC57	WFDC1_HUMAN	WAP four-disulfide core domain protein 1	26.61	2	7		0		N/A
O60293	ZC3H1_HUMAN	Zinc finger C3H1 domain-containing protein	73.06	1	2	0			N/A

Table S7. List of proteins exported from the PEAKS Studio quantitation module. showing significantly differentially expressed proteins following quantitative LC-MS/MS analysis (cut-off fold change >1.5) utilising the Top 3 peptides from each protein

Acc.	Protein ID	Description	Sign.	Coverage (%)	#Peptides	#Unique	MSC Area	CPC Area	MSC Area (top-3 peptides)	CPC Area (top-3 peptides)	Group Profile (Ratio)
Q13308	PTK7_HUMAN	Inactive tyrosine-protein kinase 7	200	19	13	13	2 850 000	723 000	1 220 000	280 000	-4.36
P17813	EGLN_HUMAN	Endoglin	200	26	9	9	5 820 000	1 140 000	3 100 000	700 000	-4.43
P27487	DPP4_HUMAN	Dipeptidyl peptidase 4	200	16	10	10	2 110 000	421 000	1 240 000	179 000	-6.93
P42892	ECE1_HUMAN	Endothelin-converting enzyme 1	200	16	7	7	1 290 000	386 000	645 000	227 000	-2.84
Q96QD8	S38A2_HUMAN	Sodium-coupled neutral amino acid transporter 2	200	7	2	2	613 000	120 000	613 000	120 000	-5.11
P06756	ITAV_HUMAN	Integrin alpha-V	156.54	48	38	38	10 200 000	5 370 000	1 940 000	798 000	-2.43
P54709	AT1B3_HUMAN	Sodium/potassium-transporting ATPase subunit beta-3	130.95	30	8	8	2 660 000	1 040 000	1 810 000	642 000	-2.82
P08195	4F2_HUMAN	4F2 cell-surface antigen heavy chain	127.26	46	30	30	29 400 000	9 020 000	7 700 000	2 770 000	-2.78
P13591	NCAM1_HUMAN	Neural cell adhesion molecule 1	125.31	9	5	5	807 000	199 000	760 000	172 000	-4.42
Q8IWA5	CTL2_HUMAN	Choline transporter-like protein 2	122.37	11	6	6	1 440 000	197 000	980 000	135 000	-7.26
Q13740	CD166_HUMAN	CD166 antigen	80.61	46	29	29	18 900 000	8 120 000	5 580 000	2 480 000	-2.25
P05106	ITB3_HUMAN	Integrin beta-3	73.81	7	5	4	401 000	67 100	302 000	47 100	-6.41
P00533	EGFR_HUMAN	Epidermal growth factor receptor	72.88	27	21	20	5 970 000	2 740 000	2 510 000	1 110 000	-2.26
Q96PD2	DCBD2_HUMAN	Discoidin CUB and LCCL domain-containing protein 2	69.37	14	6	6	966 000	553 000	614 000	372 000	-1.65
Q92633	LPAR1_HUMAN	Lysophosphatidic acid receptor 1	66.79	8	2	2	179 000	95 300	179 000	95 300	-1.88
P43121	MUC18_HUMAN	Cell surface glycoprotein MUC18	63.99	24	9	9	3 200 000	681 000	1 750 000	336 000	-5.21
O14495	PLPP3_HUMAN	Phospholipid phosphatase 3	61.43	7	2	2	430 000	100 000	430 000	100 000	-4.30
Q13683	ITA7_HUMAN	Integrin alpha-7	58.57	1	1	1	87 000	16 600	87 000	16 600	-5.24
P26006	ITA3_HUMAN	Integrin alpha-3	54.73	29	21	21	6 970 000	3 010 000	2 290 000	1 420 000	-1.61
Q92692	NECT2_HUMAN	Nectin-2	50.43	12	5	5	364 000	110 000	311 000	86 200	-3.61
Q9BY67	CADM1_HUMAN	Cell adhesion molecule 1	49.61	6	1	1	16 700	98 600	16 700	98 600	5.90
P02751	FINC_HUMAN	Fibronectin	48.94	1	1	1	169 000	22 100	169 000	22 100	-7.65
P78324	SHPS1_HUMAN	Tyrosine-protein phosphatase non-receptor type substrate1	47.57	13	4	2	284 000	108 000	284 000	108 000	-2.63
P63261	ACTG_HUMAN	Actin cytoplasmic 2	43.74	43	11	5	1 640 000	807 000	1 120 000	623 000	-1.80
P05362	ICAM1_HUMAN	Intercellular adhesion molecule 1	43.67	8	3	3	569 000	107 000	569 000	107 000	-5.32
P43007	SATT_HUMAN	Neutral amino acid transporter A	43.07	24	8	8	1 140 000	661 000	695 000	433 000	-1.61
Q01650	LAT1_HUMAN	Large neutral amino acids transporter small subunit 1	40.42	11	4	4	1 360 000	374 000	1 230 000	331 000	-3.72
P35052	GPC1_HUMAN	Glypican-1	39.72	9	4	4	424 000	128 000	373 000	102 000	-3.66
P18827	SDC1_HUMAN	Syndecan-1	38.78	12	2	2	202 000	104 000	202 000	104 000	-1.94
P50895	BCAM_HUMAN	Basal cell adhesion molecule	35.87	17	7	7	355 000	103 000	237 000	63 800	-3.71
P02786	TFR1_HUMAN	Transferrin receptor protein 1	34.24	40	23	23	4 640 000	3 120 000	1 030 000	631 000	-1.63
P35579	MYH9_HUMAN	Myosin-9	31.95	5	6	5	245 000	50 800	198 000	36 200	-5.47
Q14439	GP176_HUMAN	G-protein coupled receptor 176	30.67	9	3	3	182 000	64 600	182 000	64 600	-2.82
Q6YHK3	CD109_HUMAN	CD109 antigen	30.45	29	30	30	5 310 000	7 240 000	1 070 000	1 690 000	1.58
P15144	AMPN_HUMAN	Aminopeptidase N	30.21	50	50	50	33 300 000	47 800 000	6 670 000	10 400 000	1.56

Table S7. List of proteins exported from the PEAKS Studio quantitation module. showing significantly differentially expressed proteins *(continued)*

Acc.	Protein ID	Description	Sign.	Coverage (%)	#Peptides	#Unique	MSC Area	CPC Area	MSC Area (top-3 peptides)	CPC Area (top-3 peptides)	Group Profile (Ratio)
Q14118	DAG1_HUMAN	Dystroglycan	29.78	15	9	9	1 690 000	961 000	735 000	467 000	-1.57
Q07954	LRP1_HUMAN	Prolow-density lipoprotein receptor-related protein 1	29.59	19	61	61	8 650 000	7 570 000	1 280 000	844 000	-1.52
P56199	ITA1_HUMAN	Integrin alpha-1	28.86	5	6	6	822 000	350 000	605 000	245 000	-2.47
Q4KMQ2	ANO6_HUMAN	Anoctamin-6	28.82	5	3	3	414 000	99 200	344 000	65 500	-5.25
P27701	CD82_HUMAN	CD82 antigen	26.81	16	3	3	1 010 000	188 000	949 000	176 000	-5.39
P07355	ANXA2_HUMAN	Annexin A2	26.13	42	14	14	2 990 000	1 920 000	1 250 000	830 000	-1.51
P04921	GLPC_HUMAN	Glycophorin-C	25.44	20	2	2	326 000	175 000	307 000	157 000	-1.96
P67936	TPM4_HUMAN	Tropomyosin alpha-4 chain	23.79	14	3	2	109 000	35 100	109 000	35 100	-3.11
Q9H0X4	F234A_HUMAN	Protein FAM234A	23.18	11	4	4	541 000	284 000	522 000	275 000	-1.90
P29317	EPHA2_HUMAN	Ephrin type-A receptor 2	22.97	10	7	7	682 000	217 000	376 000	116 000	-3.24
P17301	ITA2_HUMAN	Integrin alpha-2	22.44	23	17	17	976 000	2 450 000	344 000	915 000	2.66
Q99808	S29A1_HUMAN	Equilibrative nucleoside transporter 1	20.72	7	3	3	105 000	45 800	82 200	33 900	-2.42
